# Differential pheromone profile as a contributor to premating isolation between two sympatric sibling fruit fly species

**DOI:** 10.1093/jisesa/ieae066

**Published:** 2024-06-24

**Authors:** Cynthia Castro-Vargas, John Graham Oakeshott, Heng Lin Yeap, Michael J Lacey, Siu Fai Lee, Soo Jean Park, Phillip Warren Taylor, Gunjan Pandey

**Affiliations:** Environment, Commonwealth Scientific and Industrial Research Organisation, Acton, ACT, Australia; Applied BioSciences, Macquarie University, North Ryde, NSW, Australia; Environment, Commonwealth Scientific and Industrial Research Organisation, Acton, ACT, Australia; Applied BioSciences, Macquarie University, North Ryde, NSW, Australia; Environment, Commonwealth Scientific and Industrial Research Organisation, Acton, ACT, Australia; Health and Biosecurity, Commonwealth Scientific and Industrial Research Organisation, Parkville, VIC, Australia; Bio21 Molecular Science and Biotechnology Institute, University of Melbourne, Parkville, VIC, Australia; National Collections and Marine Infrastructure, Commonwealth Scientific and Industrial Research Organisation, Acton, ACT, Australia; Environment, Commonwealth Scientific and Industrial Research Organisation, Acton, ACT, Australia; Applied BioSciences, Macquarie University, North Ryde, NSW, Australia; Applied BioSciences, Macquarie University, North Ryde, NSW, Australia; Australian Research Council Centre for Fruit Fly Biosecurity Innovation, Macquarie University, North Ryde, NSW, Australia; Applied BioSciences, Macquarie University, North Ryde, NSW, Australia; Australian Research Council Centre for Fruit Fly Biosecurity Innovation, Macquarie University, North Ryde, NSW, Australia; Environment, Commonwealth Scientific and Industrial Research Organisation, Acton, ACT, Australia; Applied BioSciences, Macquarie University, North Ryde, NSW, Australia

**Keywords:** Queensland fruit fly, *Bactrocera tryoni*, *Bactrocera neohumeralis*, rectal gland, sex pheromone

## Abstract

*Bactrocera tryoni* (Froggatt) and *Bactrocera neohumeralis* (Hardy) are sibling fruit fly species that are sympatric over much of their ranges. Premating isolation of these close relatives is thought to be maintained in part by allochrony—mating activity in *B. tryoni* peaks at dusk, whereas in *B. neohumeralis*, it peaks earlier in the day. To ascertain whether differences in pheromone composition may also contribute to premating isolation between them, this study used solid-phase microextraction and gas chromatography-mass spectrometry to characterize the rectal gland volatiles of a recently collected and a more domesticated strain of each species. These glands are typical production sites and reservoirs of pheromones in bactrocerans. A total of 120 peaks were detected and 50 were identified. Differences were found in the composition of the rectal gland emissions between the sexes, species, and recently collected versus domesticated strains of each species. The compositional variation included several presence/absence and many quantitative differences. Species and strain differences in males included several relatively small alcohols, esters, and aliphatic amides. Species and strain differences in females also included some of the amides but additionally involved many fatty acid esters and 3 spiroacetals. While the strain differences indicate there is also heritable variation in rectal gland emissions within each species, the species differences imply that compositional differences in pheromones emitted from rectal glands could contribute to the premating isolation between *B. tryoni* and *B. neohumeralis*. The changes during domestication could also have significant implications for the efficacy of Sterile Insect Technique control programs.

## Introduction

Closely related sympatric insect taxa may remain reproductively isolated through differences in the nature or timing of visual, acoustic, tactile, or chemical signals in premating communication (Muñoz et al. 2010, Takanashi et al. 2010, Veen et al. 2011, Jones and Conner 2018). For example, differences in sex pheromones have been implicated as key factors in maintaining reproductive isolation in moths (Roelofs and Brown 1982, Ming et al. 2007, Allison and Cardé 2016, Liénard and Löfstedt 2019). Some closely related moth species differ in the ratios of geometric or optical isomers of major components of their sex pheromone blends. The moths involved include sibling species in the noctuid genera *Helicoverpa* (Wang et al. 2005, Wu et al. 2013) and *Spodoptera* (Tamaki and Yushima 1974, Muñoz et al. 2008) and the geometrid genera *Itame* (Millar et al. 1990) and *Semiothisa* (Gries et al. 1993). Even when differences in other aspects of the mating system—such as visual displays—have a role, differences in pheromones may still be very important contributors to sexual isolation (Cardé 1986, Wicker-Thomas 2011, Bacquet et al. 2015, Juárez et al. 2015, Liénard and Löfstedt 2019).

The Queensland fruit fly *Bactrocera tryoni* (Froggatt) (Diptera: Tephritidae) and lesser Queensland fruit fly *Bactrocera neohumeralis* (Hardy) (Diptera: Tephritidae) are sibling pest species of horticulture in Australia (Hancock et al. 2000, Clarke et al. 2011). While they occur sympatrically in parts of their ranges, *B. tryoni* is more widespread across eastern and northern Australia, while *B. neohumeralis* is largely confined to the coastline of north-eastern Australia (Hancock and Drew 1999, Hancock et al. 2000, Dominiak and Worsley 2016) and further north in Papua New Guinea (Drew et al. 1978). The only reliable taxonomic difference known between them in morphology is the color of their humeral calli, which is yellow in *B. tryoni* and brown in *B. neohumeralis* (Drew 1989). However, while *B. tryoni* and *B. neohumeralis* can be crossed to produce fertile progeny in the laboratory, microsatellite, and other genetic analyses have so far revealed no clear evidence of hybridization between the 2 species in the field (Smith 1979, Gilchrist and Ling 2006, Yeap et al. 2020). The apparent absence of hybridization in the field is believed to be at least in part due to allochrony; *B. tryoni* mates during a short period at dusk (Tychsen and Fletcher 1971, Tychsen 1978, Smith 1979) whereas *B. neohumeralis* mates in the morning to late afternoon in bright light (Smith 1979, Yeap et al. 2020).

Several studies have investigated sex pheromone functions in *B. tryoni* but very little is known about the sex pheromones of *B. neohumeralis* and nothing is known about how any pheromone differences between them relate to their differences in mating time. When *B. tryoni* males are sexually active at dusk they release a volatile blend from a specialized rectal gland and disperse it by rapid wing fanning (“calling”) (Monro 1953, Fletcher 1969, Tychsen 1978, Mankin et al. 2008). It has been suggested that the volatile blend is a short-range attractant of females and functions as a sex pheromone (Fletcher 1968, Tychsen 1978, Bellas and Fletcher 1979, Kumaran et al. 2014), since mature virgin females respond to volatiles from crushed rectal glands in the laboratory (Giannakakis and Fletcher 1978, Kumaran et al. 2014). Nothing is known of the function of female gland emissions, but female-produced volatiles are known to be involved in the mating system of some other bactroceran fruit flies. For example, females of the olive fruit fly, *Bactrocera oleae*, produce a volatile pheromone that attracts males (Canale et al. 2013, 2015). Some spiroacetals are known to be pheromonal components in the oriental fruit fly *Bactrocera dorsalis* (Baker and Bacon 1985) and elicit antennal responses in both sexes of the mango fruit fly *Bactrocera frauenfeldi* (Noushini et al. 2020b) and in males of the banana fruit fly *Bactrocera musae* (Noushini et al. 2020c).

Several studies have analyzed the volatile contents of *B. tryoni* rectal glands. Most of the work has analyzed solvent-extracted rectal gland contents using gas chromatography–mass spectrometry (GC–MS; Bellas and Fletcher 1979, Lewis 1988, Fletcher and Kitching 1995, Kumaran et al. 2014, Pérez et al. 2018, Noushini et al. 2020a) but other studies have also applied GC–MS to headspace volatiles from dissected glands or intact flies using solvent extractions of adsorbents from dynamic headspace collections or solid phase micro-extraction (SPME) (Booth et al. 2006, Noushini et al. 2020a, Castro-Vargas et al. 2022, 2023). Some quantitative analyses of hexane extracts of rectal gland contents have also been carried out using gas chromatography with a flame ionization detector (GC-FID) (Castro-Vargas et al. 2022). In sum, across all these studies, over 140 chemicals have been detected and over 50 of the more abundant ones have been identified.

The major chemicals in male *B. tryoni* rectal glands are aliphatic amides (methyl or methylbutyl acetamides or propanamides) and these are either not detected or are much less abundant in female rectal glands. Other chemicals identified in the male glands, albeit not necessarily preferentially expressed in male glands, include several short- and long-chain fatty acid esters and short-chain ketones and alcohols. The major compounds identified in female *B. tryoni* rectal glands are various saturated and unsaturated fatty acid esters. In addition, several less abundant spiroacetals identified from female glands have generally not been recorded from male glands (Bellas and Fletcher 1979, Booth et al. 2006, Kumaran et al. 2014, El-Sayed et al. 2019, Noushini et al. 2020a, but see also Castro-Vargas et al. 2022). Several volatiles from the rectal glands of each sex have also been recovered in headspace analyses of whole live flies of that sex, which supports the proposition that some of them may have pheromonal functions (Booth et al. 2006, El-Sayed et al. 2019, Noushini et al. 2020a). However, specific pheromonal functions have not yet been elucidated for any of the individual volatiles recovered from either male or female gland extracts. The only information published to date on the rectal gland semiochemistry of either sex of *B. neohumeralis* is that the 6 major aliphatic amides found in hexane extracts from male *B. tryoni* rectal glands are also found in broadly similar proportions in hexane extracts of male *B. neohumeralis* rectal glands (Bellas and Fletcher 1979, Fletcher and Kitching 1995).

The present study compares the headspace volatile profiles of dissected rectal glands of each sex in *B. tryoni* and *B. neohumeralis* using SPME GC–MS. Given previous evidence that the profiles of *B. tryoni* glands differ markedly between virgins of each sex and those from mixed-sex groups (Castro-Vargas et al. 2022, 2023), which itself is suggestive of a role in mating, this study also analyses the profiles of both virgin and mainly mated flies (i.e., from mixed-sex groups) of each sex and species. Two strains of each species differing in number of generations in the laboratory are used in all comparisons to test whether volatile profiles change during domestication. Preliminary evidence for domestication-related changes in rectal gland volatile production has already been reported for *B. tryoni* (Pérez et al. 2018, Castro-Vargas et al. 2023) and domestication-related changes have also been demonstrated for some other characters (Weldon 2005, Lynch et al. 2018, Popa-Báez et al. 2020, Ahmed et al. 2022, Gaire et al. 2022).

## Materials and Methods

### Fly Stocks and Culture Conditions

Details of the 4 strains used are given in Table 1. The 2 *B. tryoni* strains were collected from the Canberra/Sydney region in southeastern Australia, where that species predominates. The 2 *B. neohumeralis* strains were collected from the Cairns/Mareeba region in north-eastern Australia, where that species is more common. The recently collected and long domesticated strains of each species are hereafter designated “New” and “Old,” respectively. Culture conditions, which were the same for both species, followed those of Castro-Vargas et al. (2023). This included a 13:11 h light:dark photoperiod, with a simulated dawn and dusk in which light levels gradually ramped up and down over a period of 1 h at the beginning and end of the light phase, respectively.

**Table 1.** Provenance of the strains used. Note that the old *B. tryoni* strain corresponds to the S06 stain developed by Gilchrist and Meats (2010) and subsequently also used in studies of the species’ reproductive biology by Ahmed et al. (2022), Yeap et al. (2020), and Castro-Vargas et al. (2022, 2023)

Species/strain	Source	Approximate coordinates	Year of collection	Generation tested
*B. tryoni* New	Canberra	35.27°S; 149.11°E	2019	G7
*B. tryoni* Old	Sydney	33.90°S; 151.14°E	2006	G128
*B. neohumeralis* New	Cairns	16.89°S; 145.74°E	2019	G6
*B. neohumeralis* Old	Cairns	16.89°S; 145.74°E	2014	G58

### Analytical Strategy

The approach taken here was based on the SPME-MS methodology that has recently been used to characterize *B. tryoni* rectal glands volatiles, where it has enabled significant numbers of compounds, albeit still a minority of the total, to be identified by reference to authentic standards, Kovats indices (KIs) and the National Institute of Standards and Technology (NIST) mass spectral library (Castro-Vargas et al. 2022, 2023). SPME-MS has also been used against bactroceran whole fly emissions (Noushini et al. 2020b, c) but dissected rectal glands were preferred for the current study, even though the whole fly emissions would be a closer representation of the pheromones. This was because of the particular interest in the male volatiles and evidence from preliminary trials for high levels of variation between males in their levels of calling, which suggested standardization of samples for quantitative analyses would be logistically problematic. As detailed below, the harvesting of rectal glands was carefully calibrated against the peak calling time of each species to make it as relevant to pheromone emissions as possible, and the quantitation of their volatiles was based on a statistical normalization of individual peak areas against the total area of all peaks scored in the respective samples. This statistical approach has proven useful in GC-flame ionization detection analysis of *B. tryoni* rectal glands but GC-FID was not used here because, without MS, it was problematic for peak identification.

### Sample Preparation

Sample preparation followed the methods of Castro-Vargas et al. (2023). Briefly, 10 rectal glands from sexually mature males and females (15–20 days after emergence) were excised under a stereoscopic microscope (Leica Microsystems, Heidelberg, Germany), pooled and placed in 10-ml screw-cap SPME headspace vials (Sigma-Aldrich, St. Louis, MO, USA). Samples for each species were collected 3 h before peak calling time, that is, at dawn + 9 h for *B. tryoni* and dawn + 0 h for *B. neohumeralis*. Under the culture conditions used, calling time for *B. tryoni* is quite narrow, with the great majority of mating occurring about 1 h from the onset of dusk, whereas for *B. neohumeralis* calling peaks around midday, but some calling occurs from about dawn + 4 h to dawn + 9 h (Yeap et al. 2020). Male calling is a major determinant of the mating time profile of each species but female receptivity is also important, particularly for *B. tryoni* (Yeap et al. 2020). The collection times used here were chosen with the intention of harvesting the emissions of rectal glands that were primed for mating activity while minimizing volatile releases prior to that point. Vials were then stored at −80 °C until GC–MS analysis. An average of 8 biological replicates for each strain, sex and mating history (virgin vs from mixed-sex cages) combination were analyzed by SPME GC–MS.

### SPME GC–MS Data Acquisition

SPME GC–MS analyses were performed on a Shimadzu TQ8050 gas chromatograph-mass spectrometer (Shimadzu Corporation, Kyoto, Japan) fitted with a fused silica DB5 column (30 m × 0.250 mm inner diameter × 1 µm film thickness; Agilent Technologies, CA, USA) as per Castro-Vargas et al. (2023). Each sample was preincubated for 5 min at 30 °C before adsorption into a conditioned polydimethylsiloxane SPME fiber (30 µm thickness; Restek Corporation, PA, USA). Adsorption was performed for 30 min at 50 °C with constant agitation at 750 rpm. Samples were then desorbed at 250 °C for 60 s in the injector fitted with a Topaz inlet liner (Restek Corporation) in a splitless injection mode. Helium was used as carrier gas at a total flow rate of 1 ml/min. Separation involved an initial 40 °C oven temperature held for 1 min, increased to 100 °C at 10 °C/min with 3 min hold, and then to 325 °C at a rate of 30 °C/min with a 10 min final hold. The MS ion source was held at 230 °C and the mass range was set to detect chemicals with *m*/*z* values from 20 to 600 with a scan interval of 0.3 s. Empty vials with no rectal glands were used as controls after every 10 sample injections and at the beginning and end of the analysis sequence.

### Data Analysis

All chromatograms were analyzed using ACD/Spectrus Processor software version 2019.2.0 (Advanced Chemistry Development, Toronto, Canada). Control chromatograms were subtracted from sample chromatograms to remove peaks that did not originate from the rectal glands. Retained peaks were manually aligned to correct retention time drift. As detailed in Castro-Vargas et al. (2022, 2023), most peaks were identified by matching their spectra against the NIST database (MS search version 2.3) and, where possible, also against authentic standards. In cases where standards were not available, manual analyses of spectra were accompanied by matching KIs against the literature (Noushini et al. 2020a, Park et al. 2020, El-Sayed 2021, Castro-Vargas et al. 2022).

The ACD/Spectrus Processor software version 2019.2.0 was also used to calculate peak areas. Quantitative analysis was then performed on each peak that was present in at least 50% of the samples in at least 1 of the 4 sex/mating history categories in at least 1 species. Statistical analyses for peaks that met these criteria were performed using R Studio (version 1.3.1093). Quantitation of absolute amounts of compounds in SPME samples can be problematic (Nolvachai et al. 2023), so quantitative analyses of each peak were performed on the area of that peak expressed as a percentage of the total areas of all peaks in the sample. The Best Normalize package (Peterson 2021) was used to identify transformations, almost always OrderNorm (Peterson and Cavanaugh 2020), that met the assumptions of normality and heteroscedasticity required in the following analyses.

Principal component analyses (PCAs) were used to assess overall differences between species/sex/mating history categories. Tests for differences in individual peaks between species, strains within species, and their mating history in each sex were carried out using linear modeling (lm function, tidyverse package, version 1.3.0; Wickham et al. 2019). Probability values were adjusted using the Bonferroni correction (p.adjust function, tidyverse package; Wickham et al. 2019). Estimated marginal means (emmeans package; Lenth et al. 2017) were calculated for the peaks that showed significant differences between species, strains, or mating histories, as appropriate. Prior to emmeans analysis, a reduced model (i.e., dropping terms that were not statistically significant) was refitted for some peaks where the higher order interactions involving those terms were also not statistically significant. Emmeans analysis was then undertaken based on the highest order variable(s) that was statistically significant.

Qualitative results for some peaks in the Old *B. tryoni* strain had previously been reported by Castro-Vargas et al. (2023) but a full quantitative analysis of all the consistently detectable peaks is presented for the first time here.

## Results

### Qualitative Analyses of the Volatile Profiles

SPME GC–MS analysis detected a total of 120 peaks across the 2 species. A full breakdown of each peak’s distribution across the various species/sex/strain/mating history categories is given in Supplementary Table S1, with the spectra for each peak in Supplementary Fig. S1 and its peak areas in each sample in Supplementary Dataset S1. The majority of the peaks, 93, were common to both species, with just 5 only found in *B. tryoni* and 22 only found in *B. neohumeralis* (Table 2). The results for *B. tryoni* are broadly similar to a previous SPME GC–MS headspace analysis of that species’ rectal glands (Castro-Vargas et al. 2022), in which 109 peaks were detected.

**Table 2.** Numbers of peaks detected in each species and their respective KIs. Panel A) shows the breakdown by species and sex and panel B) the breakdown by species and strain. A complete breakdown by species, strain, sex, and mating history is given in Supplementary Table S1 and the raw peak area data for all samples are given in Supplementary Dataset S1. Individual peaks that were categorized as major, intermediate, or minor in their relative abundances are bolded, italicized, or neither, respectively. *try* = *B. tryoni*; *neo* = *B. neohumeralis*

Groups	*N*	KI list
**(A) Broken down by species and sex**		
Both species, both sexes	33	674, 786, 789, 867, *883*, *939*, 972, 981, 1019, **1026**, 1028, *1132*, **1142**, **1159**, *1175*, **1201**, **1212**, *1216*, *1219*, *1235*, **1239**, 1244, 1253, 1267, 1296, **1357**, *1442*, 1454, *1464*, *1484*, *1583*, **1760**, **1783**
Both species, but only males	19	702, *738*, **752**, *843*, *902*, 933, 955, 992, 1115, *1164*, 1173, 1179, *1182*, 1187, *1188*, *1189*, *1256*, 1303, 1468
Both species, but only females	36	1059, 1064, *1072*, **1174**, *1183*, 1184, 1185, 1285, 1292, 1306, 1334, *1340*, 1370, 1375, 1388, 1434, 1446, 1473, 1492, 1499, **1525**, **1592**, *1635*, *1659*, *1675*, *1716*, **1723**, **1788**, *1847*, *1868*, 1889, *1911*, *1928*, **1981**, **1989**, *2188*
Both species but not male *try*	2	1083, 1461
Both species but not female *try*	2	*1094*, **1127**
Both species but not male *neo*	1	695
Only *try* males	3	*709*, 1123, 1171
Only *try* females	2	1157, 1609
Only *neo* (both sexes)	1	734
Only *neo* males	17	723, 795, 799, *831*, 951, 962, 966, 989, 993, 1001, 1006, 1087, 1098, 1108, 1124, 1178, 1194
Only *neo* females	4	1172, 1191, 1327, **1551**
**(B) Broken down by species and strain**		
Both species, all strains	79	674, 695, 786, 789, 867, *883*, *939*, 972, 981, 1019, **1026**, 1028, 1059, 1064, *1072*, 1083, *1094*, **1127**, *1132*, **1142**, **1159**, *1164*, 1173, **1174**, *1175*, 1179, *1182*, *1183*, 1187, *1188*, **1201**, **1212**, *1216*, *1219*, *1235*, **1239**, 1244, 1253, *1256*, 1267, 1285, 1292, 1296, 1306, 1334, *1340*, **1357**, 1370, 1375, 1388, 1434, *1442*, 1446, 1454, 1461, *1464*, 1468, *1484*, 1492, 1499, **1525**, *1583*, **1592**, *1635*, *1659*, *1675*, *1716*, **1723**, **1760**, **1783**, **1788**, *1847*, *1868*, 1889, *1911*, *1928*, **1981**, **1989**, *2188*
Both species, but only New strains	1	*738*
Both species, but only Old strains	1	1184
Both species but only *neo* New and *try* Old	1	702
Both species but not *try* New	6	**752**, *843*, *902*, 933, 955, 992
Both species but not *neo* Old	5	1115, 1185, *1189*, 1303, 1473
Only *try* (both strains)	2	1123, 1171
Only *try* New	1	1609
Only *try* Old	2	*709*, 1157
Only *neo* (both strains)	2	1178, 1191
Only *neo* New	16	723, 795, 799, *831*, 951, 962, 966, 989, 993, 1001, 1006, 1087, 1098, 1108, 1124, 1194
Only *neo* Old	4	734, 1172, 1327, **1551**

A majority, 88, of the peaks showed the same pattern across sexes in the 2 species, with 33 found in both sexes, 19 specific to males, and 36 specific to females (Table 2). Five other peaks were found in both species but were not detected in one of the sexes in one of the species. All 5 *B. tryoni*-specific peaks were also sex-specific, 3 in males and 2 in females, and only 1 of the 22 *B. neohumeralis*-specific peaks was found in both sexes, 17 being specific to males and 4 specific to females.

A total of 37 peaks showed presence/absence differences between the New and Old strains of one and/or other species (Table 2). Fewer showed presence/absence differences between strains in *B. tryoni* than in *B. neohumeralis* (12 and 28 peaks, respectively, with 3 peaks showing strain variation in both species). More of these peaks were absent in the Old than New strains of both species (8/12 and 23/28, respectively).

Thirty-five peaks generated a total of 47 cases of presence/absence differences between virgin and mixed (i.e., taken from mixed-sex cohorts) samples of the respective sex (Supplementary Table S1). Similar numbers of cases were seen in the different species, strains, and sexes. However, the direction of the difference did vary between the sexes. In females, the numbers of cases where the peaks were present in virgins but absent in mixed samples were similar to those where they were absent in virgins but present in mixed samples. However, in males, there were fewer cases of presence in virgin/absence in mixed samples than absence in virgin/presence in mixed samples (4 vs 21; χ^2^ = 11.56, *df* = 1, *P* < 0.01), which was largely explained by differences in the New strains of the 2 species (1 vs 14; χ^2^ = 11.27, *df* = 1, *P* < 0.01).

Following Castro-Vargas et al. (2022, 2023), peaks were classified as major, intermediate, or minor according to their relative abundance (≥ 1%, 0.1–0.99% and < 0.1% of total peak area, respectively) in the species/sex/mating history category in which they were most abundant (Table 2, Supplementary Table S1). So defined, 19, 34, and 67 of the peaks were rated as major, intermediate, or minor, respectively. Most of the peaks that were restricted to a single strain and sex were only minor in abundance; for example, 15 of the 16 peaks that were specific to New strain *B. neohumeralis* males were classified as Minor.

### Identified Peaks

In total, 50 of the 120 compounds were identified (Table 3, Supplementary Fig. S1). Forty-seven of the 50 had been identified in *B. tryoni* rectal glands previously, including in most cases against authentic standards (Bellas and Fletcher 1979, Lewis 1988, Fletcher and Kitching 1995, Booth et al. 2006, Kumaran et al. 2014, Pérez et al. 2018, El-Sayed et al. 2019, Noushini et al. 2020a, Castro-Vargas et al. 2022). Six of those (the 6 major amides) had also been previously identified in *B. neohumeralis* rectal glands (Bellas and Fletcher 1979, Fletcher and Kitching 1995). Two of the 3 compounds newly identified in *B. tryoni* rectal glands here have been implicated in sex pheromone functions in other insect species, albeit not yet in Diptera. Acetoin is a female sex pheromone in the summer chafer, *Amphimallon solstitiale*, and a male sex pheromone in the speckled cockroach, *Nauphoeta cinerea* (Sreng 1990, Tolasch et al. 2003), and 2-methyl butanoic acid is a precursor of sex pheromones in the Madeira mealybug, *Phenacoccus madeirensis*, and the palm weevil, *Rhynchophorus ferrugineus* (Zou and Millar 2015, Selim et al. 2017). The third newly identified compound in *B. tryoni* rectal glands, 2-ethyl-4-methyl-1-pentanol, has not previously been implicated in sex pheromone functions in insects.

**Table 3.** Volatiles identified. Previous studies that also identified these compounds in one or other of the 2 species, usually *B. tryoni*, are listed in the footnote

Compound		ID^a^	KI obs	KI literature	Source
1.	Acetoin (Acet)	Here	695	693	♂♀
2.	Ethyl propanoate (EP)	Previous	702	710	♂
3.	3-Methyl-1-butanol (3M1B)	Previous	723	724	♂
4.	Ethyl 2-methylpropanoate (E2MP)	Previous	752	756	♂
5.	(*D*,*L*)-2,3-Butanediol (23But)	Previous	786	788	♂
6.	(*meso*)-2,3-Butanediol (23But(meso))	Previous	789	788	♂♀
7.	2-Methyl butanoic acid (2MB)	Here	831	830	♂
8.	Ethyl 2-methylbutanoate (E2MB)	Previous	843	849	♂
9.	*n*-Propyl 2-methylpropanoate (P2MP)	Previous	848	852	♂
10.	4-Heptanone (4Hep)	Previous	867	872	♂♀
11.	2-Methyl-3-hexanol (2M3H)	Previous	883	858	♂♀
12.	*x*-Octenal isomer1 (Oct-01)	Previous	902	NA	♂
13.	Ethyl 2-methylpentanoate (E2MPen)	Previous	933	941	♂
14.	*x*-Octenal isomer 2 (Oct-02)	Previous	939	NA	♂♀
15.	*n*-Butylcyclopentane (NBCP)	Previous	955	949	♂
16.	Phenol (Phe)	Previous	972	971	♂♀
17.	2-Ethyl-4-methyl-1-pentanol (2E4MP)	Here	981	962	♂♀
18.	*n*-Octen-1-ol (Oct1ol)	Previous	1019	NA	♂♀
19.	2-Ethyl-1-hexanol (2E1H)	Previous	1026	1030	♂♀
20.	N-(2-Methylpropyl)propanamide (MPP)	Previous	1094	1091	♂♀
21.	*N*-(2-Methylbutyl)acetamide (Am1)	Previous	1132	1131	♂♀
22.	*N*-(3-Methylbutyl)acetamide (Am2)	Previous	1142	1150	♂♀
23.	2-Methyl-1,6-dioxaspiro[4.5]decane (2M16DD)	Previous	1157	NA	♀
24.	(*E*,*E*)-2,8-Dimethyl-1,7-dioxaspiro[5.5]undecane (28DDU)	Previous	1159	1148	♂♀
25.	2-Bornanone (2Bor)	Previous	1171	1171	♂
26.	2,7-Dimethyl-1,6-dioxaspiro[4.5]decane isomer 1 (27DDD)	Previous	1172	NA	♀
27.	2-Ethyl-7-methyl-1,6-dioxaspiro[4.5]decane isomer 1 (2E7MDD)	Previous	1174	1157	♀
28.	Borneol isomer 1 (Bor1)	Previous	1179	NA	♂
29.	Diethyl succinate (DS)	Previous	1182	1182	♂
30.	Borneol isomer 2 (Bor2)	Previous	1187	NA	♂
31.	*N*-(2-Methylbutyl)propanamide (Am3)	Previous	1201	1198	♂♀
32.	*N*-(3-Methylbutyl)propanamide (Am4)	Previous	1212	1204	♂♀
33.	*N*-(2-Methylbutyl)-2-methylpropanamide (Am5)	Previous	1235	1226	♂♀
34.	*N*-(3-Methylbutyl)-2-methylpropanamide (Am6)	Previous	1239	1230	♂♀
35.	2-Ethyl-8-methyl-1,7-dioxaspiro[5.5]undecane (2E8MDD)	Previous	1244	1233	♂♀
36.	Methyl dodecanoate (MD)	Previous	1525	1526	♀
37.	Ethyl (*Z*)-9-dodecenoate (E9D)	Previous	1583	1595	♂♀
38.	Ethyl dodecanoate (ED)	Previous	1592	1596	♀
39.	*n*-Propyl dodecanoate (PD)	Previous	1675	1685	♀
40.	Methyl (Z)-9-tetradecenoate (M9T)	Previous	1716	1703	♀
41.	Methyl tetradecanoate (MT)	Previous	1723	1725	♀
42.	Ethyl (*E*)-9-tetradecenoate (E9T)	Previous	1783	1769	♂♀
43.	Ethyl tetradecanoate (ET)	Previous	1788	1795	♀
44.	Ethyl 12-methyltetradecanoate (E12MT)	Previous	1868	1861	♀
45.	*n*-Propyl tetradecanoate (PT)	Previous	1889	1887	♀
46.	Methyl (*Z*)-9-hexadecenoate (M9Hex)	Previous	1911	1911	♀
47.	Methyl hexadecanoate (MHex)	Previous	1928	1927	♀
48.	Ethyl (*Z*)-9-hexadecenoate (E9Hex)	Previous	1981	1975	♀
49.	Ethyl hexadecanoate (EHex)	Previous	1989	1990	♀
50.	Ethyl (*E*)-9-octadecenoate (E9Oct)	Previous	2188	2174	♀

^a^ Bellas and Fletcher (1979), Lewis (1988), Fletcher and Kitching (1995), Booth et al. (2006), Kumaran et al. (2014), Pérez et al. (2018), El-Sayed et al. (2019), Noushini et al. (2020a), Park et al. (2020b), and Castro-Vargas et al. (2022, 2023).

The 50 identified compounds included: 5 short-chain esters, 8 alcohols, 1 ketone, 2 aldehydes, 1 acid, and 1 alkane and acetoin in a relatively short KI range (≤ 1059); 7 aliphatic amides, 5 spiroacetals, 3 terpene derivatives, and a diester in a mid KI range (1064–1327); and 15 fatty acid esters in a long KI range (≥ 1334). These 3 KI ranges closely match the retention time/KI ranges in Castro-Vargas et al. (2022, 2023), except that the larger number of compounds identified here enabled refinement of the boundaries of the mid KI region slightly, so it contained all the amides and spiroacetals now identified. In total, 34 of the 120 rectal gland peaks detected lay in the short KI range, 48 in the mid KI range, and 38 in the long KI range.

### Quantitative Analysis of Total Peak Areas

**Fig. 1.**
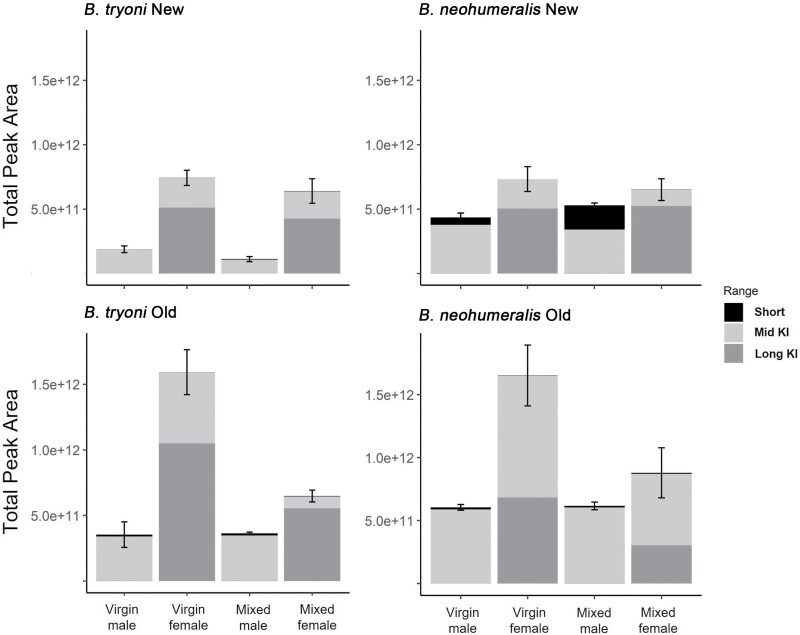
Means and standard errors of total peak areas for rectal gland volatiles in each species, strain, sex, and mating history category. The totals for each of the short, mid, and long KI ranges are also indicated.

Pairwise comparisons showed that total peak areas (i.e., the sum of the areas of all detected peaks) were always higher in females than males of the same species/strain/mating history category (~1.5- to 6-fold in *B. tryoni*, ~1.2- to 3-fold in *B. neohumeralis*) but differences due to the other factors were more complex (Fig. 1). Mating history had little effect on total peak area in males or New strain females of either species but in Old strain females of both species total peak area was only about half as much in mixed as virgin females. In females, the total peak area was similar for the 2 species, but in males the total peak area in *B. neohumeralis* was 2- to 3-fold higher than the corresponding categories in *B. tryoni.* Domestication had little effect on mixed females of either species but was associated with ~ 2-fold increase in virgin females of both species and a ~ 1.5- to 3-fold increase in males in all 4 species/mating history categories.

The contributions of peaks from different KI ranges to the totals also differed substantially between the sexes (Fig. 1). Peaks in the short KI range only comprised more than 5% of the total in New *B. neohumeralis* males, where they contributed 13% and 33% in virgin and mixed flies, respectively. Peaks in the mid KI range contributed the great majority (65–95%) of total peak areas in all male categories but lesser proportions (14–63%) of total peak areas in females. Peaks in the long KI range contributed less than 5% to the totals in all males, 34–39% in virgin and mixed Old *B. neohumeralis* females, and 65–83% in all other female categories.

### Principal Component Analyses

**Fig. 2.**
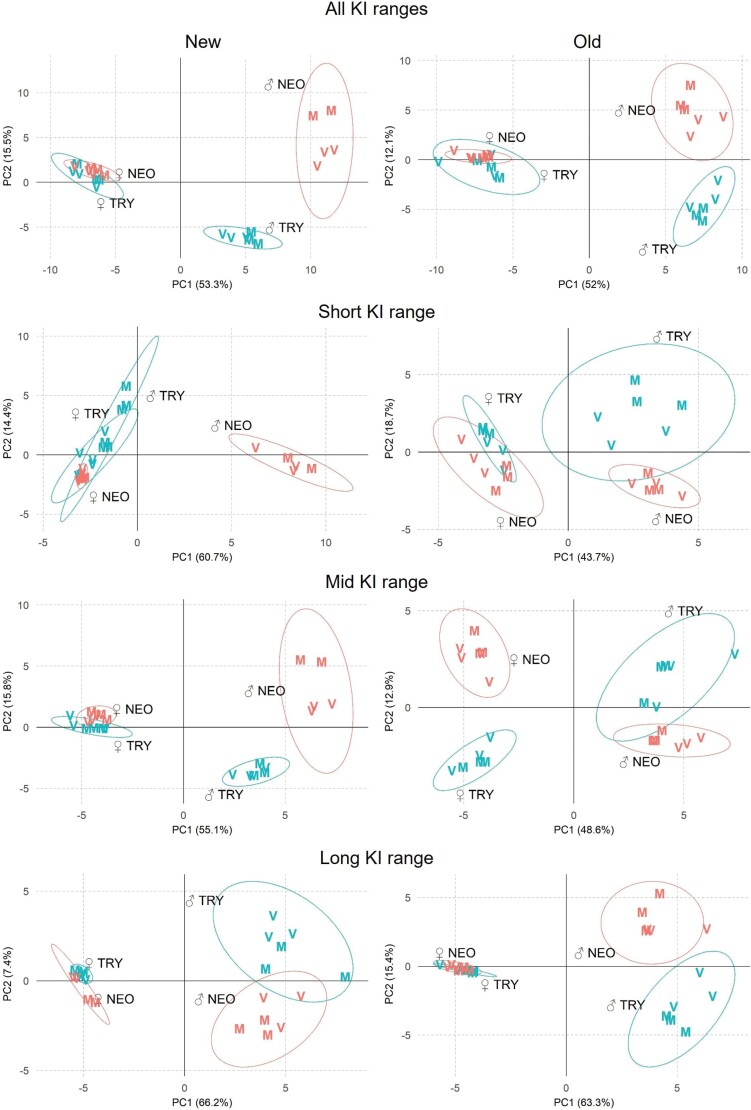
PCAs of percentage peak areas of rectal gland peaks in New and Old strains of each species. The top 2 panels give the overall results using all 120 peaks and the panels below them give the results for the peaks in the short, mid, and long KI ranges, respectively. Virgin and mixed samples are represented by V and M, respectively.

As a first step in elucidating compositional differences in the rectal gland volatiles, PCAs were carried out on the data for each peak expressed as a percentage of the total area of all peaks in each sample. Separate analyses were conducted for the New and Old strains and in both cases PC1 and PC2 combined were found to account for over half of the total variance (Fig. 2). The results for both pairs of strains showed clear differences between the sexes and, in males but not females, also between species. There was little difference between virgin and mixed flies in most of the 4 species × sex combinations, the exceptions being some relatively small but consistent differences in New *B. neohumeralis* males.

Equivalent PCAs carried out on the data for peaks in each of the short, mid, and long KI ranges showed broadly similar patterns, but with a few notable exceptions (Fig. 2). The sexes were again well separated, except in the short KI PCA for the New *B. tryoni* strain, and the distributions were tighter in females than males for all but the short and mid KI plots for the Old strains of both species. Species were again well separated in all male plots except that for the long KI plot for the New strains. Conversely, the species were again not clearly distinguished in any of the female plots, except for the mid KI plot for the Old strains. Thus, notwithstanding the generally clear separation between the sexes and between the species in males, there were several differences due to domestication in particular sex and KI range combinations.

Note that the PC analyses normalized the data for each peak to zero mean and unit variance, so that the data for all peaks were given equal weight in the analysis regardless of their overall abundance relative to one another. They thus tested for shared patterns of difference between categories of flies among the peaks but, without consideration of abundance differences between peaks, were insufficient alone to account for the KI range-specific patterns of difference between categories of flies in total peak areas in Fig. 1.

### Quantitative Analyses of Individual Peaks

Complementing the PCAs, linear modeling was therefore carried out to identify specific peaks that differed in abundance between the species in each sex. The modeling tested for effects of species, domestication (i.e., New vs Old strains), mating history, and all the interactions between these 3 factors. Significant effects were found for 89 individual peaks, that is, 74% of all those tested (Supplementary Tables S2 and S3). A higher proportion of the peaks scored in males showed significant effects than did those in females: 76% (59/78) and 58% (47/81), respectively (χ^2^ = 4.78, *df* = 1, *P* < 0.05), with 16 peaks significant in both datasets. Similar proportions of the peaks in each of the 3 KI ranges varied significantly in both males and females (χ^2^ = 4.51 and 2.05, respectively, *df* = 2, *P* > 0.05 in both cases). Similar proportions of the peaks in each of the 3 abundance categories also varied significantly in males (χ^2^ = 3.23, *df* = 2, *P* > 0.05) but proportionally more major than intermediate or minor peaks varied significantly in females (94% vs 42% and 51%, respectively; χ^2^ = 13.18, *df* = 2, *P* < 0.001).

Forty of the significantly varying peaks had been identified. Five of the 6 major amides varied significantly in both sexes and amide 1 varied significantly in females only. Most of the other significantly varying peaks identified in males were in the short KI range. Nine of the other significantly varying peaks identified in females were fatty acid esters in the long KI range and 3 were spiroacetals in the mid KI range.

The models required for most of the significantly varying peaks had main or interaction terms involving species and domestication (45 and 50, respectively, in males and 35 and 38, respectively, in females), with only a minority involving mating history (19 in males and 9 in females) (Supplementary Tables S2 and S3). One or more interactions, most commonly species × domestication, were retained in most of the models. However, models for several peaks involved a main effect of a particular factor that was not involved in significant interactions: 12, 20, and 5 for species, domestication, and mating history, respectively, in males, and 9, 9, and 5, respectively, in females (Table 4). Identified compounds among these in males included 9 cases involving short KI compounds, mainly alcohols, and 9 involving mid KI compounds, mainly amides, but no long KI compounds. Those in females included 5 cases involving short KI compounds, again mainly alcohols, 4 involving mid KI compounds, again mainly amides but also including a spiroacetal, and 5 involving long KI compounds, all fatty acid esters.

**Table 4.** Peaks showing significant main effects of species (Sp), domestication (Dm), and mating history (Mh) on percentage peak areas without significant relevant interactions. As per Table 2, peaks rated major, intermediate, or minor in relative abundance are marked in bold, italicized, or neither, respectively. See also Supplementary Table S2 for a full breakdown of all the peaks showing significant main or interaction effects

Main effect	Males					Females				
	Category in excess	n	Short KI	Mid KI	Long KI	Category in excess	n	Short KI	Mid KI	Long KI
Sp	*try*	5	674	Bor2 1171, **Am4**, **Am6**	*1464*	*try*	4			1473, *PD*, **1760**, *E9Oct*
	*neo*	7	*2M3H*, 992, 1019	*1164*, 1173, 1253	1468	*neo*	5	1059	**1127**, **Am2**, 1191	1492
Dm	New	11	23But (meso), **2E1H**, 1028	1115, Bor1, Bor2, *1188*, *1256*	*1442*, 1454, *1484*	New				
	Old	9	4Hep, *2M3H*, E2MPen, 992	*MPP*, *Am5*, **Am6**	1468, **1760**	Old	9	Oct1ol, **2E1H**	**2E7MDD**, **Am4**, *Am5*, 1253, 1267, 1306	**MD**
Mh	Virgin male	2	*1164*	**Am4**		Virgin female	2			**EHex**, *E9Oct*
	Mixed male	3	4Hep, *Oct-02*,	**Am2**		Mixed female	3	*Oct-02*, Oct1ol, **2E1H**		

While the modeling had exposed many significant effects, there were 7 peaks in males and 10 in females for which particularly large effects were found (Table 5 and see also Supplementary Tables S3 and S4 for full lists of all the significant differences). Large effects were defined as ones in which at least 1 category of sample implicated in a significant term comprised > 1% of total peak area and the difference between the highest and lowest categories in that term was either >1% or >10-fold. The peaks involved in the large differences were all short and mid KI compounds in males and mid and long KI compounds in females. They included amides 2, 4, and 6 in both sexes, plus amide 3, a short KI ester and a short KI alcohol in males and a spiroacetal and 5 fatty acid esters in females. The peaks involved in the large differences together accounted for the great majority of the total peak areas in each species × domestication combination in both sexes, albeit somewhat less in New and Old *B. neohumeralis* than the other combinations in males and females, respectively.

**Table 5.** Percentage peak areas for peaks showing large main or interaction effects in males A) and females B). Large effects are defined as those where the value for at least 1 category implicated in the model was > 1% and the differences between the largest and smallest values for the categories were either >1% or >10-fold. 95% confidence limits are also given. Note that the values shown are back-transformed estimated marginal means (emmeans) to ensure that their confidence limits were calculated on data that had been transformed to establish normality and homoscedasticity. See Supplementary Tables S4 and S5 for the emmeans for all the significantly varying peaks for males and females, respectively. Abbreviations are as per earlier tables

(A) Males											
Term	Group	752 E2MP	1026 2E1H	1127		1142 Am2	1201 Am3		1212 Am4		1239 Am6
Sp	*try*	–	–	–		–	–		76.8 (72.4,77.8)		8.7 (8.2,10.6)
	*neo*	–	–	–		–	–		64.7 (63.4,65.5)		3.6 (0.9,7.6)
Dm	New	–	3.9 (0.9,10.9)	–		–	–		–		3.6 (0.9,7.7)
	Old	–	0.3 (0.3,0.7)	–		–	–		–		8.7 (8.1,10.5)
Mh	Virgin ♂	–	–	–		5.6 (5.5,6.1)	–		72.5 (70.3,77.2)		–
	Mixed ♂	–	–	–		7.7 (6.8,10.0)	–		65.3 (64.7,69.2)		–
Sp:Dm	*try* New	0.0 (0.0,0.1)	–	0.0 (0.0,0.0)		6.3 (5.5,7.5)	2.4 (2.2,3.2)		–		–
	*neo* New	0.4 (0.2,0.8)	–	10.3 (8.1,11.6)		4.3 (3.6,5.5)	2.0 (1.5,2.4)		–		–
	*try* Old	2.5 (2.3,3.3)	–	0.0 (0.0,0.0)		5.8 (5.5,7.1)	2.5 (2.3,3.5)		–		–
	*neo* Old	1.2 (0.8,2.3)	–	0.0 (0.0,0.0)		19.9 (13.2,20.3)	4.2 (3.5,5.1)		–		–
**(B) Females**											
**Term**	**Group**	**1142** **Am2**	**1159** **28DDU**	**1212** **Am4**	**1239** **Am6**	**1357**	**1592** **ED**	**1783** **E9T**	**1788** **ET**	**1981** **E9Hex**	**1989** **EHex**
Sp	*try*	0.2 (0.1,0.3)	–	–	–	–	–	–	–	–	–
	*neo*	1.3 (0.7,1.8)	–	–	–	–	–	–	–	–	–
Dm	New	–	–	2.4 (1.9,3.2)	–	–	–	–	–	–	–
	Old	–	–	7.8 (4.4,10.5)	–	–	–	–	–	–	–
Sp:Dm	*try* New	–	25.0 (19.6,30.6)	–	2.1 (1.2,4.3)	0.5 (0.3,0.7)	29.7 (26.0,34.1)	3.2 (2.4,4.5)	23.2 (19.3,25.3)	6.3 (4.6,7.9)	1.9 (1.7,2.2)
	*neo* New	–	19.1 (13.6,24.0)	–	1.2 (0.7,2.1)	0.3 (0.2,0.5)	28.6 (25.2,31.9)	7.8 (5.3,11.2)	25.2 (23.2,28.3)	9.0 (7.8,9.3)	1.7 (1.5,1.9)
	*try* Old	–	15.7 (8.6,19.3)	–	1.7 (1.1,2.8)	0.3 (0.1,0.4)	32.0 (28.6,40.2)	5.3 (4.1,9.3)	23.0 (17.3,24.6)	8.2 (6.6,9.1)	2.0 (1.8,2.3)
	*neo* Old	–	36.4 (31.9,38.6)	–	9.3 (5.8,11.8)	1.4 (1.0,1.8)	14.9 (6.2,22.8)	2.3 (1.6,3.2)	12.1 (9.5,13.7)	3.3 (1.9,4.9)	0.5 (0.3,0.8)

The 3 largest differences in males involved amide 4, which was over 10% higher in *B. tryoni* than *B. neohumeralis* in both the New and Old strains, and amide 2 and peak 1127, which were over 10% higher in Old and New *B. neohumeralis*, respectively, than in other species × domestication combinations (Table 5). The other large differences due to species and domestication in males involved differences of ~1–4% favoring *B. tryoni* and Old strains (amide 6), New strains of both species (2-ethyl-1-hexanol), Old *B. tryoni* (ethyl-2-methylpropanoate), and Old *B. neohumeralis* (amide 3). There were also differences of similar magnitudes among males due to mating history differences in amides 2 and 4. Although the differences were smaller, most of the other significantly varying peaks in males also favored higher percentage peak areas in New *B. neohumeralis* than in other male categories (Supplementary Table S4). This largely reflected the fact that the large differences in males accounted for less of total peak areas for this strain than for the other 3.

The 3 largest differences in females all involved Old *B. neohumeralis*, which was over 10% higher in the spiroacetal (*E*,*E*)-2,8-dimethyl-1,7-dioxaspiro[5.5]undecane, and over 10% lower in the fatty acid esters ethyl dodecanoate and ethyl tetradecanoate, than it was in all the other species × domestication combinations (Table 5). The other large differences in females, ranging from ~1% to 8%, involved higher values for *B. neohumeralis* in both strains (amide 2), Old strains of both species (amide 4) and Old *B. neohumeralis* (amide 6 and peak 1357), and lower values for Old *B. neohumeralis* (ethyl (*E*)-9-tetradecenoate, ethyl (*Z*)-9-hexadecenoate, and ethyl hexadecanoate). Thus, all 5 identified fatty acid esters showing large differences in females involved relatively low values for Old *B. neohumeralis.*

Old *B. neohumeralis* was also the clear outlier for several other peaks showing smaller differences among the 4 species × domestication combinations in females (Supplementary Table S4). However, in contrast to the situation for the 5 fatty acid esters showing large differences in females above, most of the smaller differences in females in the mid or early long KI ranges (i.e., 15 of 17 peaks from 1094 to 1499 with significant species × domestication interactions) involved relatively high values in Old *B. neohumeralis*. Furthermore, several of the small differences that were shared by the Old strains of both species in this KI 1094–1499 interval also had relatively high values. Many of the peaks in question could not be identified but 2 that were identified were spiroacetals.

## Discussion

Differences between tephritid species in male rectal gland emissions have been reported previously (Symonds et al. 2009, Castro-Vargas et al. 2022), but most of the previous studies have focused on a relatively small number of compounds and relatively wide phylogenetic coverage. By comparison, the present study finds several qualitative and many quantitative differences in a wide range of volatiles between 2 sibling *Bactrocera* species, with many of the differences being sex-specific. Given that male rectal gland emissions appear to elicit responses in females of *B. tryoni* (Giannakakis and Fletcher 1978, Kumaran et al. 2014), and in both sexes of some other tephritids (Mazomenos and Pomonis 1983, Mazomenos and Haniotakis 1985, Canale et al. 2013, 2015, Kumaran et al. 2014, Mavraganis et al. 2017), it seems likely that some of the differences described here affect reproductive behavior, and hence, potentially, reproductive isolation, between *B. tryoni* and *B. neohumeralis*. The fact that some differences depended on the mating status of the flies could also reflect a role for the compounds in question in reproductive behavior, although it is unclear how they would contribute to reproductive isolation.

Sex-dependent species differences were evident in both the analyses of total peak areas and the PCAs of their compositions and both analyses showed that the nature of the differences in each sex varied with domestication and KI range. However, the differences exposed differed between the 2 analyses. In particular, the analyses of total peak areas showed New strain *B. neohumeralis* males had disproportionately high total levels of short KI compounds and females of this strain had relatively high levels of short and mid KI compounds, while the PCAs only clearly separated the 2 species into nonoverlapping groups in the short and mid KI ranges in New strain males, the long KI range in Old strain males and the mid KI range in Old strain females.

Most of the individual peaks also showed significant sex-dependent differences among the species/domestication/mating history categories, most again involving effects of species and/or domestication and a smaller number involving mating history. Complicating their interpretation but consistent with the complexities evident in the first 2 analyses, most models involved at least one interaction term, most commonly between species and domestication. However, the models for some compounds involved main effect terms for a particular factor without any confounding interaction terms with other factors. In males, most of these were short and mid KI compounds, including some identified alcohols and amides, respectively, whereas in females, they were a more even mix of the 3 KI ranges, including some of the amides again but also several fatty acid esters.

A second issue complicating interpretation of the results for specific peaks arose because they were based on analyses of the percentage contributions of each peak to overall totals rather than absolute amounts. This could lead to an overestimate of the number of peaks showing differences, as large contributions of particular peaks would have necessarily led to compensatory differences in others. Nevertheless, the overall diversity of models fitted across the different peaks, and the differing magnitudes and directions of the effects they represented, still imply, not only that the composition of rectal gland emissions differed substantially between species and domestication categories but also that large numbers of peaks contributed to those differences. This is consistent with evidence from other tephritids that the male sex pheromone is most often a complex blend of compounds (Jang et al. 1989) and that male rectal gland emissions may also have other functions, for example, in male-male interactions (Ohinata et al. 1977, Nishida et al. 1988, Tan and Nishida 1996, Būda et al. 2020).

Given the issue of compensatory differences, particular attention was paid to peaks showing large differences. Such differences between species and/or domestication categories were found for 7 short or mid KI compounds in males and 10 mid or long KI compounds in females. Amides 2, 4, and 6 were involved in both sexes, albeit not in the same ways in any of them. The other peaks showing large differences included a short-chain ester and alcohol in the males and a spiroacetal and 5 fatty acid esters in females. The relative abundances of some of these compounds suggest that the differences in them may be functionally significant but do not necessarily imply that they have roles as sex pheromones; sex pheromones in other species are often quite unabundant in the overall profile (Brezolin et al. 2018). While male sex pheromone functions have previously been proposed for the amides (Fletcher 1968, Bellas and Fletcher 1979), direct empirical evidence is still lacking and, as noted elsewhere (El-Sayed et al. 2019, Castro-Vargas et al. 2022), the relatively high amounts of the amides in female rectal gland emissions argue against a primary male pheromone function.

The species differences seen in the New strains are clearly more relevant than those in the Old strains to mating behaviors in natural populations. In this light, it is noted that the species differences were more pronounced in New males than New females. This trend held in total rectal gland emissions and both the PCA and individual peak analyses of their compositions, with most of the differences accounted for by short KI or mid KI peaks. The data thus bear out the previously published evidence for sex pheromone functions for male rectal gland emissions in *B. tryoni* and also support the hypothesis that such a function also applies to the *B. neohumeralis* emissions but is mediated by different chemistry.

Another notable precedent study in this respect involved the sympatric sibling species *Bactrocera carambolae* and *B. papayae* (now synonymized with *B. dorsalis*) (Wee and Tan 2005). These 2 species have been shown to differ in 2 specific rectal gland volatiles produced endogenously from the same precursor, methyl eugenol, which the flies ingest from host fruit and is also widely used in artificial lures for the species (Nishida et al. 1988, Tan and Nishida 1996, Wee 2000). The variable penetrance of the volatile phenotypes in hybrids bred in the laboratory suggest a complex mode of inheritance for the differences. Unlike the findings to date for *B. tryoni* and *B. neohumeralis*, morphologically hybrid flies have also been found in the field, and these flies also show a range of phenotypes for the volatiles in question (Wee and Tan 2005).

Unlike the situation in males, there is currently no direct behavioral evidence for the existence of female sex pheromones in either *B. tryoni* or *B. neohumeralis*. However, female sex pheromones have been reported for other tephritids (Baker et al. 1980, Mazomenos and Haniotakis 1981, Canale et al. 2015, Noushini et al. 2020c) and the New strain data provide indirect evidence suggesting they likely exist in *B. tryoni* and *B. neohumeralis* as well, and could again be mediated by somewhat different chemistries in the 2 species. Evidence for this from the current work includes the large number of individual peaks that were female-specific or female-selective, the large number of these that differed between the species, and the strong representation in these groups of fatty acid esters and spiroacetals which have been implicated in pheromonal functions in other species (Mazomenos and Haniotakis 1985, Canale et al. 2015, Noushini et al. 2020b).

Comparisons of Old and New strains of each species showed a mix of shared and species-specific effects of domestication. There were large increases in total emissions in virgin females of the Old strains, despite the loss of some individual peaks in Old strain females of both species, particularly in *B. neohumeralis*. Many individual peaks also showed quantitative differences in abundance between the New and Old strains, some of them shared by the 2 species but most being species-specific. Even some of the relatively few effects of mating history on the emissions profiles depended on domestication status. For example, New, but not Old, strains had more peaks in mixed than virgin males in both species, total emissions were much higher in virgin than mixed females in the Old, but not New, strains of both species, and total short KI emissions were much higher in mixed New male *B. neohumeralis* than they were in any other category.

While not as directly relevant as the New strain results to mating behaviors in natural populations, the Old strain results are nevertheless germane to the control of field populations. This is because the efficacy of SIT programs being developed for *B. tryoni* depends on the mating success of the sterilized mass-release males (Pérez-Staples et al. 2013). SIT stocks have necessarily been through numerous generations of artificial rearing prior to their use in SIT programs and the current results suggest their pheromone emissions could change in that time, potentially compromising their attractiveness as mates in the field. Systematic outcrossing of SIT strains to newly caught material has been advocated to alleviate inbreeding depression in SIT strains (Gilchrist et al. 2012), and it may also help maintain wild-type sex pheromone compositions. While many of the specific pheromonal changes seen here during the domestication of *B. neohumeralis* differed from those in *B. tryoni*, the fact that they occurred suggests the issue could also apply more generally to SIT programs deployed against other tephritids.

Intriguingly, many of the differences found in individual peaks involved *B. neohumeralis* as the major outlier—New strains particularly in males and Old strains particularly in females. Such apparently large changes during the domestication of this species occurred despite the fact that fewer generations separated the 2 *B. neohumeralis* strains than separated those of *B. tryoni* (Table 1). It may be relevant that *B. neohumeralis* is much harder to maintain in laboratory culture than *B. tryoni*, with a much higher proportion of cultures lost within the first few generations. The selective pressures on social interactions would clearly have been very different in the laboratory from those experienced by either species in their respective field environments. However, these pressures may have been greater for *B. neohumeralis* than for *B. tryoni*.

The evidence presented here for many differences in the rectal gland profiles of the recently collected vs domesticated strains of each species also complements a previous finding of variation in the profiles between different populations of *B. tryoni*; 26 of over a hundred peaks tested in that study showed such presumptively genetic variation (Castro-Vargas et al. 2023). Only 8 of the 26 were identified, but all 8 (*n*-propyl 2-methylpropanoate, ethyl 2-methylpentanoate, 2-methyl-3-hexanol, isomers of octenol and octenal, amides 1 and 5, and methyl dodecanoate) also varied in the current study. All 26 of the intra-specific differences in the previous study were only significant in males and it was noted there that the failure to find differences in females may be at least partly due to lower sampling intensity in the female dataset. The results of the present study support that idea. Overall, the results of the 2 studies show that each species has a reservoir of heritable variation and a capacity for rapid evolutionary change in some biochemistries directly linked to reproductive biology, which could form the basis for the development of additional prezygotic isolating mechanisms between populations beyond those already evident between the 2 species.

A wide range of biochemical pathways are likely involved in both the species and domestication differences described herein because the synthesis of short KI alcohols or esters, the amides, the spiroacetals, and the fatty acid esters involve very different biochemical pathways (Fletcher et al. 2002, Schwartz et al. 2005, Booth et al. 2006, 2007, 2009, Singh et al. 2014, Park et al. 2020, Castro-Vargas et al. 2022). Curiously, however, the limited comparative genomic data currently available (Gilchrist and Ling 2006) have thus far failed to find any fixed differences between the 2 species. Nor is anything yet known about genetic changes during domestication in either species which might affect these pathways. Indeed, little is yet known about the genetics of domestication in any insect (Hoffmann and Ross 2018). Notably, however, Raphael et al. (2019) found several tissue- and temporally specific differences in the transcriptome profiles of the 2 species, so regulatory changes may underpin much of the rapid changes in reproductive biochemistry described herein.

Recent work on *B. dorsalis* and *Zeugodacus cucurbitae* also raises the possibility that rectal bacteria might also contribute to some of the variation reported here (Ren et al. 2021, Gao et al. 2023). Those 2 species differ in the relative proportions of the tri- and tetra-methylpyrazine components of their male sex pheromone blends. These compounds are synthesized by rectal bacteria from precursors produced by the hosts. The precursors are the amino acids glycine and threonine, the relative proportions of which can be influenced by both the genes and the diet of the hosts. No pyrazines were found among the 50 identified rectal gland volatiles in the species studied here, but the major aliphatic amides studied here are also produced from amino acid precursors, in this case, leucine and isoleucine, and the relative proportions of the amides in rectal gland emissions of *B. tryoni* can be varied by manipulating the relative amounts of those amino acids in its diet (Castro-Vargas et al. 2022). Further work is now needed to assess the possible effects of rectal bacteria on the emissions of *B. tryoni* and *B. neohumeralis.*

However, a key priority for future research will be to assign behavioral functions to specific volatiles in the profiles described here. An important early step toward this goal will be to establish the extent to which the differences in headspace volatiles of dissected rectal glands, such as have been found here, translate to differences in headspace volatiles of live calling males. As noted, earlier experiments along these lines were beset by practical difficulties trying to manage and standardize the amounts of calling done by different males tested. However, the success of the statistical normalization approach applied here suggests this should now be achievable. Identified peaks showing both strong sex and species differences in the 2 species in the data from live calling males and with evidence for pheromonal functions in other insects could then be prioritized for electroantennagram and behavioral assays (Robacker et al. 1986, Raptopoulos et al. 1995, Canale et al. 2013, van der Pers et al. 2017). While significant numbers of the volatiles of potential interest found herein have yet to be identified, the database of spectra in Supplementary Fig. S1, together with the rapidly expanding NIST database of spectra for known compounds, should reduce this issue over time.

## Supplementary Material

ieae066_suppl_Supplementary_Material
