# Supplementary material for: Differential pheromone profile as a contributor to premating isolation between two sympatric sibling fruit fly species
Source: J Insect Sci. 2024 Jun 24;24(3):26. doi: 10.1093/jisesa/ieae066 (PMC11195474; doi:10.1093/jisesa/ieae066)
Supplement: ieae066_suppl_Supplementary_Material [file ieae066_suppl_supplementary_material.docx]

## Supporting information

**Supplementary Fig. S1. Mass spectra and segments of the respective chromatograms for the peaks recorded in either or both the rectal gland and whole male analyses.**

**Supplementary Table S1. Peaks detected in the two species*’* rectal glands.** The numbers of peaks found in each species, strain, sex and mating history category are shown, together with the KIs of all peaks in each category. Ticked green cells and pink cells indicate the peak was found or not found in the species/strain/sex/mating history category in question respectively. n is the number of strains with the distribution in question. Sum is the number of categories with the distribution in question. spp = species; str = strain; sex = sexes; V = virgin; M = mixed; *try* = *B. tryoni*; *neo* = *B. neohumeralis*; mals = males; fems = females.

**Supplementary Table S2. Peaks showing significant effects on percentage peak areas due to Species (Sp), Domestication (Dm), Mating history (Mh) or any of their interactions.** See also Supplementary Table S3 for the modelling results for peaks for which no significant effects were found.

**Supplementary Table S3. Results of linear modelling to detect significant effects on percentage peak areas of all rectal gland peaks due to species differences, domestication and mating status.** F ratios are given for each term in the model. The sexes were analysed separately, with the results for males in panel A and those for females in panel B. Sp = Species; Dm = Domestication; Mh = Mating history. ^⁎^Bonferroni-corrected P < 0.05, ^⁎⁎^ Bonferroni-corrected P < 0.01, ^⁎⁎⁎^ Bonferroni-corrected P < 0.001.

**Supplementary Table S4. Back-transformed emmeans of percentage peak areas for male rectal gland peaks showing significant main or interaction effects in Supplementary Table S2.** 95% confidence limits are also shown. Abbreviations are as per previous Tables and Figures.

**Supplementary Table S5. Back-transformed emmeans of percentage peak areas for female rectal gland peaks showing significant main or interaction effects in Supplementary Table S2.** 95% confidence limits are also shown. Abbreviations are as per previous Tables and Figures.

**Supplementary Dataset S1. Raw peak area data for all the samples and peaks used in the rectal gland analyses.** Only peaks that were detected in at least 50% of the samples in at least one category were included in the analysis. Note that six peaks (KIs 1226, 1320, 1382, 1399, 1412, 1596) could not be scored in all samples because their signal was obscured by larger, closely adjacent peaks in those samples. They are indicated as N/A in those samples and none of the data for those peaks were included in the analyses. Files are available online at the following link: https://github.com/cas212/Supplementary_Dataset1.

**Supplementary Fig. S1.**

| Compound | Spectrum | Peak |
| --- | --- | --- |
| 674 | 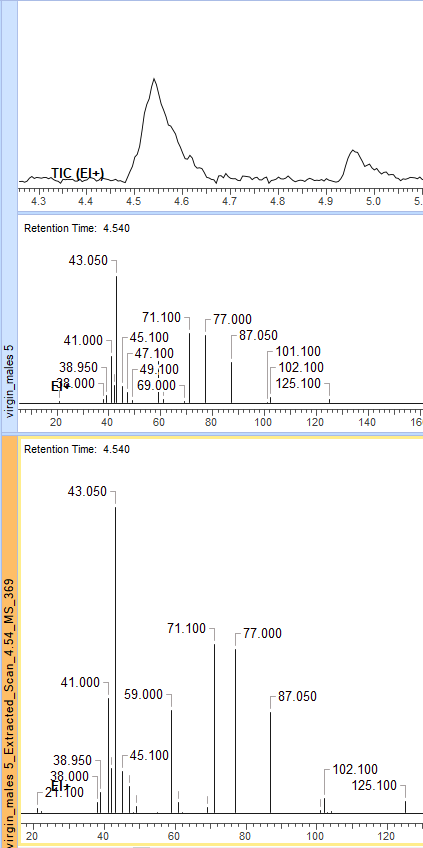 | 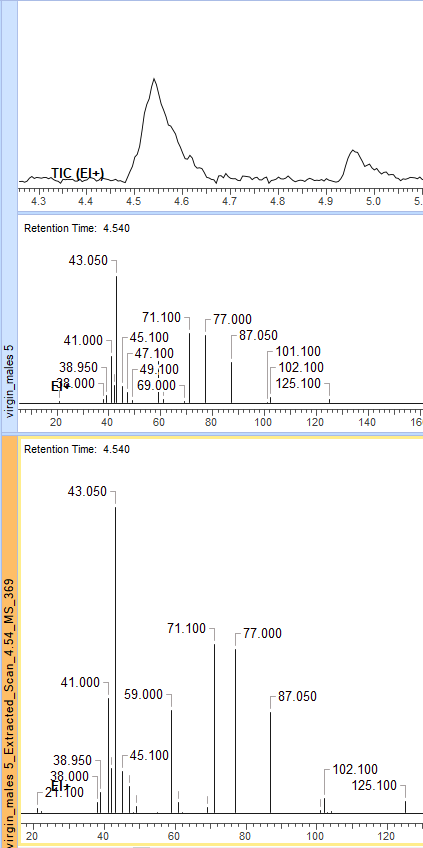 |
| 695  Acetoin | 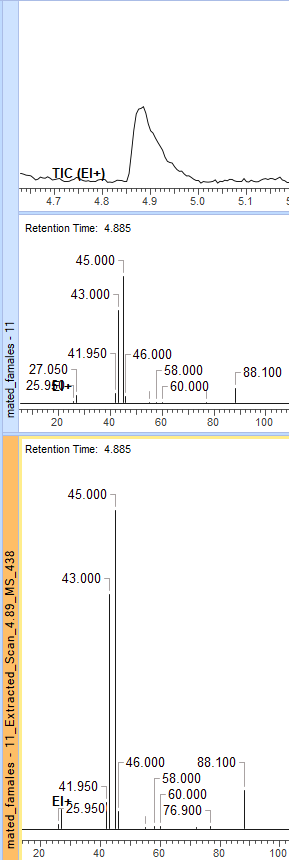 | 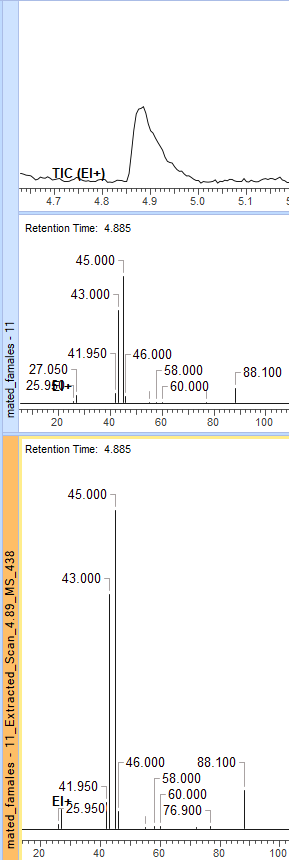 |
| 702  Ethyl propanoate | 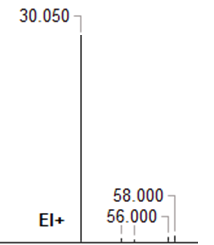 | 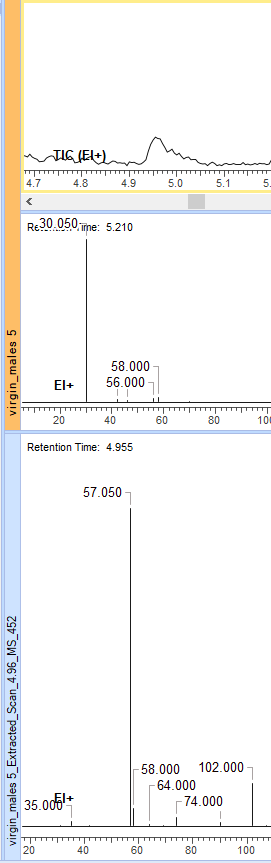 |
| 709 | 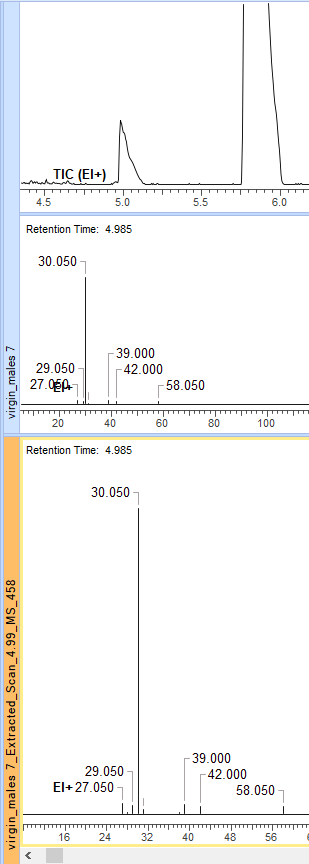 | 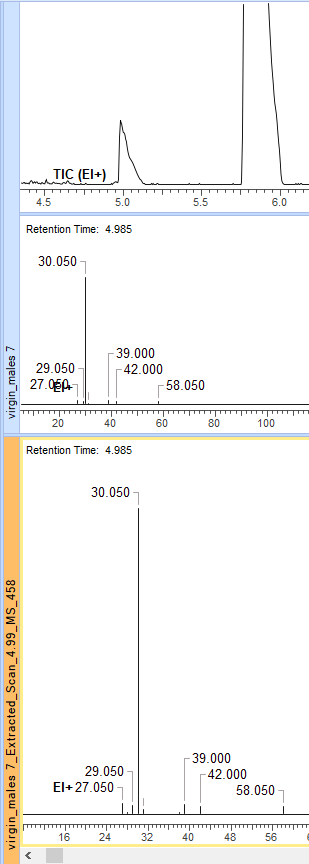 |
| 723  3-Methyl-1-butanol | 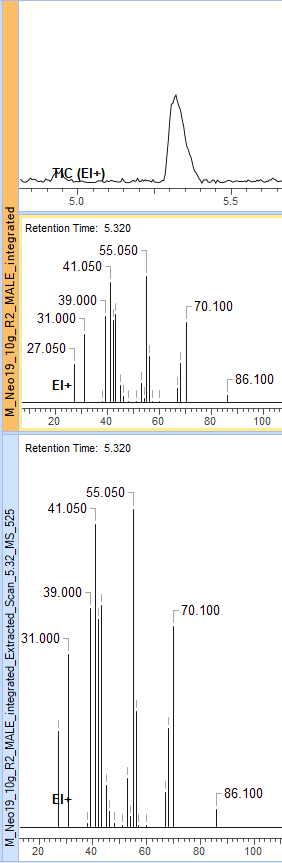 | 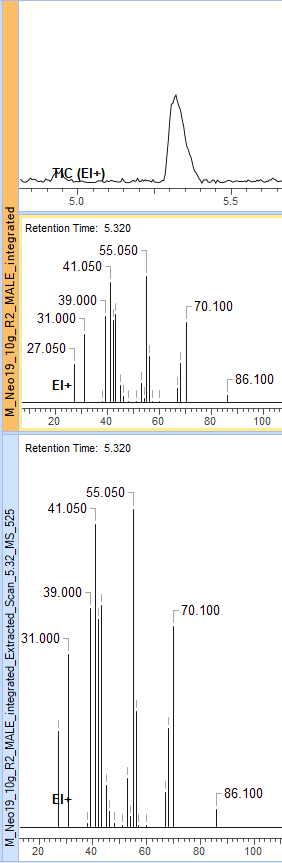 |
| 734 | 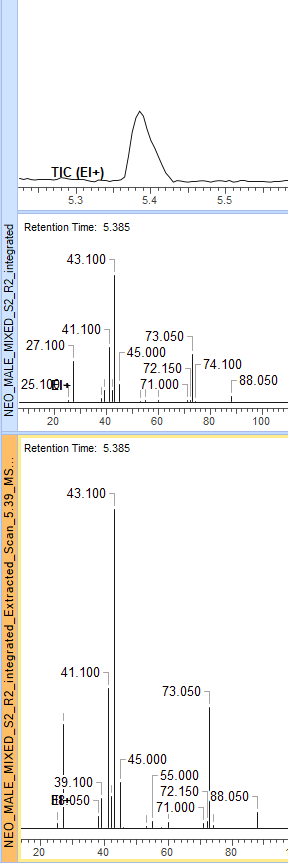 | 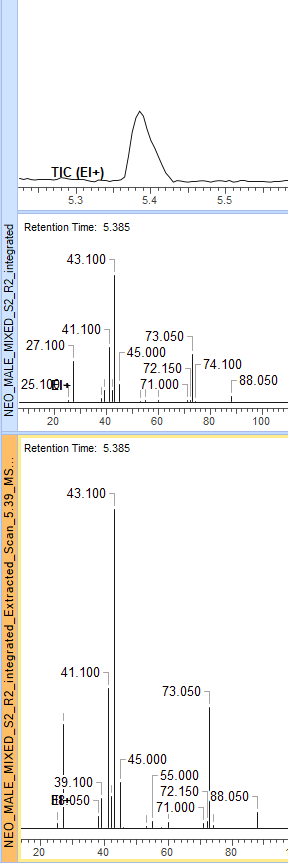 |
| 738 | 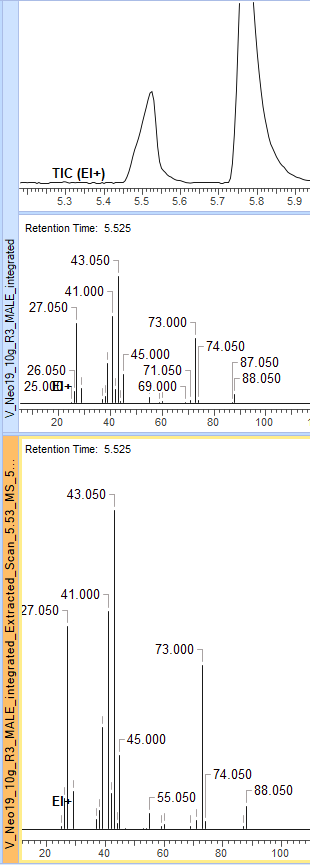 | 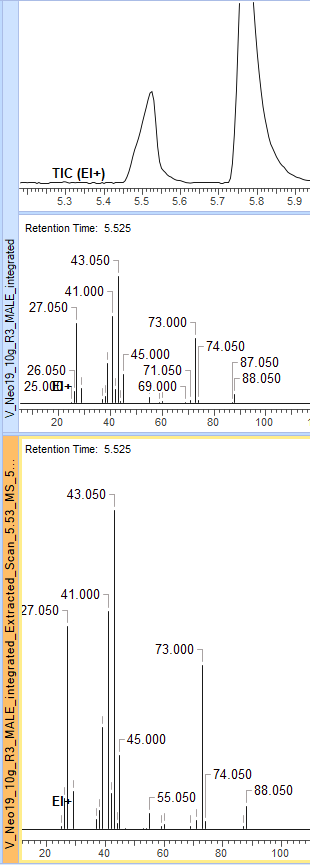 |
| 752  Ethyl 2-methyl-  propanoate | 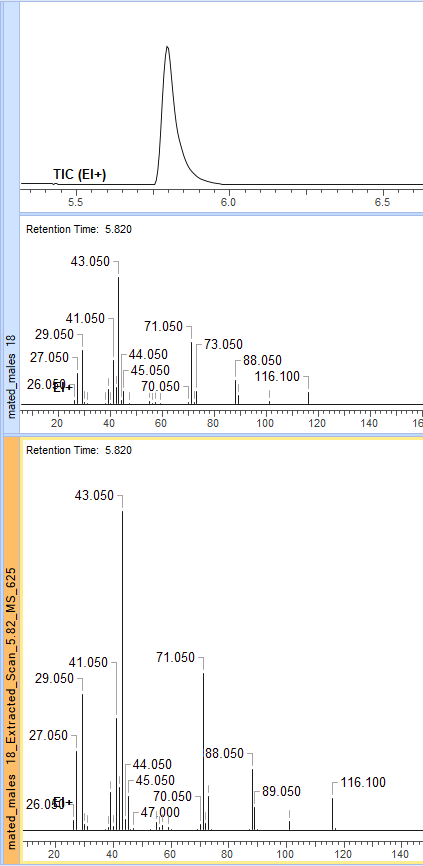 | 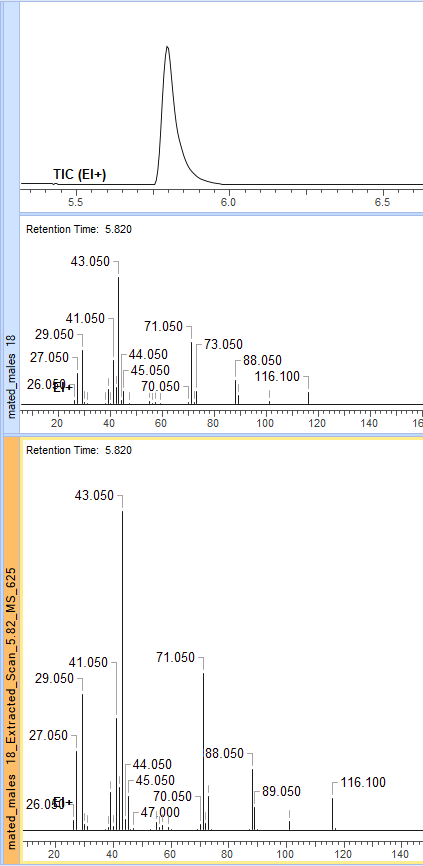 |
| 786  (*D*,*L*)- 2,3-Butanediol | 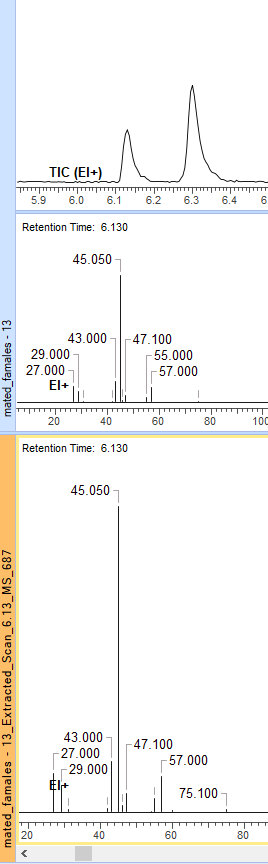 | 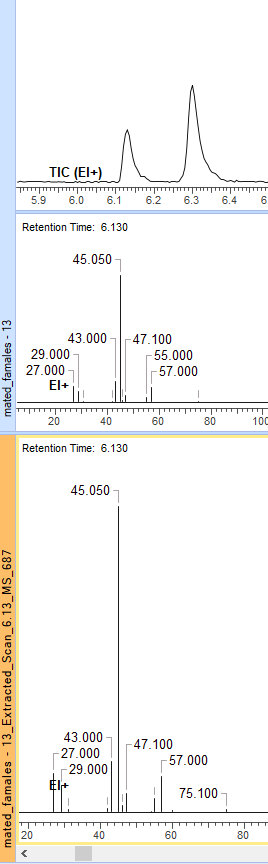 |
| 789  (*meso*)- 2,3-Butanediol | 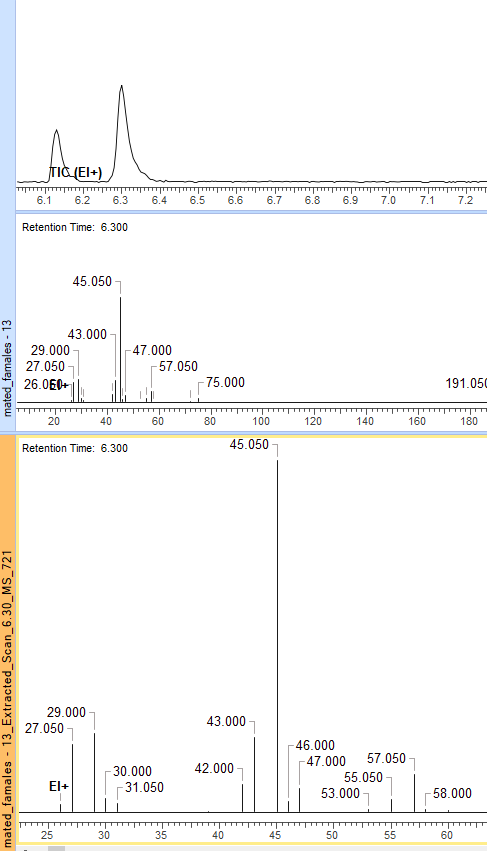 | 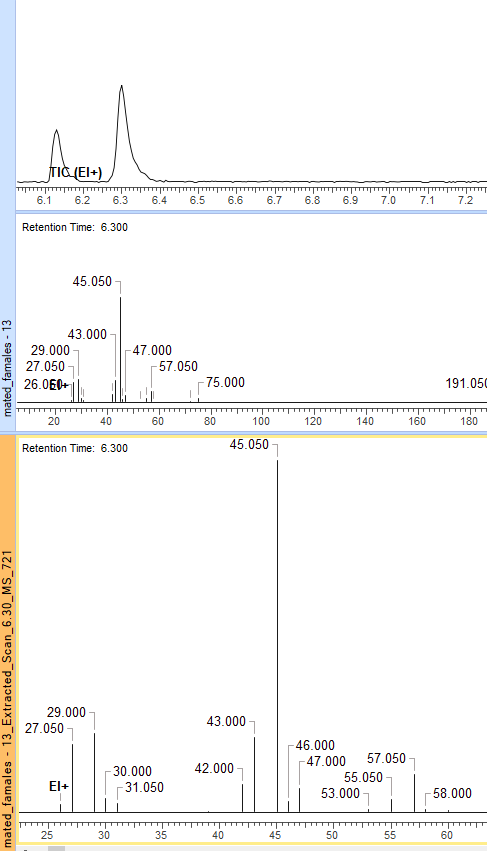 |
| 795 | 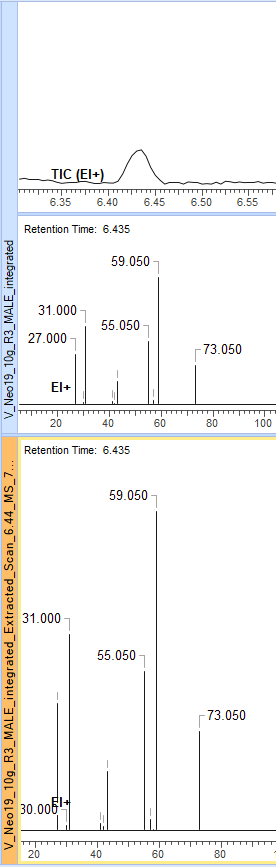 | 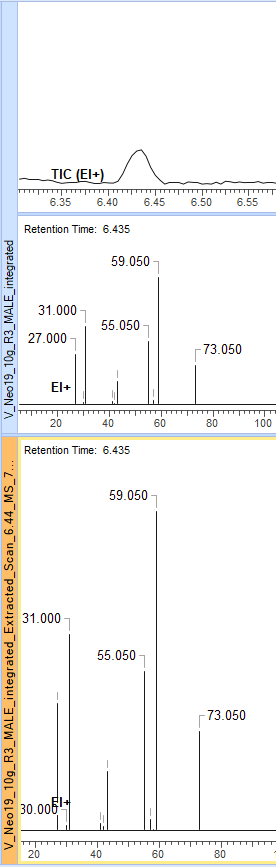 |
| 799 | 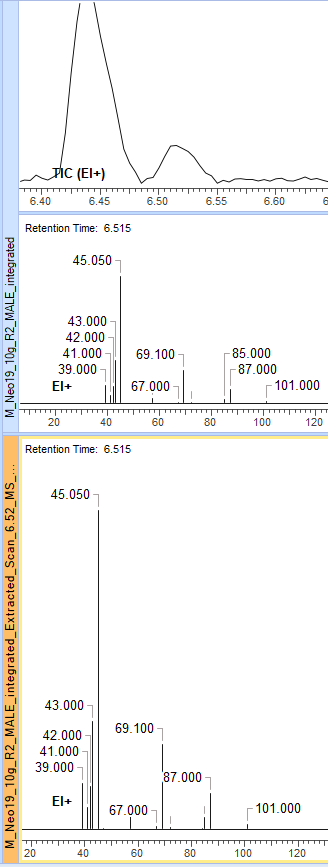 | 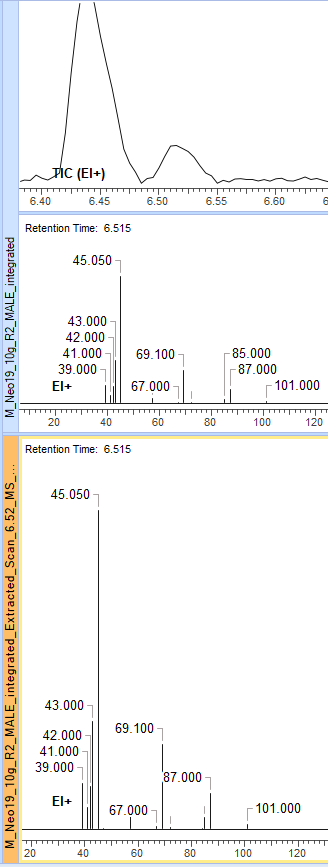 |
| 831  2-Methyl butanoic acid | 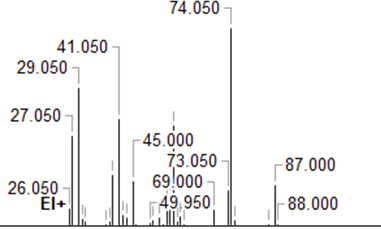 | 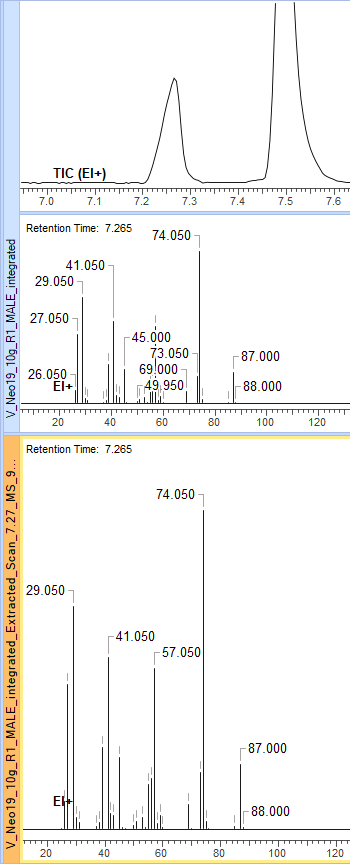 |
| 843  Ethyl 2-methyl-  butanoate | 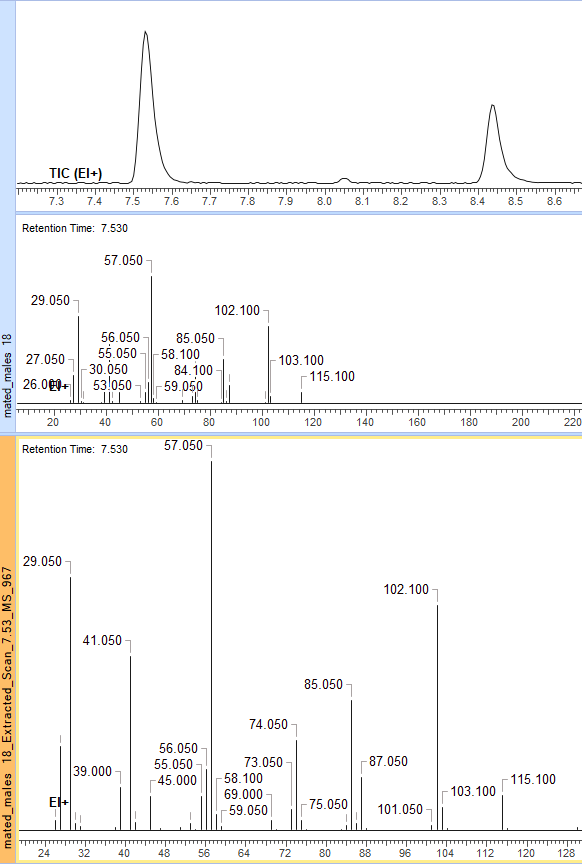 | 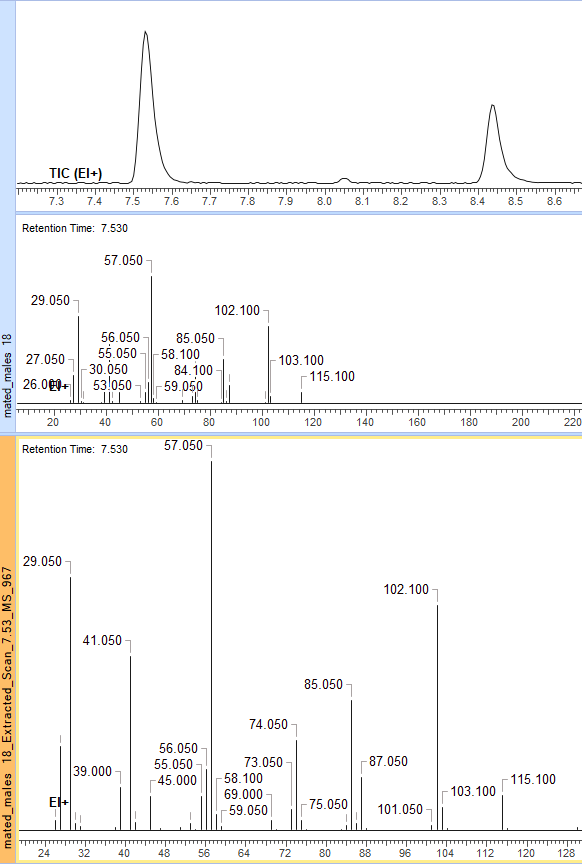 |
| 848  *n*-Propyl 3-methylpropanoate | 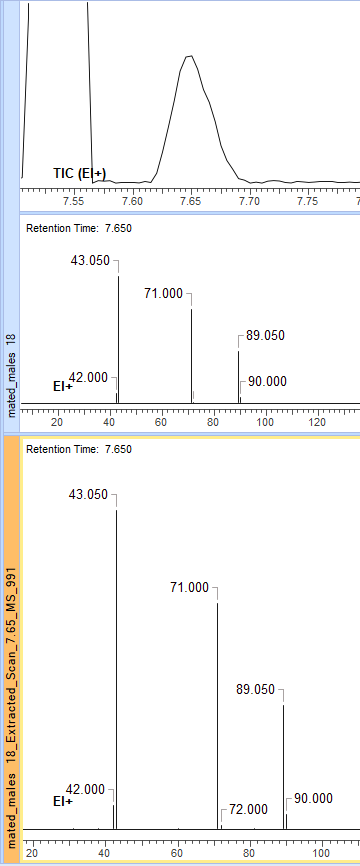 | 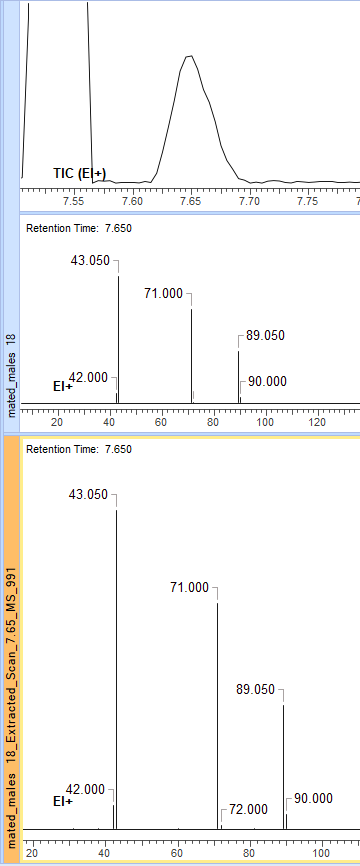 |
| 867  4-Heptanone | 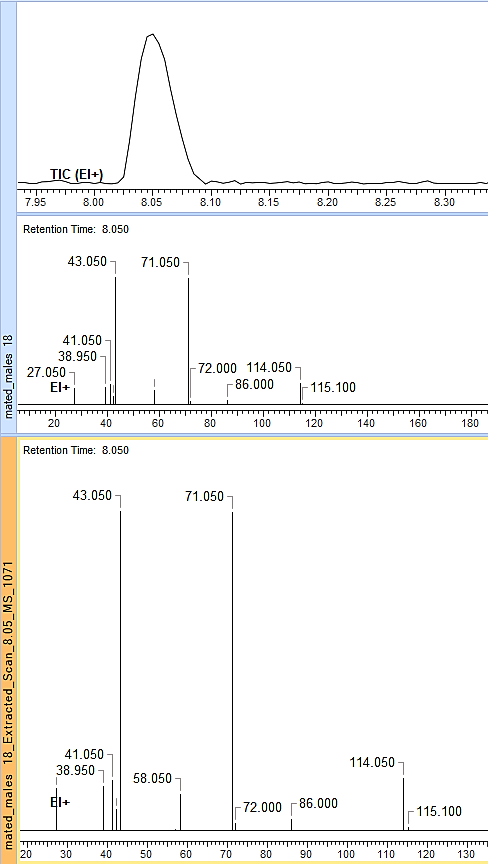 | 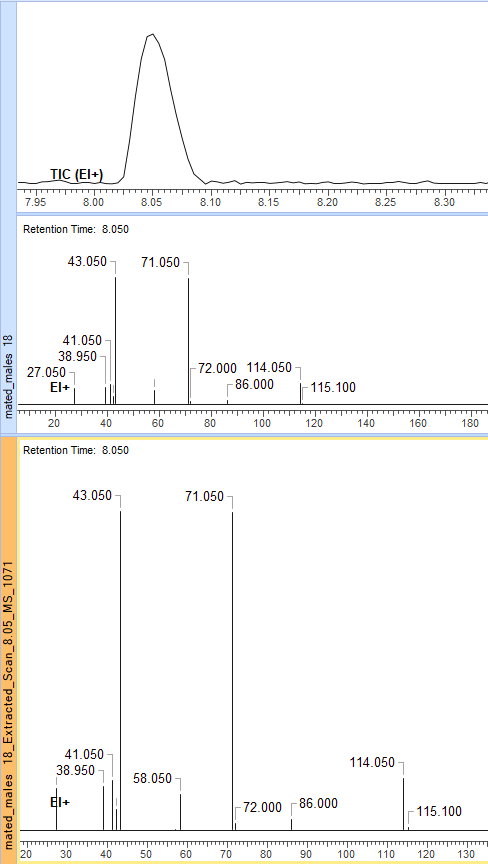 |
| 883  2-Methyl 3-hexanol | 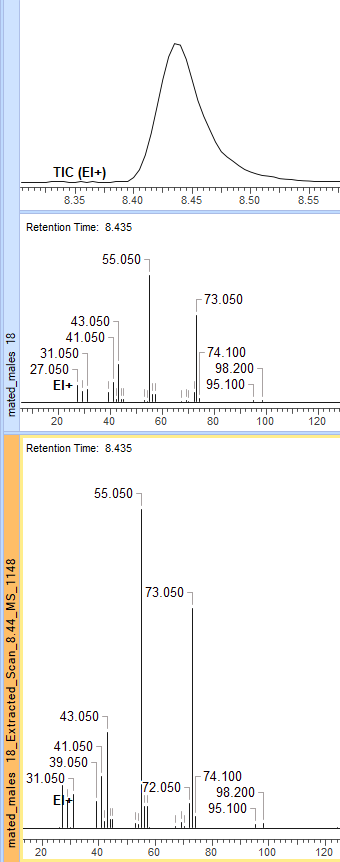 | 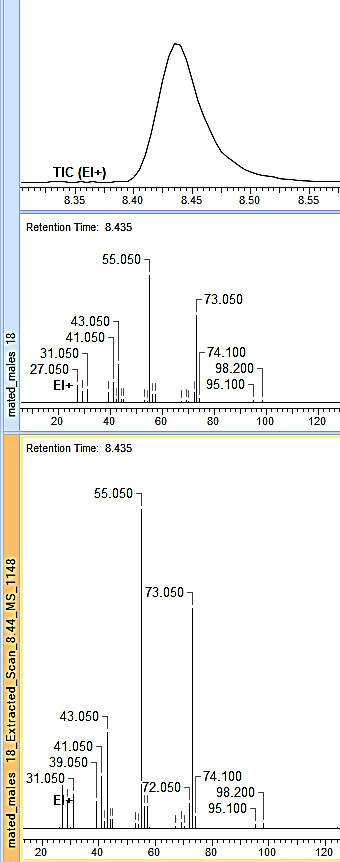 |
| 902  *x*-Octenal isomer 1 | 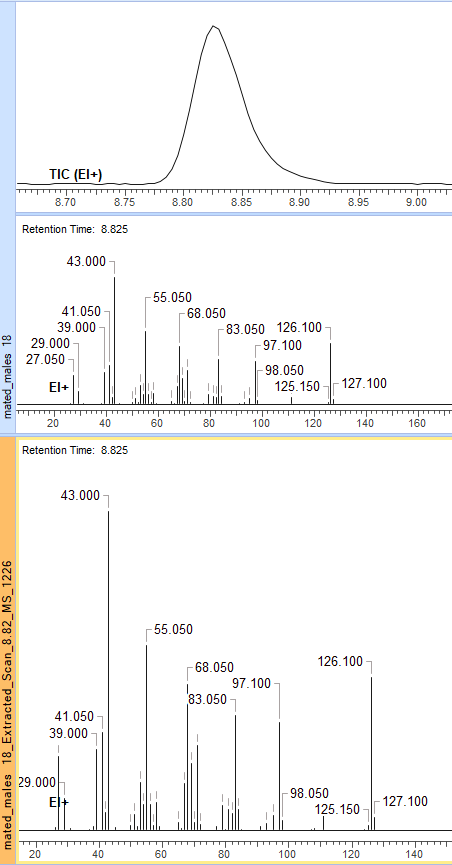 | 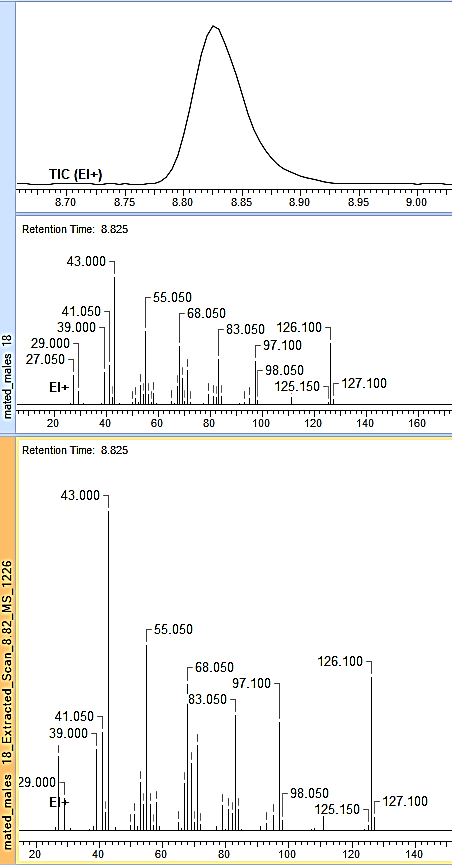 |
| 933  Ethyl 2-methylpentanoate | 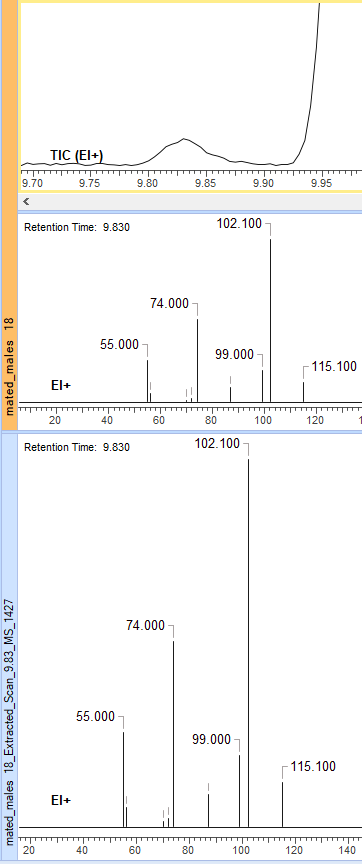 | 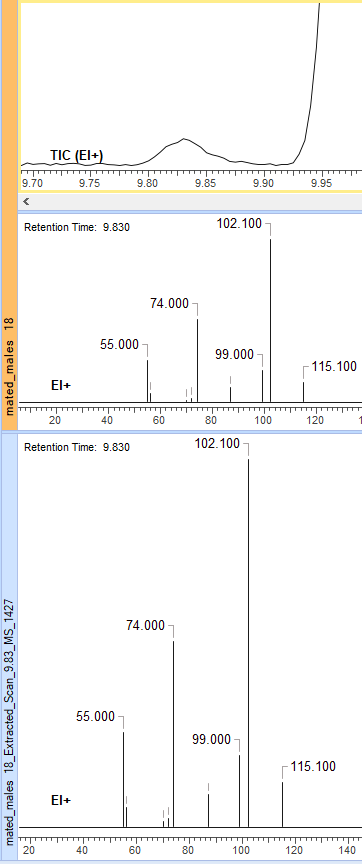 |
| 939  *x*-Octenal isomer 2 | 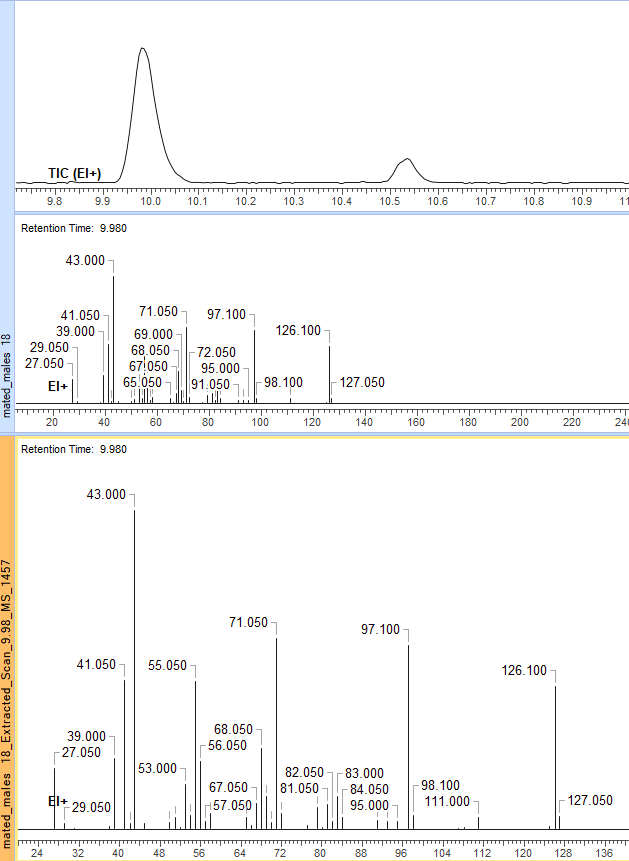 | 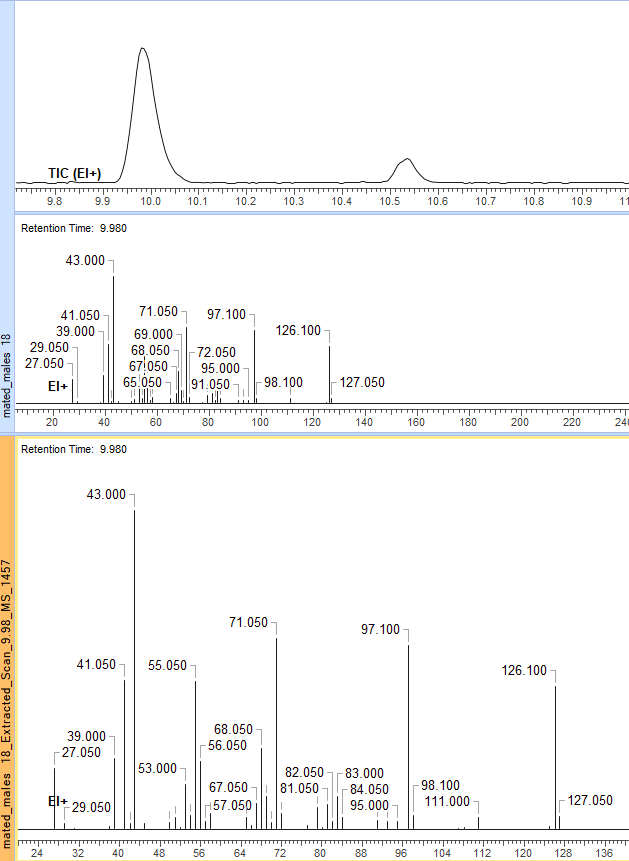 |
| 951 | 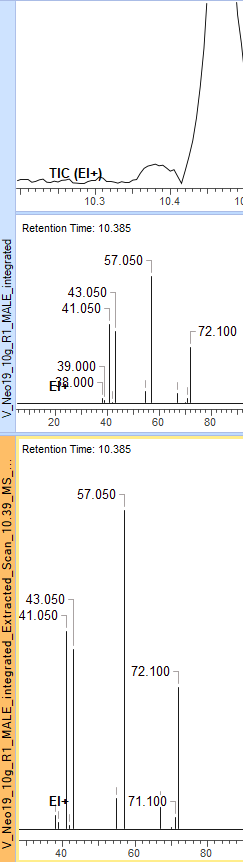 | 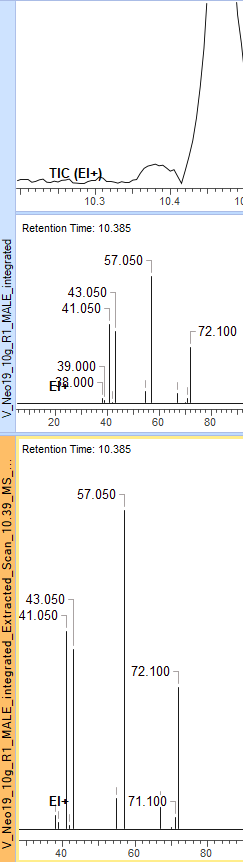 |
| 955  *n*-Butyl cyclopentane | 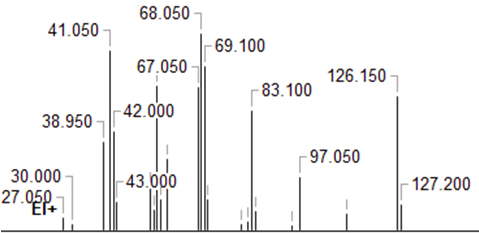 | 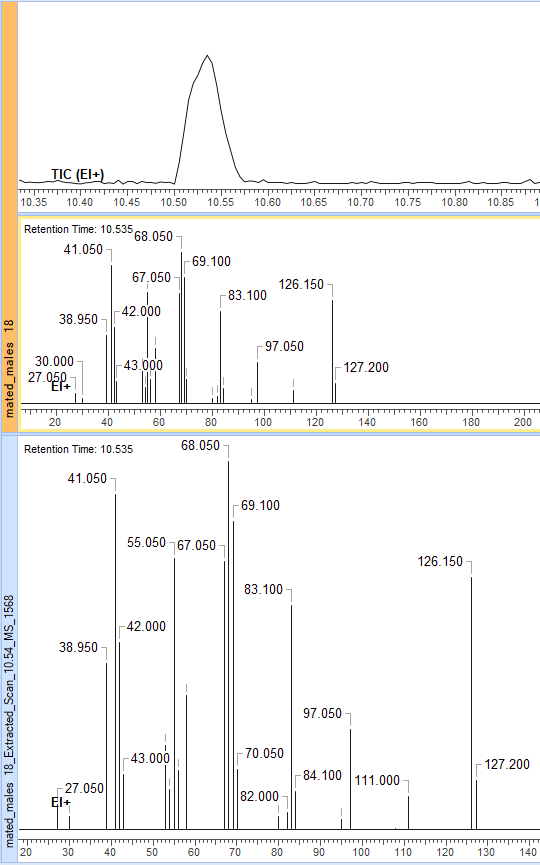 |
| 962 | 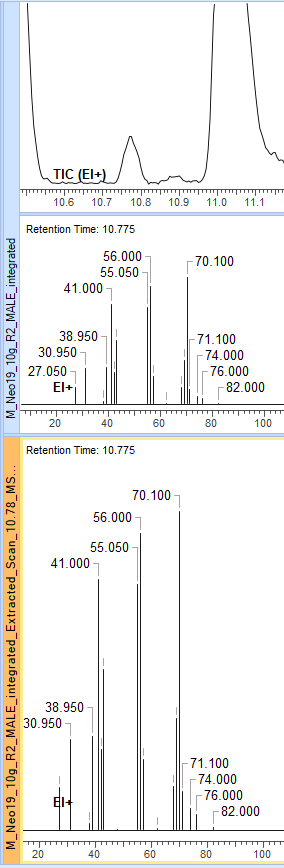 | 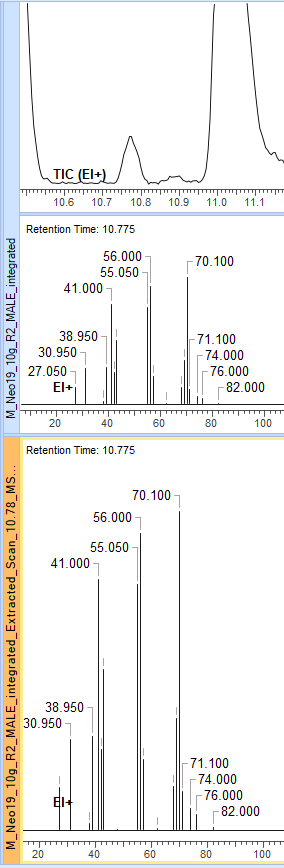 |
| 966 | 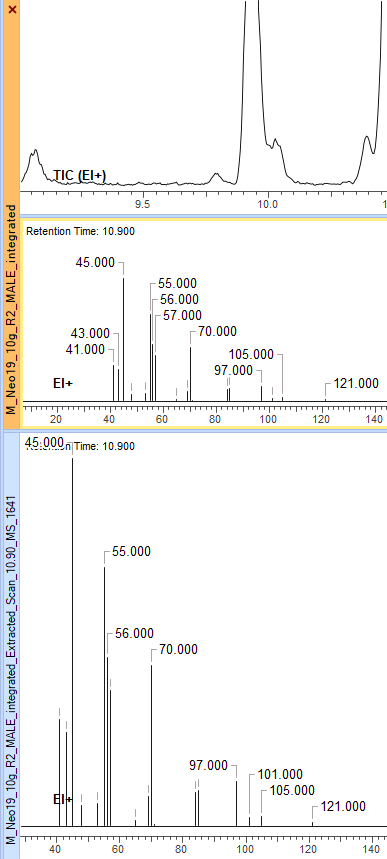 | 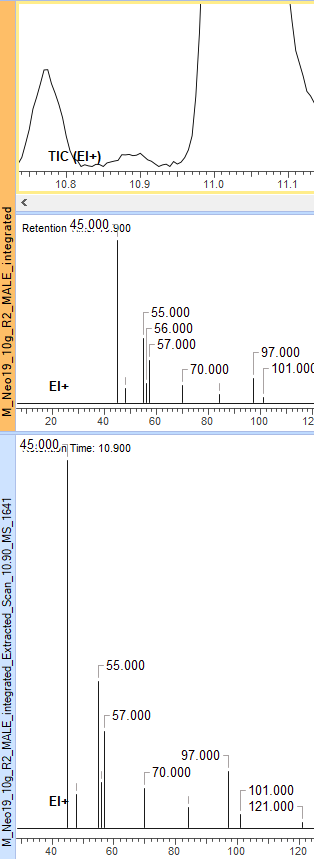 |
| 972  Phenol | 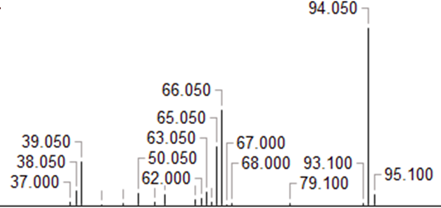 | 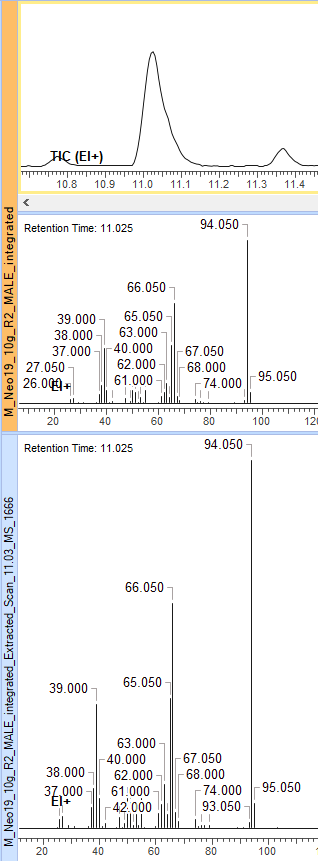 |
| 981  2-Ethyl-4-methyl-1-pentanol | 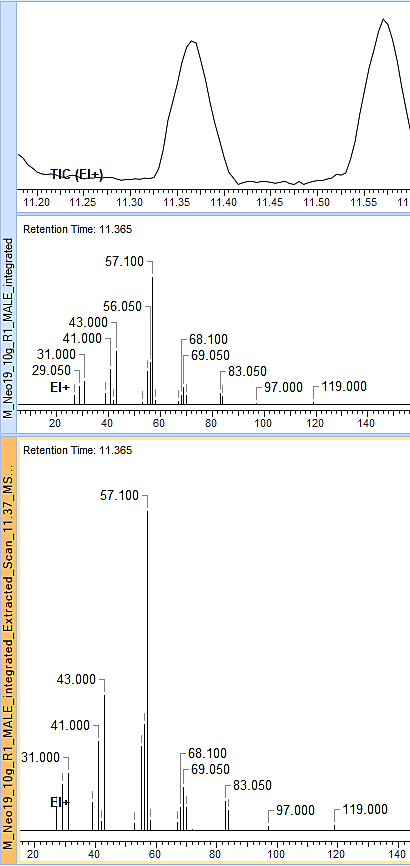 | 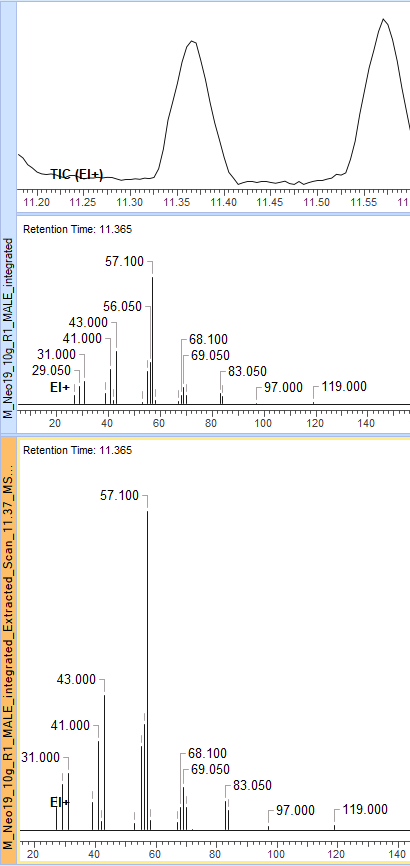 |
| 989 | 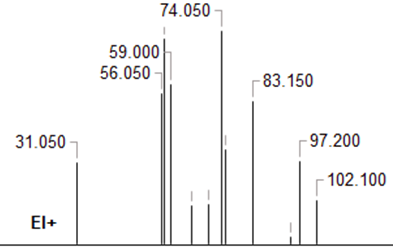 | 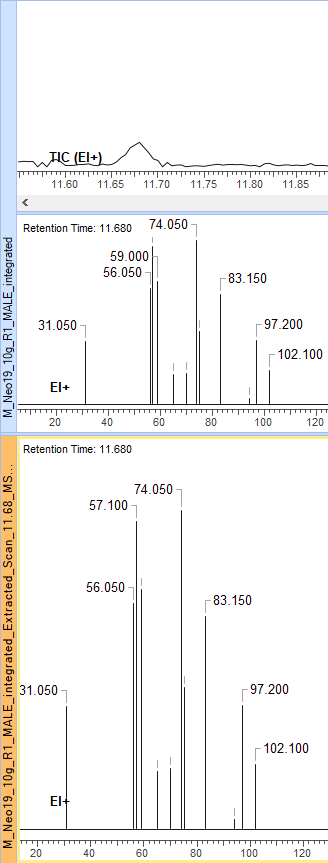 |
| 992 | 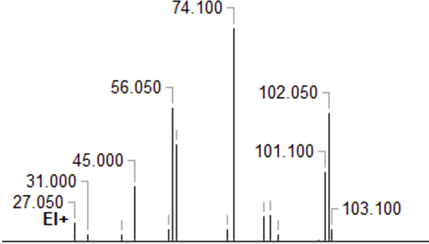 | 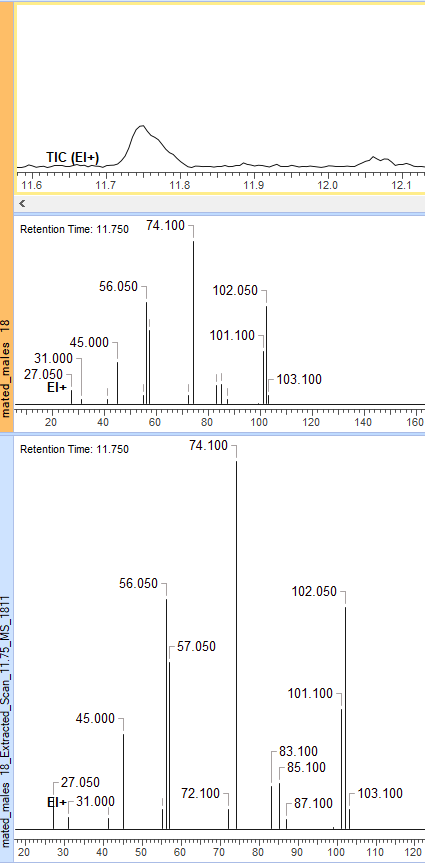 |
| 993 | 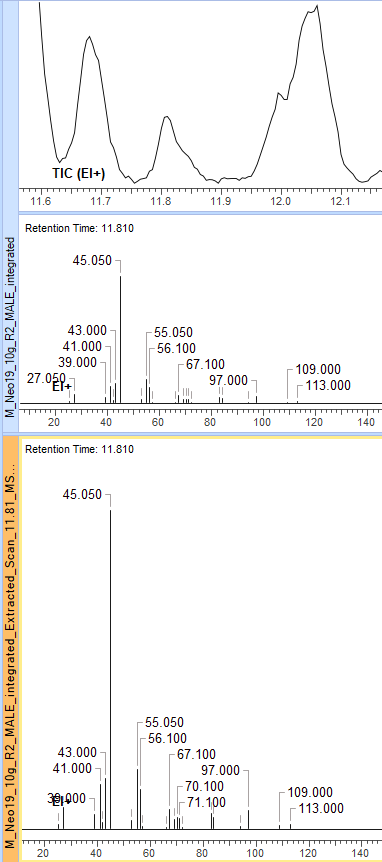 | 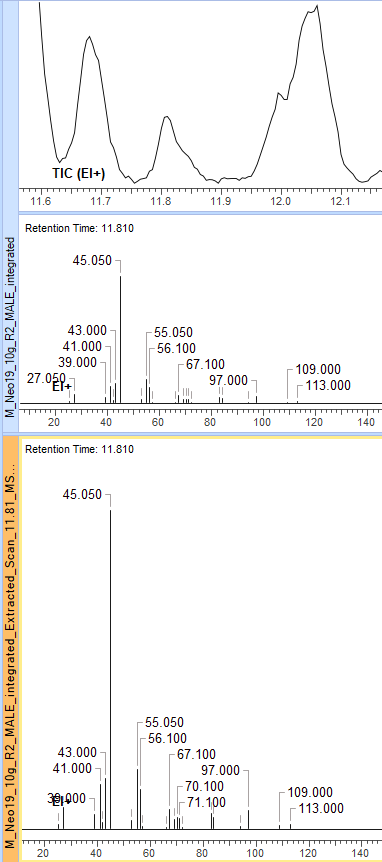 |
| 1001 | 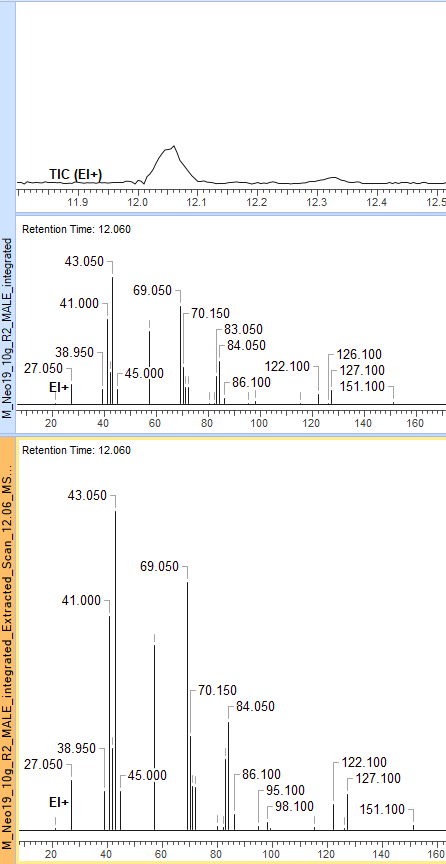 | 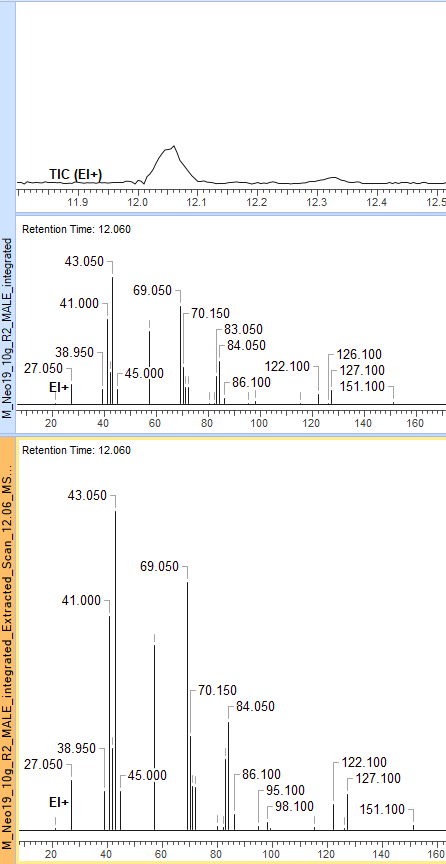 |
| 1006 | 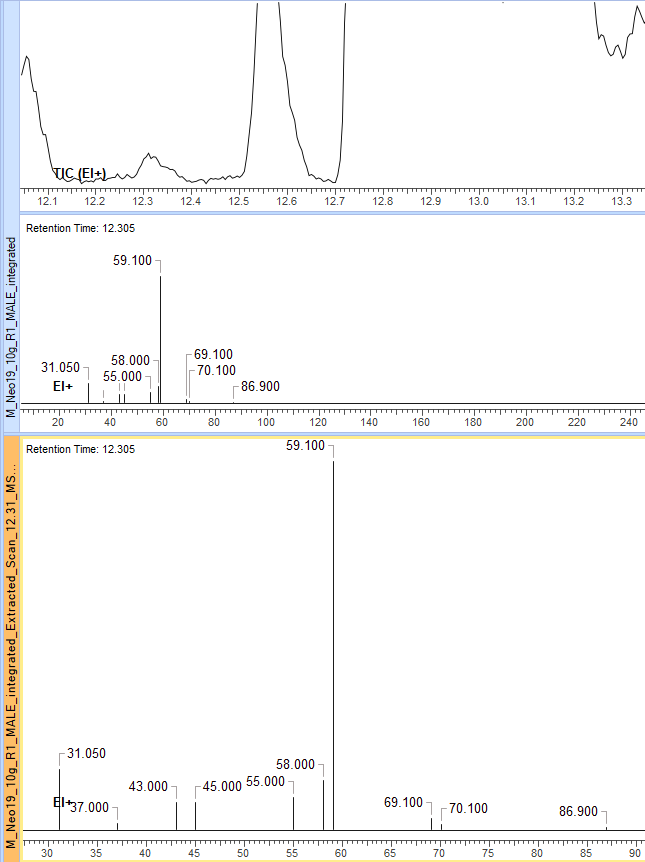 | 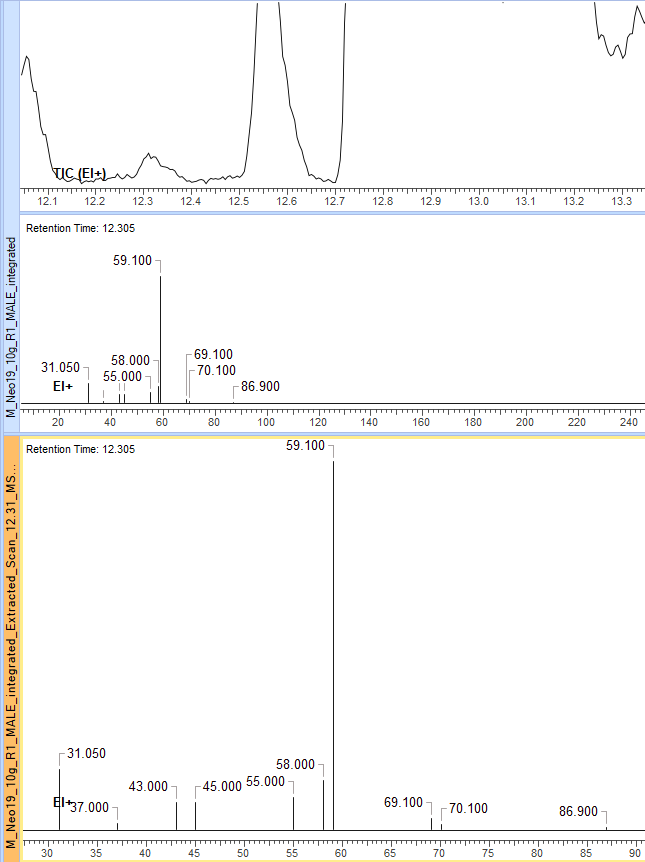 |
| 1019  *n*-Octen-1-ol | 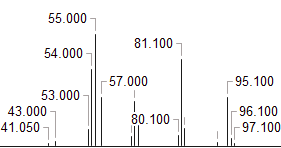 | 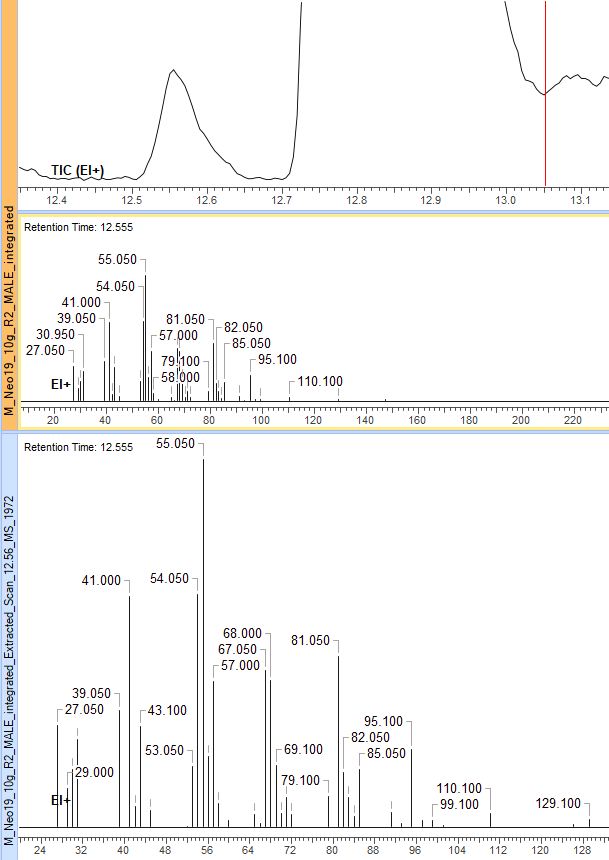 |
| 1026  2-Ethyl-1-hexanol | 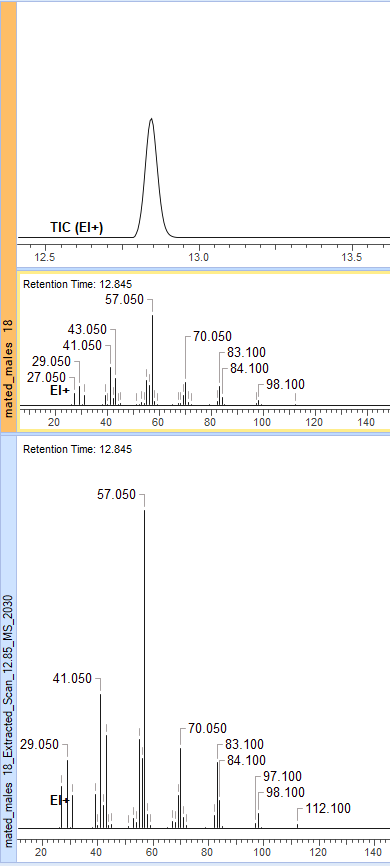 | 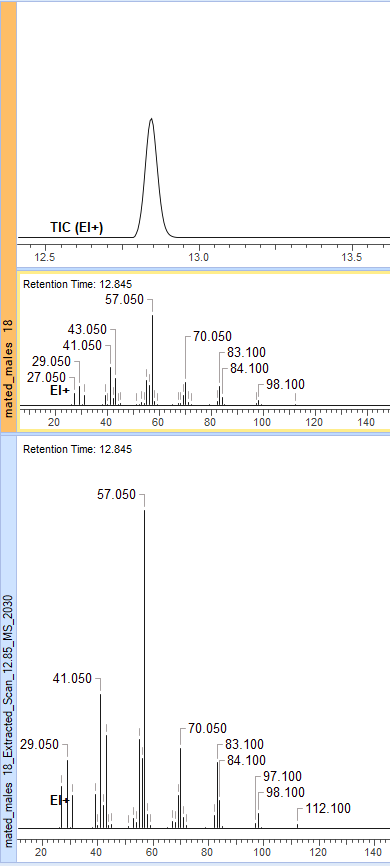 |
| 1028 | 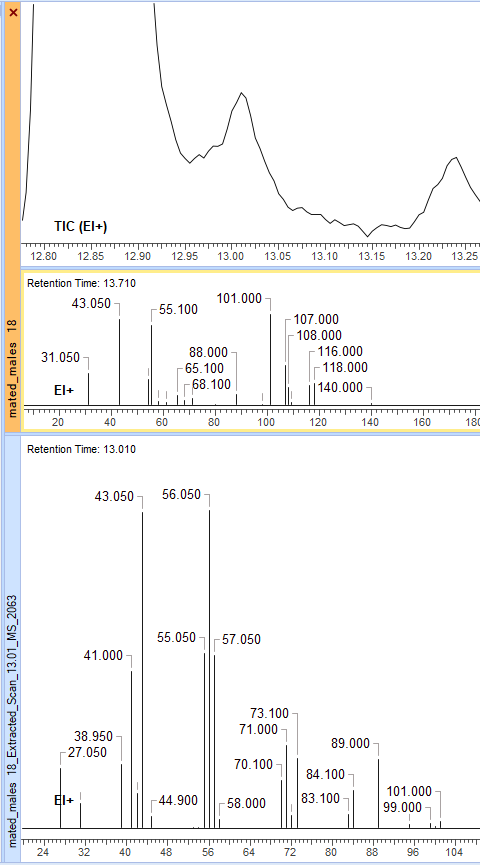 | 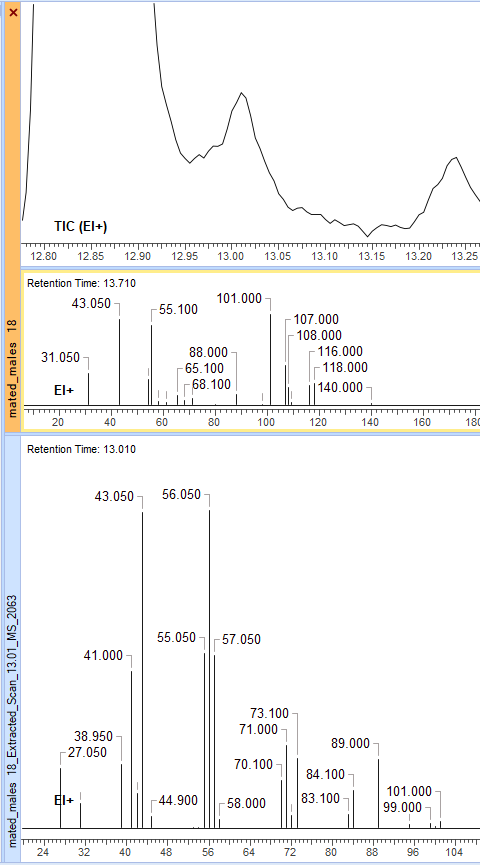 |
| 1059 | 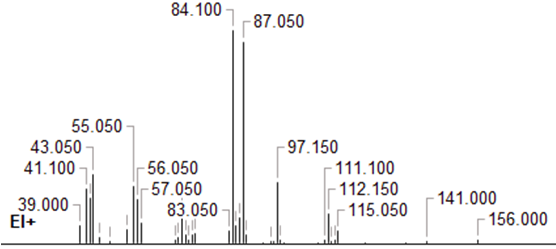 | 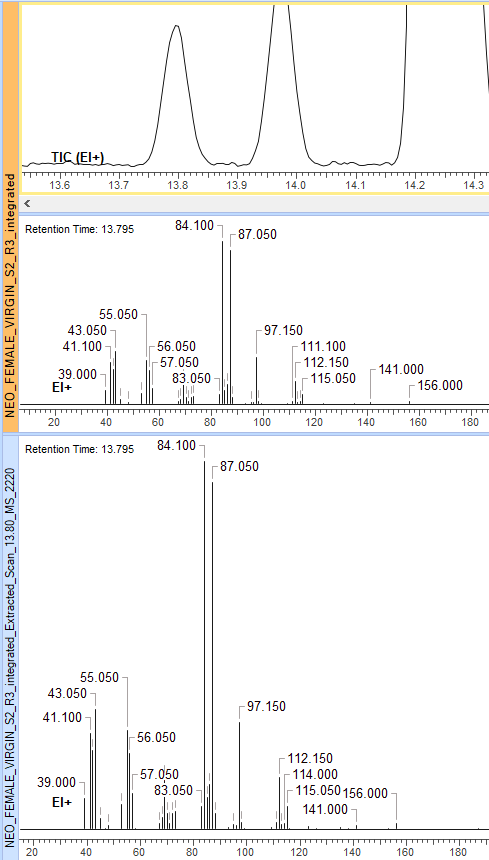 |
| 1064 | 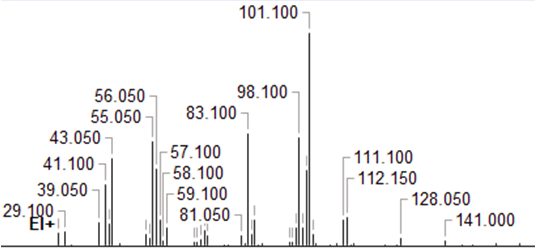 | 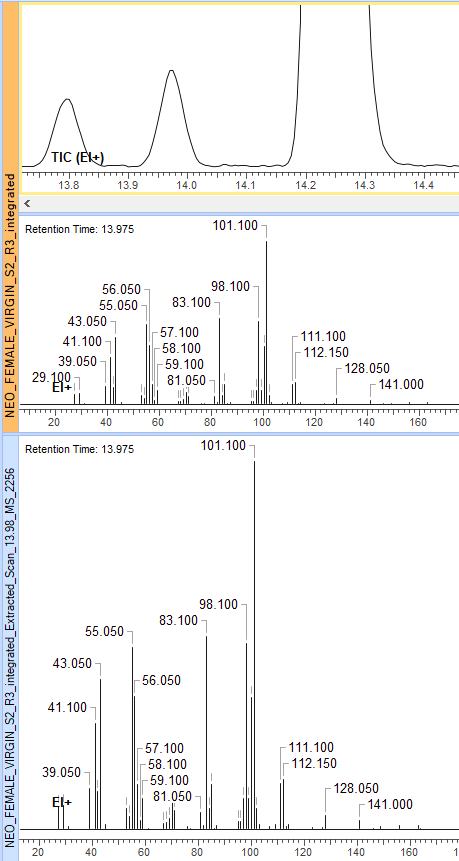 |
| 1072 | 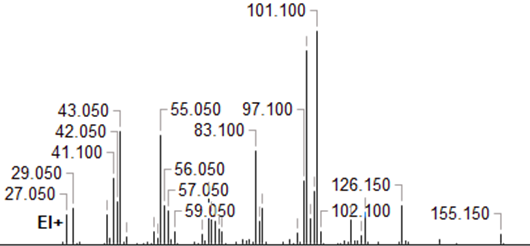 | 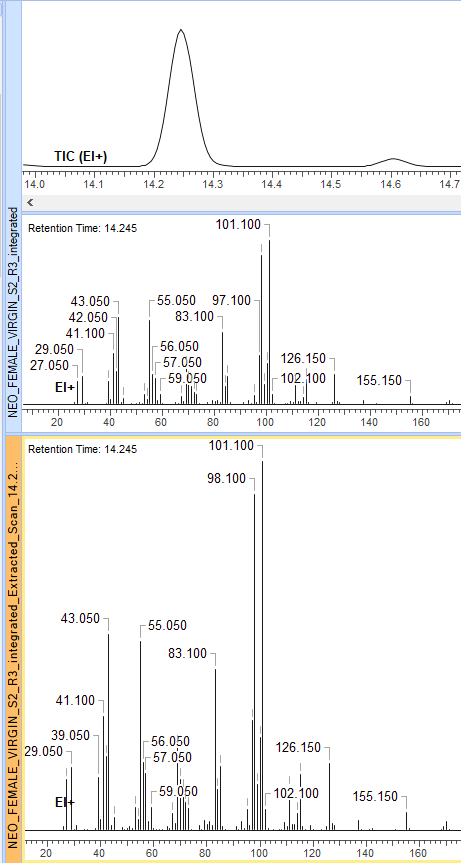 |
| 1083 | 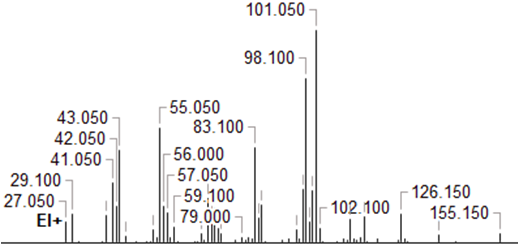 | 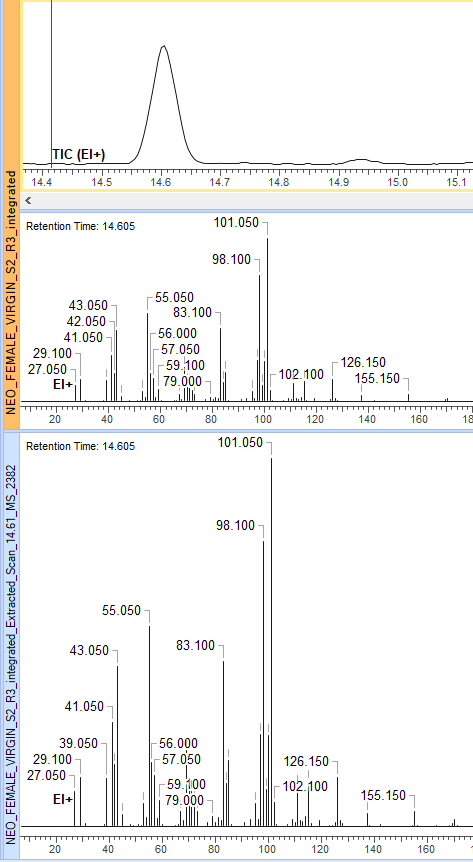 |
| 1087 | 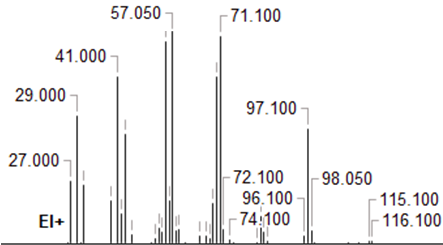 | 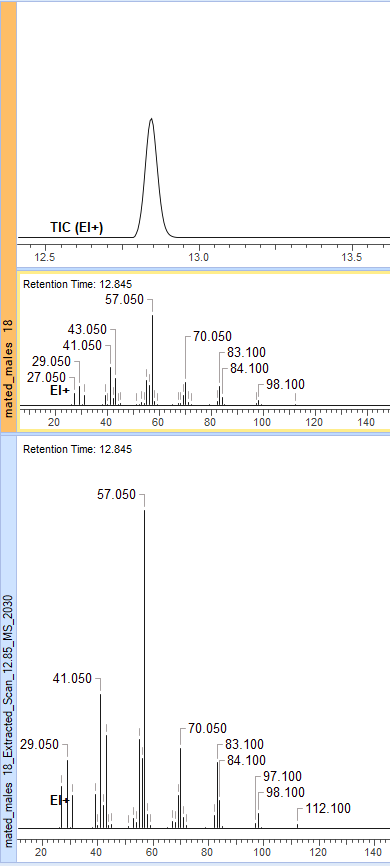 |
| 1094  *N*-(2-Methylpropyl)  propanamide | 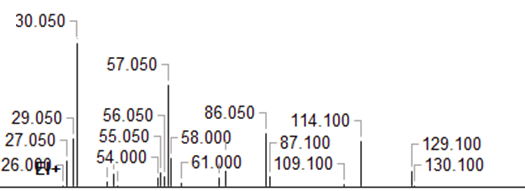 | 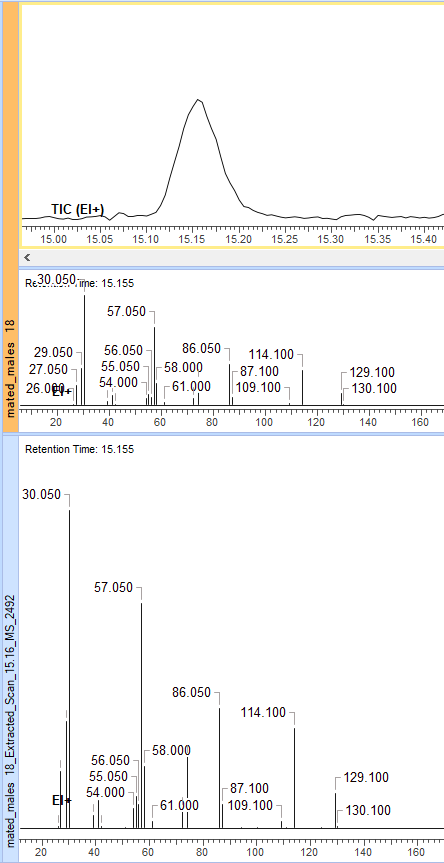 |
| 1098 | 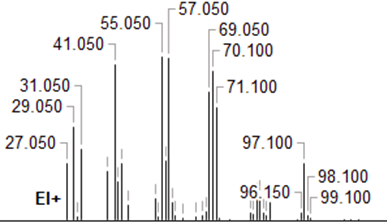 | 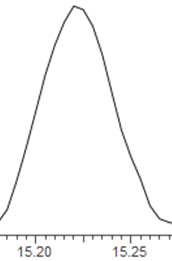 |
| 1108 | 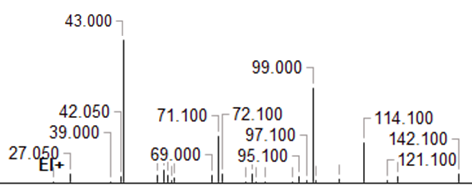 | 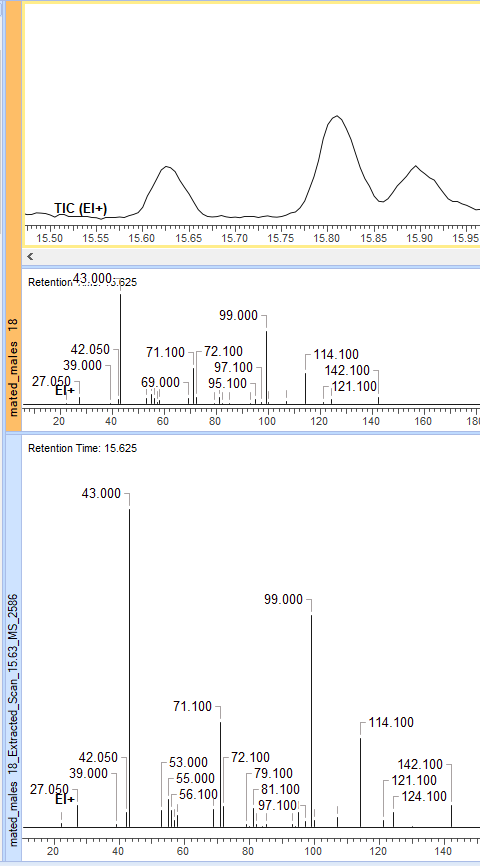 |
| 1115 | 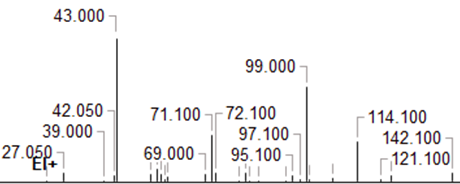 | 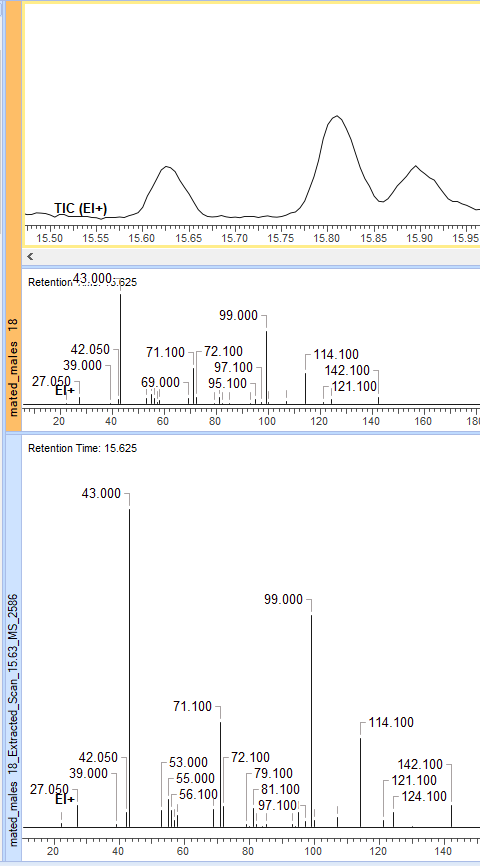 |
| 1123 | 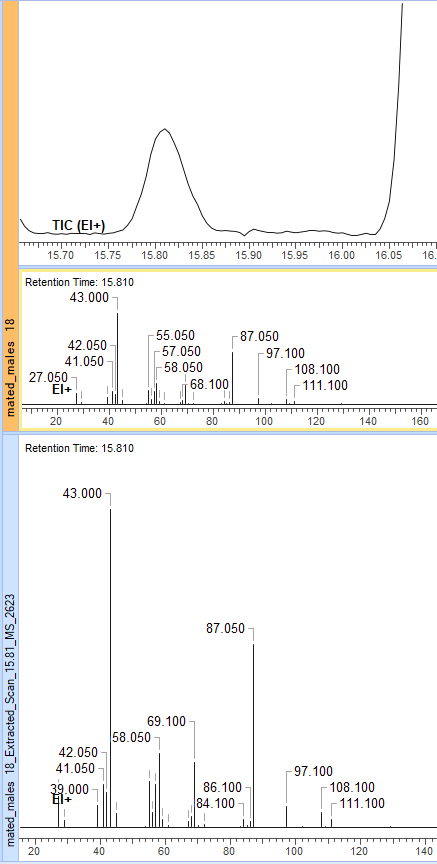 | 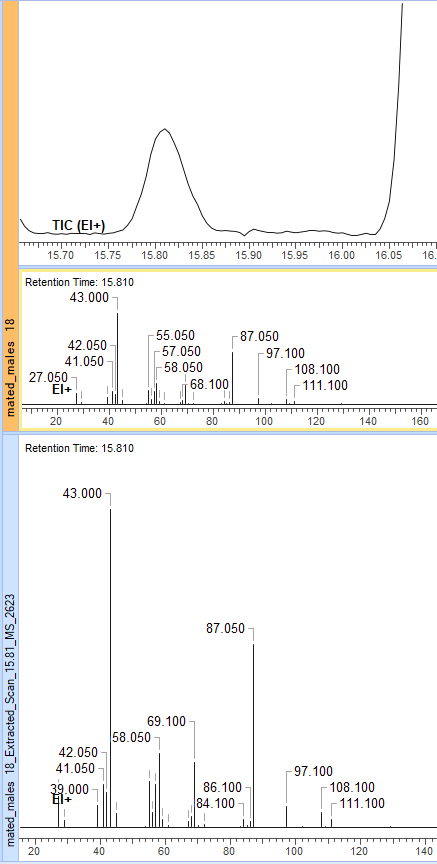 |
| 1124 | 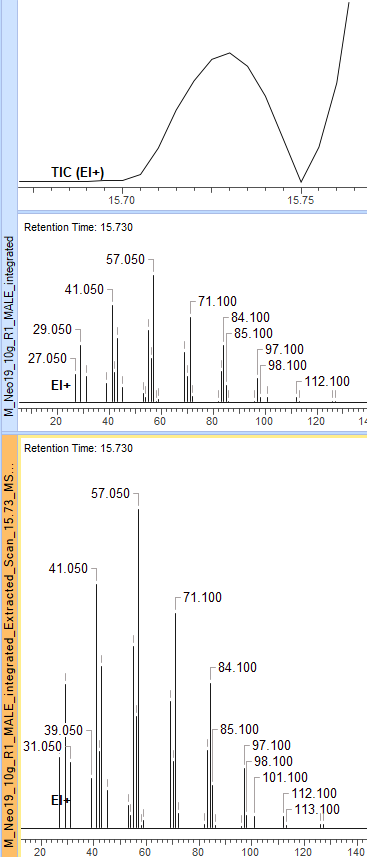 | 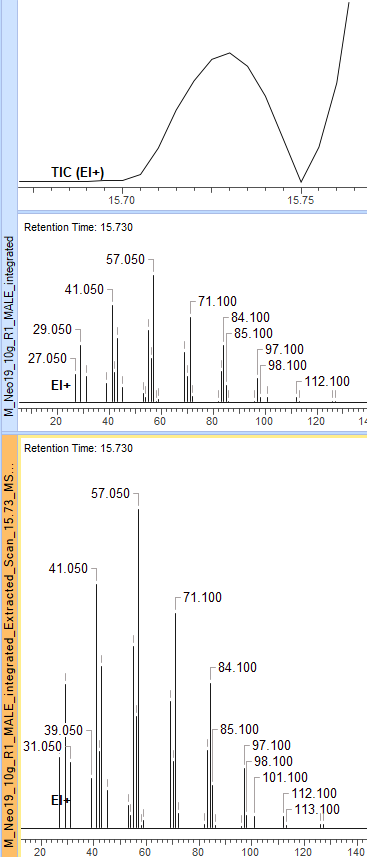 |
| 1127 | 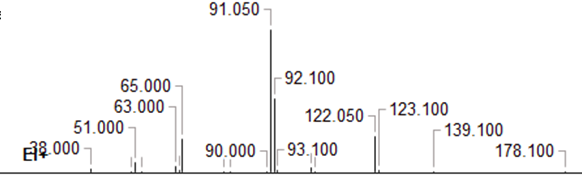 | 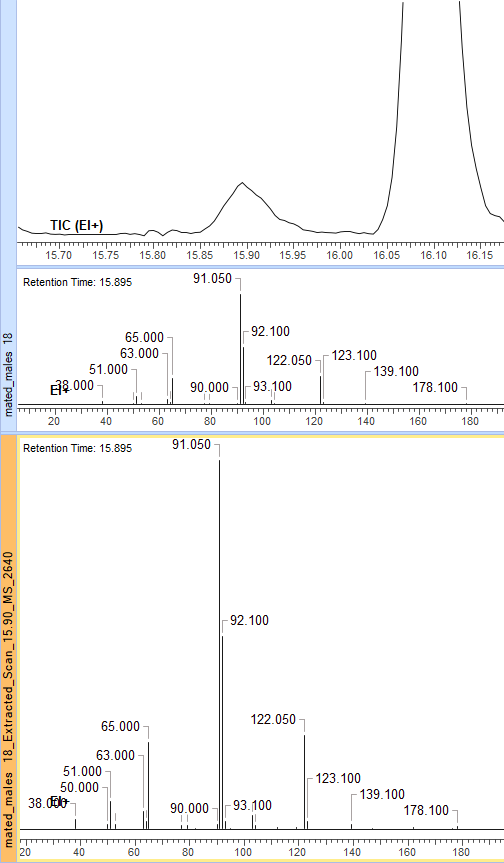 |
| 1132  *N*-(2-Methylbutyl)  acetamide | 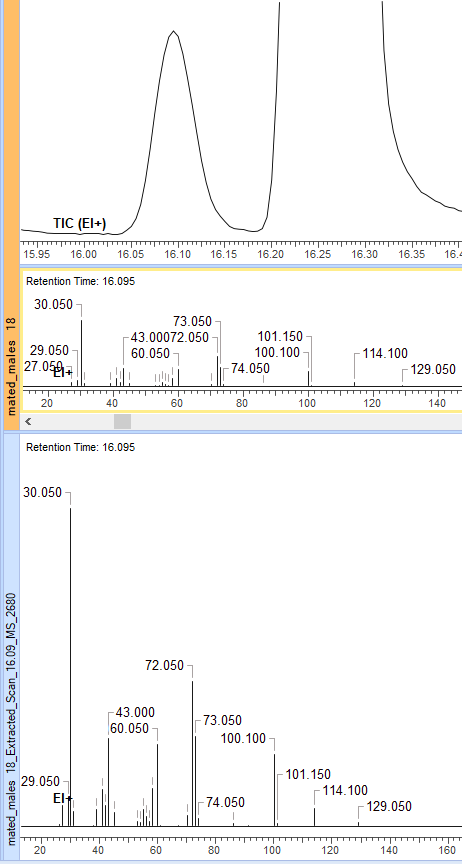 | 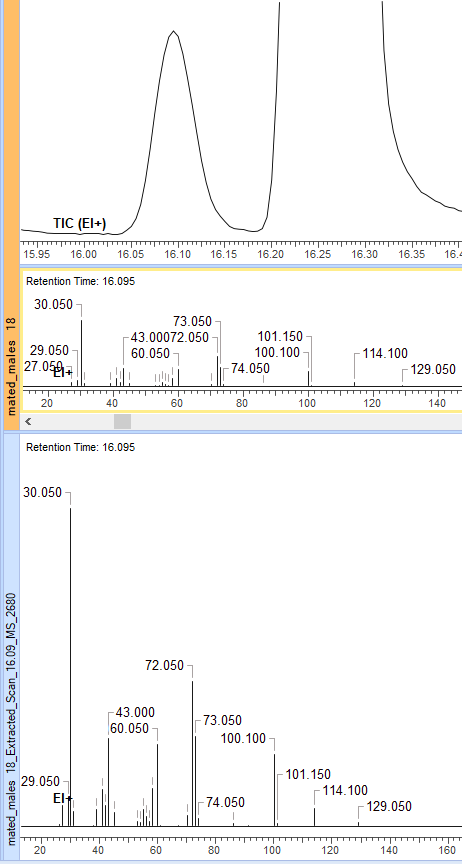 |
| 1142  *N*-(3-Methylbutyl)  acetamide | 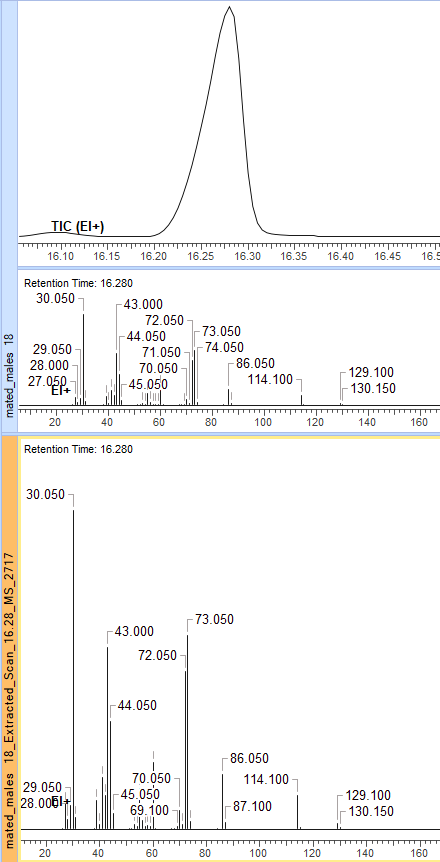 | 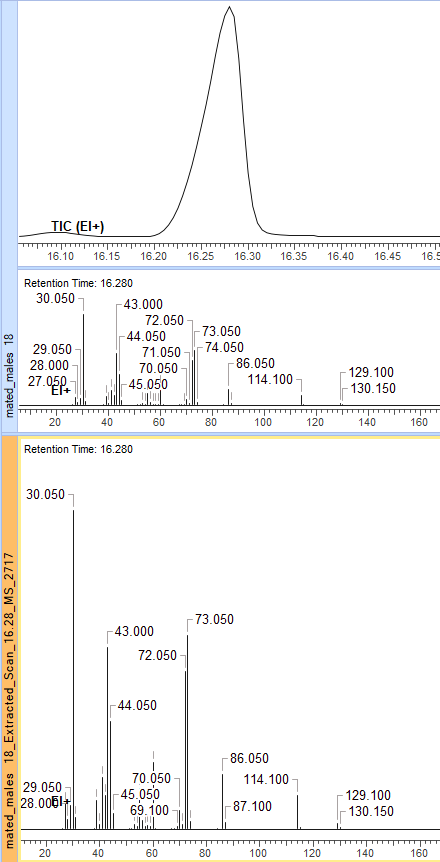 |
| 1157  2-Methyl-1,6-dioxaspiro[4.5]  decane | 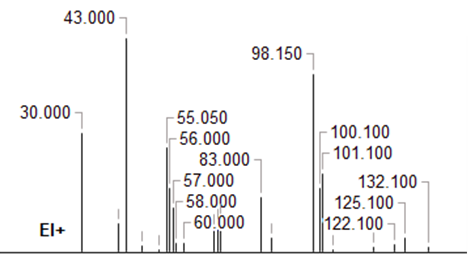 | 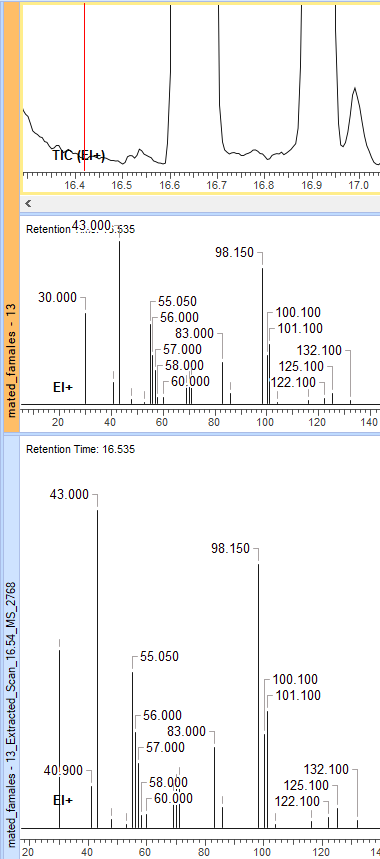 |
| 1159  (*E,E*)-2,8-Dimethyl-1,7-dioxaspiro[5.5]  undecane | 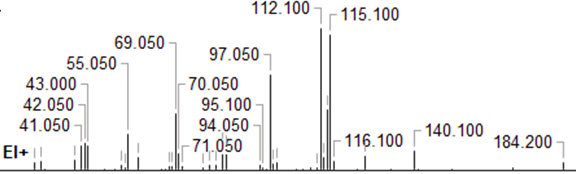 | 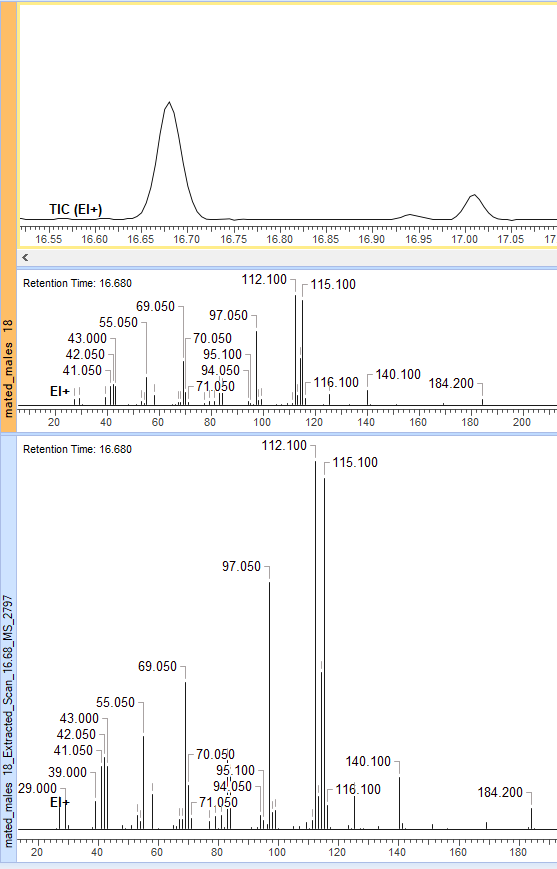 |
| 1164 |  |  |
| 1171  2-Bornanone |  |  |
| 1172  2,7-Dimethyl-1,6-dioxaspiro[4.5]  decane isomer 1 |  |  |
| 1173 |  |  |
| 1174  2-Ethyl-7-methyl-1,6-dioxaspiro[4.5]decane isomer 1 |  |  |
| 1175 |  |  |
| 1178 |  |  |
| 1179  Borneol isomer 1 |  |  |
| 1182  Diethyl succinate |  |  |
| 1183 |  |  |
| 1184 |  |  |
| 1185 |  |  |
| 1187  Borneol isomer 2 |  |  |
| 1188 |  |  |
| 1189 |  |  |
| 1191 |  |  |
| 1194 |  |  |
| 1201  *N*-(2-Methylbutyl)  propanamide |  |  |
| 1212  *N*-(3-Methylbutyl)  propanamide |  |  |
| 1216 |  |  |
| 1219 |  |  |
| 1226 |  |  |
| 1235  *N*-(2-Methylbutyl)  propanamide |  |  |
| 1239  *N*-(3-Methylbutyl)  propanamide |  |  |
| 1244  2-Ethyl-8-methyl-1,7-dioxaspiro[5.5]undecane |  |  |
| 1253 |  |  |
| 1256 |  |  |
| 1267 |  |  |
| 1285 |  |  |
| 1292 |  |  |
| 1296 |  |  |
| 1303 |  |  |
| 1306 |  |  |
| 1327 |  |  |
| 1334 |  |  |
| 1340 |  |  |
| 1357 |  |  |
| 1370 |  |  |
| 1375 |  |  |
| 1382 |  |  |
| 1388 |  |  |
| 1399 |  |  |
| 1412 |  |  |
| 1434 |  |  |
| 1442 |  |  |
| 1446 |  |  |
| 1454 |  |  |
| 1461 |  |  |
| 1464 |  |  |
| 1468 |  |  |
| 1473 |  |  |
| 1484 |  |  |
| 1492 |  |  |
| 1499 |  |  |
| 1525  Methyl dodecanoate |  |  |
| 1551 |  |  |
| 1583  Ethyl (*Z*)-9-dodecenoate |  |  |
| 1592  Ethyl dodecanoate |  |  |
| 1596 |  |  |
| 1609 |  |  |
| 1635 |  |  |
| 1659 |  |  |
| 1675  *n*-Propyl dodecanoate |  |  |
| 1716  Methyl (*Z*)-9-tetradecenoate |  |  |
| 1723  Methyl tetradecanoate |  |  |
| 1760 |  |  |
| 1783  Ethyl (*E*)-9-tetradecenoate |  |  |
| 1788  Ethyl tetradecanoate |  |  |
| 1847 |  |  |
| 1868  Ethyl 12-methyltetradecanoate |  |  |
| 1889  *n*-Propyl tetradecanoate |  |  |
| 1911  Methyl (*Z*)-9-hexadecenoate |  |  |
| 1928  Methyl hexadecanoate |  |  |
| 1981  Ethyl (*Z*)-9-hexadecenoate |  |  |
| 1989  Ethyl hexadecanoate |  |  |
| 2188  Ethyl (*E*)-9-octadecenoate |  |  |

**Supplementary Table S1**

| **Species, strain and sex specificity** | | **Mating history specificity** | ***try*** | | | | | | | | ***neo*** | | | | | | | |  | | |
| --- | --- | --- | --- | --- | --- | --- | --- | --- | --- | --- | --- | --- | --- | --- | --- | --- | --- | --- | --- | --- | --- |
| **Found in** | **but missing in** | **also missing in** | **New** | | | | **Old** | | | | **New** | | | | **Old** | | | |  | | |
|  |  |  | **♂** | | **♀** | | **♂** | | **♀** | | **♂** | | **♀** | | **♂** | | **♀** | |  | | |
|  |  |  | **V** | **M** | **V** | **M** | **V** | **M** | **V** | **M** | **V** | **M** | **V** | **M** | **V** | **M** | **V** | **M** | **n** | **KI** | **sum** |
| Both spp, both str, both sex | - | no | ✓ | ✓ | ✓ | ✓ | ✓ | ✓ | ✓ | ✓ | ✓ | ✓ | ✓ | ✓ | ✓ | ✓ | ✓ | ✓ | 11 | 1026, 1028, 1132, 1142, 1159, 1175, 1201, 1212, 1235, 1239, 1244 | 16 |
| Both spp, both str, both sex | - | V *try* New mals |  | ✓ | ✓ | ✓ | ✓ | ✓ | ✓ | ✓ | ✓ | ✓ | ✓ | ✓ | ✓ | ✓ | ✓ | ✓ | 1 | 1019 | 15 |
| Both spp, both str, both sex | - | M *try* Old fems | ✓ | ✓ | ✓ | ✓ | ✓ | ✓ | ✓ |  | ✓ | ✓ | ✓ | ✓ | ✓ | ✓ | ✓ | ✓ | 3 | 1442, 1454, 1484 | 15 |
| Both spp, both str, both sex | - | V *neo* New fems | ✓ | ✓ | ✓ | ✓ | ✓ | ✓ | ✓ | ✓ | ✓ | ✓ |  | ✓ | ✓ | ✓ | ✓ | ✓ | 1 | 939 | 15 |
| Both spp, both str, both sex | - | V *neo* Old mals | ✓ | ✓ | ✓ | ✓ | ✓ | ✓ | ✓ | ✓ | ✓ | ✓ | ✓ | ✓ |  | ✓ | ✓ | ✓ | 1 | 1216 | 15 |
| Both spp, both str, both sex | - | V *neo* New mals, V *neo* Old mals | ✓ | ✓ | ✓ | ✓ | ✓ | ✓ | ✓ | ✓ |  | ✓ | ✓ | ✓ |  | ✓ | ✓ | ✓ | 1 | 1219 | 14 |
| Both spp, both str, both sex | - | V *neo* Old mals, V *try* Old mals | ✓ | ✓ | ✓ | ✓ |  | ✓ | ✓ | ✓ | ✓ | ✓ | ✓ | ✓ |  | ✓ | ✓ | ✓ | 1 | 1357 | 14 |
| Both spp, both str, both sex | *try* New mals | V *neo* New mals |  |  | ✓ | ✓ | ✓ | ✓ | ✓ | ✓ |  | ✓ | ✓ | ✓ | ✓ | ✓ | ✓ | ✓ | 1 | 1583 | 13 |
| Both spp, both str, both sex | *try* New mals | V *neo* New mals, V *neo* Old mals |  |  | ✓ | ✓ | ✓ | ✓ | ✓ | ✓ |  | ✓ | ✓ | ✓ |  | ✓ | ✓ | ✓ | 2 | 1760, 1783 | 12 |
| Both spp, both str, both sex | *neo* Old mals | V *try* New mals, V *try* Old mals |  | ✓ | ✓ | ✓ |  | ✓ | ✓ | ✓ | ✓ | ✓ | ✓ | ✓ |  |  | ✓ | ✓ | 1 | 1296 | 12 |
| Both spp, both str, both sex | *neo* Old mals | M *neo* New fems, | ✓ | ✓ | ✓ | ✓ | ✓ | ✓ | ✓ | ✓ | ✓ | ✓ | ✓ |  |  |  | ✓ | ✓ | 2 | 674, 789 | 13 |
| Both spp, both str, both sex | *neo* New fems | V *try* New mals |  | ✓ |  |  | ✓ | ✓ | ✓ | ✓ | ✓ | ✓ | ✓ | ✓ | ✓ | ✓ | ✓ | ✓ | 1 | 1267 | 13 |
| Both spp, both str, both sex | *neo* Old fems | V *try* Old fems | ✓ | ✓ | ✓ | ✓ | ✓ | ✓ |  | ✓ | ✓ | ✓ | ✓ | ✓ | ✓ | ✓ |  |  | 1 | 1464 | 13 |
| Both spp, both str, both sex | *neo* New fems, *try* New mals | V *neo* Old fems, V *try* New fems |  |  |  | ✓ | ✓ | ✓ | ✓ | ✓ | ✓ | ✓ |  |  | ✓ | ✓ |  | ✓ | 1 | 972 | 10 |
| Both spp, both str, both sex | *neo* New fems, *neo* Old mals | no | ✓ | ✓ | ✓ | ✓ | ✓ | ✓ | ✓ | ✓ | ✓ | ✓ |  |  |  |  | ✓ | ✓ | 1 | 786 | 12 |
| Both spp, both str, both sex | *neo* New fems, *try* New fems | no | ✓ | ✓ |  |  | ✓ | ✓ | ✓ | ✓ | ✓ | ✓ |  |  | ✓ | ✓ | ✓ | ✓ | 1 | 1253 | 12 |
| Both spp, both str, both sex | *neo* New fems,*try* Old fems | V *neo* Old fems, V *try* New fems, V *try* New mals |  | ✓ |  | ✓ | ✓ | ✓ |  |  | ✓ | ✓ |  |  | ✓ | ✓ |  | ✓ | 2 | 867, 883 | 9 |
| Both spp, both str, both sex | *neo* New fems,*neo* Old mals | V *try* New fems, V *try* New mals |  | ✓ |  | ✓ | ✓ | ✓ | ✓ | ✓ | ✓ | ✓ |  |  |  |  | ✓ | ✓ | 1 | 981 | 10 |
| Both spp, both str, both sex | *neo* New mals, *neo* Old mals,*try* Old mals | M *neo* New fems | ✓ | ✓ | ✓ | ✓ |  |  | ✓ | ✓ |  |  | ✓ |  |  |  | ✓ | ✓ | 1 | 695 | 9 |
| Both spp, both str, both sex | *neo* Old mals, *try* New mals, *try* Old mals | V *neo* New mals |  |  | ✓ | ✓ |  |  | ✓ | ✓ |  | ✓ | ✓ | ✓ |  |  | ✓ | ✓ | 1 | 1461 | 9 |
| Both spp, both str, both sex | *neo* Old mals,*try* New fems, *try* Old fems | no | ✓ | ✓ |  |  | ✓ | ✓ |  |  | ✓ | ✓ | ✓ | ✓ |  |  | ✓ | ✓ | 1 | 1127 | 10 |
| Both spp, both str, both sex | *neo* Old mals, *try* New mals, *try* Old mals | no |  |  | ✓ | ✓ |  |  | ✓ | ✓ | ✓ | ✓ | ✓ | ✓ |  |  | ✓ | ✓ | 1 | 1083 | 10 |
| Both spp, both str, both sex | *neo* New fems,*try* New fems,*try* Old fems | no | ✓ | ✓ |  |  | ✓ | ✓ |  |  | ✓ | ✓ |  |  | ✓ | ✓ | ✓ | ✓ | 1 | 1094 | 10 |
| Both spp, both str, just mal | - | no | ✓ | ✓ |  |  | ✓ | ✓ |  |  | ✓ | ✓ |  |  | ✓ | ✓ |  |  | 7 | 1164, 1179, 1182, 1187, 1188, 1256, 1468 | 8 |
| Both spp, both str, just mal | - | M *try* Old mals | ✓ | ✓ |  |  | ✓ |  |  |  | ✓ | ✓ |  |  | ✓ | ✓ |  |  | 1 | 1173 | 7 |
| Both spp, both str, just fem | - | no |  |  | ✓ | ✓ |  |  | ✓ | ✓ |  |  | ✓ | ✓ |  |  | ✓ | ✓ | 29 | 1064, 1072, 1174, 1183, 1285, 1292, 1306, 1334, 1340, 1370, 1388, 1434, 1492, 1525, 1592, 1635, 1659, 1675, 1716, 1723, 1788, 1847, 1868, 1889, 1911, 1928, 1981, 1989, 2188 | 8 |
| Both spp, both str, just fem | - | M *try* Old fems |  |  | ✓ | ✓ |  |  | ✓ |  |  |  | ✓ | ✓ |  |  | ✓ | ✓ | 4 | 1059, 1375, 1446, 1499 | 7 |
| Both spp, both str, just mal | neo Old | no | ✓ | ✓ |  |  | ✓ | ✓ |  |  | ✓ | ✓ |  |  |  |  |  |  | 3 | 1115, 1189, 1303 | 6 |
| Both spp, both str, just mal | try New | no |  |  |  |  | ✓ | ✓ |  |  | ✓ | ✓ |  |  | ✓ | ✓ |  |  | 5 | 752, 843, 902, 955, 992 | 6 |
| Both spp, both str, just mal | try New | V *neo* New mals |  |  |  |  | ✓ | ✓ |  |  |  | ✓ |  |  | ✓ | ✓ |  |  | 1 | 933 | 5 |
| Both spp, both str, just mal | neo Old, try New | V *neo* New mals |  |  |  |  |  | ✓ |  |  | ✓ | ✓ |  |  |  |  |  |  | 1 | 702 | 3 |
| Both spp, both str, just fem | neo Old | no |  |  | ✓ | ✓ |  |  | ✓ | ✓ |  |  | ✓ | ✓ |  |  |  |  | 2 | 1185, 1473 | 6 |
| Both spp, just New, just mal | - | M *neo* New mals | ✓ | ✓ |  |  |  |  |  |  | ✓ |  |  |  |  |  |  |  | 1 | 738 | 3 |
| Both spp, just Old, just fem | - | M *neo* Old fems, V *try* Old fems |  |  |  |  |  |  |  | ✓ |  |  |  |  |  |  | ✓ |  | 1 | 1184 | 2 |
| Just neo, just Old, both sex | - | no |  |  |  |  |  |  |  |  |  |  |  |  | ✓ | ✓ | ✓ | ✓ | 1 | 734 | 4 |
| Just try, both str, just mal | - | no | ✓ | ✓ |  |  | ✓ | ✓ |  |  |  |  |  |  |  |  |  |  | 1 | 1123 | 4 |
| Just try, both str, just mal | - | M *try* Old mals | ✓ | ✓ |  |  | ✓ |  |  |  |  |  |  |  |  |  |  |  | 1 | 1171 | 3 |
| Just neo, both str, just mal | - | M *neo* Old mals |  |  |  |  |  |  |  |  | ✓ | ✓ |  |  | ✓ |  |  |  | 1 | 1178 | 3 |
| Just neo, both str, just fem | - | M *neo* New fems |  |  |  |  |  |  |  |  |  |  | ✓ |  |  |  | ✓ | ✓ | 1 | 1191 | 3 |
| Just try, just Old, just mal | - | no |  |  |  |  | ✓ | ✓ |  |  |  |  |  |  |  |  |  |  | 1 | 709 | 2 |
| Just try, just New, just fem | - | no |  |  | ✓ | ✓ |  |  |  |  |  |  |  |  |  |  |  |  | 1 | 1609 | 2 |
| Just try, just Old, just fem | - | no |  |  |  |  |  |  | ✓ | ✓ |  |  |  |  |  |  |  |  | 1 | 1157 | 2 |
| Just neo, just New, just mal | - | no |  |  |  |  |  |  |  |  | ✓ | ✓ |  |  |  |  |  |  | 15 | 723, 795, 799, 831, 951, 962, 989, 993, 1001, 1006, 1087, 1098, 1108, 1124, 1194 | 2 |
| Just neo, just New, just mal | - | V *neo* New mals |  |  |  |  |  |  |  |  |  | ✓ |  |  |  |  |  |  | 1 | 966 | 1 |
| Just neo, just Old, just fem | - | no |  |  |  |  |  |  |  |  |  |  |  |  |  |  | ✓ | ✓ | 3 | 1172, 1327, 1551 | 2 |

**Supplementary Table S2**

| **Significant terms** |  | **Males** |  | **Females** |
| --- | --- | --- | --- | --- |
|  | **Number of peaks** | **Peak identity** | **Number of peaks** | **Peak identity** |
| Sp | 5 | 674, 2Bor, 1173, 1253, 1464 | 8 | 1059, **1127**, **Am2**, 1191, 1473, 1492, PD, **1760** |
| Sp:Dm | 2 | **1127**, DS | 8 | 1185, 1334, 1340, **1357**, 1370, 1499, **E9T**, **E9Hex** |
| Sp:Mh | 1 | 1296 | - |  |
| Dm | 13 | E2MPen, **2E1H**, 1028, MPP, 1115, Bor1, Bor2, 1188, Am5, 1256, 1442, 1454, 1484 | 7 | **2E7MDD**, **Am4**, Am5, 1253, 1267, 1306, **MD** |
| Dm:Mh | - | - | 2 | M9T, **MT** |
| Mh | 1 | Oct-02 | - | - |
| Sp+Dm | 4 | 2M3H, 992, **Am6**, 1468 | - | - |
| Sp+Mh | 2 | 1164, **Am4** | 1 | E9Oct |
| Sp+Sp:Dm | 4 | E2MB, Oct-01, NBCP, Phe | 7 | Am1, **28DDU**, 1375, 1388, 1461, 1484, **ED** |
| Sp+Sp:Dm+Dm | 8 | 3M1B, 795, 799, 2MB, 962, 989, 993, 1001 | 4 | N2MPP, 27DD, 1327, 1609 |
| Sp+Sp:Dm+Mh | - | - | 2 | Oct-02, **EHex** |
| Sp:Dm+Dm | 4 | **E2MP**, 2E4MP, 1189, **Am3** | 4 | 2E4MP, **Am3**, **Am6**, **ET** |
| Sp:Dm+Dm+Mh | 1 | **Am2** | - | - |
| Sp+Sp:Dm+Dm+Mh+Sp:Mh | 1 | 1178 | - | - |
| Sp+Dm+Sp:Dm+Sp:Mh+Dm:Mh | 1 | EP | - | - |
| Dm+Dm:Mh | - | - | 1 | 1464 |
| Dm+Mh | 1 | 4Hep | 2 | Oct1ol, **2E1H** |
| Dm+Mh+Sp:Mh | 2 | 23But meso, **1760** | - | - |
| Sp+Dm:Mh+Mh | 1 | Oct1ol | - | - |
| Sp+Dm+Mh+Sp:Dm+Sp:Mh+Dm:Mh+Sp:Dm:Mh | 8 | 951, 1006, 1083, 1087, 1098, 1108, 1124, 1461 | 1 | 4Hep |

**Supplementary Table S3**

| 1. **Males** | | | | | | | | | | | | | | |
| --- | --- | --- | --- | --- | --- | --- | --- | --- | --- | --- | --- | --- | --- | --- |
| **KI** | **Sp** |  | **Dm** |  | **Mh** |  | **Sp:Dm** |  | **Sp:Mh** |  | **Dm:Mh** |  | **Sp:Dm:Mh** |  |
| 674 | 25.00 | * | 14.58 |  | 1.33 |  | 0.74 |  | 0.43 |  | 0.01 |  | 2.90 |  |
| 695 | 17.35 | . | 17.35 | . | 1.29 |  | 17.35 | . | 1.29 |  | 1.29 |  | 1.29 |  |
| 702 | 40.14 | ** | 40.14 | ** | 0.81 |  | 262.79 | *** | 80.55 | *** | 80.55 | *** | 0.81 |  |
| 709 | 5.20 |  | 5.20 |  | 1.61 |  | 5.20 |  | 1.61 |  | 1.61 |  | 1.61 |  |
| 723 | 39.87 | ** | 39.87 | ** | 0.18 |  | 39.87 | ** | 0.18 |  | 0.18 |  | 0.18 |  |
| 734 | 9.76 |  | 9.76 |  | 2.15 |  | 9.76 |  | 2.15 |  | 2.15 |  | 2.15 |  |
| 738 | 2.54 |  | 16.45 | . | 7.50 |  | 2.54 |  | 8.47 |  | 7.50 |  | 8.47 |  |
| 752 | 0.06 |  | 79.28 | *** | 1.38 |  | 19.52 | * | 2.36 |  | 0.04 |  | 0.30 |  |
| 786 | 3.27 |  | 5.23 |  | 2.07 |  | 3.03 |  | 4.67 |  | 1.77 |  | 0.37 |  |
| 789 | 14.11 |  | 27.25 | ** | 31.33 | ** | 8.59 |  | 43.84 | *** | 0.58 |  | 3.19 |  |
| 795 | 182.69 | *** | 182.69 | *** | 11.73 |  | 182.69 | *** | 11.73 |  | 11.73 |  | 11.73 |  |
| 799 | 30.82 | ** | 30.82 | ** | 1.54 |  | 30.82 | ** | 1.54 |  | 1.54 |  | 1.54 |  |
| 831 | 19.80 | * | 19.80 | * | 11.76 |  | 19.80 | * | 11.76 |  | 11.76 |  | 11.76 |  |
| 843 | 59.98 | *** | 0.01 |  | 2.61 |  | 67.84 | *** | 3.91 |  | 0.30 |  | 0.03 |  |
| 867 | 12.20 |  | 74.40 | *** | 21.24 | * | 15.62 | . | 1.18 |  | 0.02 |  | 1.22 |  |
| 883 | 53.28 | *** | 54.82 | *** | 14.66 |  | 0.28 |  | 0.02 |  | 0.00 |  | 0.03 |  |
| 902 | 45.32 | *** | 0.04 |  | 2.68 |  | 111.41 | *** | 1.55 |  | 1.62 |  | 2.77 |  |
| 933 | 1.95 |  | 27.99 | ** | 0.64 |  | 1.22 |  | 1.65 |  | 1.20 |  | 2.48 |  |
| 939 | 0.81 |  | 3.41 |  | 26.94 | ** | 0.14 |  | 0.78 |  | 1.71 |  | 3.36 |  |
| 951 | 1031.11 | *** | 1031.11 | *** | 324.44 | *** | 1031.11 | *** | 324.44 | *** | 324.44 | *** | 324.44 | *** |
| 955 | 44.02 | *** | 0.03 |  | 2.48 |  | 74.24 | *** | 0.58 |  | 0.98 |  | 3.26 |  |
| 962 | 66.66 | *** | 66.66 | *** | 0.03 |  | 66.66 | *** | 0.03 |  | 0.03 |  | 0.03 |  |
| 966 | 9.68 |  | 9.68 |  | 9.68 |  | 9.68 |  | 9.68 |  | 9.68 |  | 9.68 |  |
| 972 | 79.80 | *** | 0.39 |  | 0.25 |  | 116.19 | *** | 7.50 |  | 5.45 |  | 0.01 |  |
| 981 | 16.22 | . | 24.32 | * | 2.60 |  | 38.68 | ** | 0.33 |  | 2.18 |  | 0.50 |  |
| 989 | 85.75 | *** | 85.75 | *** | 11.73 |  | 85.75 | *** | 11.73 |  | 11.73 |  | 11.73 |  |
| 992 | 29.14 | ** | 23.15 | * | 0.09 |  | 0.27 |  | 0.21 |  | 0.61 |  | 0.39 |  |
| 993 | 151.66 | *** | 151.66 | *** | 6.66 |  | 151.66 | *** | 6.66 |  | 6.66 |  | 6.66 |  |
| 1001 | 79.86 | *** | 79.86 | *** | 0.32 |  | 79.86 | *** | 0.32 |  | 0.32 |  | 0.32 |  |
| 1006 | 164.19 | *** | 164.19 | *** | 26.14 | ** | 164.19 | *** | 26.14 | ** | 26.14 | ** | 26.14 | ** |
| 1019 | 19.66 | * | 1.41 |  | 22.28 | * | 4.09 |  | 6.26 |  | 26.78 | ** | 11.25 |  |
| 1026 | 9.17 |  | 33.62 | ** | 16.25 | . | 10.12 |  | 0.90 |  | 0.01 |  | 0.01 |  |
| 1028 | 6.33 |  | 24.59 | * | 0.11 |  | 2.21 |  | 1.32 |  | 0.02 |  | 1.05 |  |
| 1083 | 151.30 | *** | 151.30 | *** | 79.99 | *** | 151.30 | *** | 79.99 | *** | 79.99 | *** | 79.99 | *** |
| 1087 | 253.70 | *** | 253.70 | *** | 151.78 | *** | 253.70 | *** | 151.78 | *** | 151.78 | *** | 151.78 | *** |
| 1094 | 6.95 |  | 18.84 | * | 0.74 |  | 3.11 |  | 0.79 |  | 0.06 |  | 0.29 |  |
| 1098 | 378.63 | *** | 378.63 | *** | 165.21 | *** | 378.63 | *** | 165.21 | *** | 165.21 | *** | 165.21 | *** |
| 1108 | 1918.40 | *** | 1918.40 | *** | 758.65 | *** | 1918.40 | *** | 758.65 | *** | 758.65 | *** | 758.65 | *** |
| 1115 | 0.90 |  | 26.00 | * | 9.43 |  | 12.15 |  | 0.81 |  | 10.00 |  | 0.65 |  |
| 1123 | 8.82 |  | 5.20 |  | 0.66 |  | 5.20 |  | 0.66 |  | 1.69 |  | 1.69 |  |
| 1124 | 247.81 | *** | 247.81 | *** | 148.07 | *** | 247.81 | *** | 148.07 | *** | 148.07 | *** | 148.07 | *** |
| 1127 | 3.55 |  | 15.66 | . | 1.24 |  | 33.05 | ** | 0.10 |  | 0.38 |  | 0.03 |  |
| 1132 | 13.75 |  | 4.30 |  | 0.71 |  | 4.07 |  | 0.10 |  | 0.15 |  | 0.31 |  |
| 1142 | 3.18 |  | 34.30 | ** | 20.70 | * | 39.50 | ** | 1.38 |  | 1.88 |  | 0.08 |  |
| 1159 | 0.01 |  | 2.50 |  | 14.44 |  | 0.15 |  | 0.05 |  | 0.04 |  | 5.88 |  |
| 1164 | 45.45 | *** | 14.30 |  | 21.83 | * | 1.94 |  | 0.55 |  | 0.02 |  | 0.00 |  |
| 1171 | 25.49 | * | 8.34 |  | 0.61 |  | 8.34 |  | 0.61 |  | 1.91 |  | 1.91 |  |
| 1173 | 21.56 | * | 1.37 |  | 0.23 |  | 0.24 |  | 0.20 |  | 0.02 |  | 0.03 |  |
| 1175 | 2.93 |  | 2.66 |  | 1.95 |  | 0.23 |  | 0.00 |  | 0.75 |  | 0.02 |  |
| 1178 | 288.62 | *** | 83.74 | *** | 40.70 | *** | 83.74 | *** | 40.70 | *** | 2.13 |  | 2.13 |  |
| 1179 | 0.14 |  | 36.19 | ** | 6.31 |  | 0.01 |  | 0.12 |  | 1.02 |  | 0.72 |  |
| 1182 | 0.22 |  | 1.51 |  | 2.58 |  | 31.89 | ** | 0.09 |  | 0.83 |  | 3.68 |  |
| 1187 | 0.21 |  | 32.56 | ** | 6.84 |  | 11.56 |  | 0.79 |  | 0.86 |  | 0.13 |  |
| 1188 | 1.32 |  | 45.39 | *** | 0.30 |  | 2.10 |  | 1.83 |  | 4.31 |  | 0.06 |  |
| 1189 | 1.71 |  | 44.58 | *** | 1.60 |  | 21.22 | * | 0.20 |  | 0.01 |  | 2.69 |  |
| 1194 | 13.53 |  | 13.53 |  | 1.29 |  | 13.53 |  | 1.29 |  | 1.29 |  | 1.29 |  |
| 1201 | 0.25 |  | 28.27 | ** | 9.55 |  | 20.38 | * | 2.90 |  | 2.23 |  | 0.75 |  |
| 1212 | 73.32 | *** | 0.01 |  | 24.95 | * | 4.67 |  | 2.03 |  | 10.25 |  | 1.80 |  |
| 1216 | 0.24 |  | 0.29 |  | 2.49 |  | 3.42 |  | 0.16 |  | 10.29 |  | 6.23 |  |
| 1219 | 3.90 |  | 0.61 |  | 8.77 |  | 0.29 |  | 0.17 |  | 0.49 |  | 0.38 |  |
| 1235 | 4.93 |  | 28.49 | ** | 1.23 |  | 9.23 |  | 2.15 |  | 2.19 |  | 3.23 |  |
| 1239 | 20.65 | * | 19.26 | * | 0.32 |  | 3.20 |  | 3.09 |  | 3.47 |  | 4.02 |  |
| 1244 | 3.97 |  | 14.38 |  | 0.06 |  | 14.18 |  | 2.78 |  | 5.23 |  | 0.01 |  |
| 1253 | 23.46 | * | 8.62 |  | 0.93 |  | 0.22 |  | 0.53 |  | 0.33 |  | 10.40 |  |
| 1256 | 0.00 |  | 77.33 | *** | 15.26 |  | 2.60 |  | 9.56 |  | 0.22 |  | 0.47 |  |
| 1267 | 4.06 |  | 16.65 | . | 2.43 |  | 0.00 |  | 0.02 |  | 1.78 |  | 1.53 |  |
| 1296 | 0.62 |  | 14.05 |  | 8.42 |  | 3.59 |  | 18.63 | * | 0.05 |  | 2.67 |  |
| 1303 | 5.49 |  | 14.25 |  | 8.66 |  | 10.55 |  | 0.81 |  | 5.20 |  | 0.06 |  |
| 1357 | 1.60 |  | 1.42 |  | 16.96 | . | 3.67 |  | 0.12 |  | 17.49 | . | 0.09 |  |
| 1442 | 1.20 |  | 52.02 | *** | 2.25 |  | 9.45 |  | 0.03 |  | 0.28 |  | 0.13 |  |
| 1454 | 0.04 |  | 55.05 | *** | 2.70 |  | 1.20 |  | 0.36 |  | 2.56 |  | 8.34 |  |
| 1461 | 291.34 | *** | 291.34 | *** | 291.34 | *** | 291.34 | *** | 291.34 | *** | 291.34 | *** | 291.34 | *** |
| 1464 | 21.49 | * | 1.21 |  | 2.55 |  | 7.95 |  | 0.12 |  | 0.82 |  | 1.89 |  |
| 1468 | 26.16 | ** | 22.59 | * | 8.86 |  | 1.43 |  | 0.25 |  | 0.14 |  | 0.85 |  |
| 1484 | 5.37 |  | 55.86 | *** | 1.73 |  | 0.16 |  | 0.15 |  | 2.36 |  | 4.35 |  |
| 1583 | 0.00 |  | 4.88 |  | 5.11 |  | 3.97 |  | 1.04 |  | 0.06 |  | 1.01 |  |
| 1760 | 11.25 |  | 54.48 | *** | 75.28 | *** | 10.83 |  | 75.74 | *** | 4.13 |  | 4.24 |  |
| 1783 | 0.00 |  | 3.09 |  | 6.49 |  | 5.32 |  | 2.30 |  | 0.06 |  | 0.62 |  |
| 1. **Females** | | | | | | | | | | | | | |  |
| 674 | 0.29 |  | 10.00 |  | 0.15 |  | 8.95 |  | 4.78 |  | 6.04 |  | 0.16 |  |
| 695 | 4.87 |  | 0.79 |  | 1.87 |  | 1.93 |  | 3.66 |  | 0.88 |  | 0.03 |  |
| 734 | 5.48 |  | 5.48 |  | 0.05 |  | 5.48 |  | 0.05 |  | 0.05 |  | 0.05 |  |
| 786 | 6.28 |  | 6.60 |  | 3.24 |  | 0.26 |  | 9.01 |  | 0.74 |  | 4.26 |  |
| 789 | 4.93 |  | 0.68 |  | 0.96 |  | 0.25 |  | 4.71 |  | 1.53 |  | 1.32 |  |
| 867 | 77.41 | *** | 77.41 | *** | 129.20 | *** | 129.20 | *** | 77.41 | *** | 77.41 | *** | 129.20 | *** |
| 883 | 7.05 |  | 7.05 |  | 12.42 |  | 12.42 |  | 7.05 |  | 7.05 |  | 12.42 |  |
| 939 | 19.47 | * | 5.04 |  | 52.40 | *** | 42.84 | *** | 5.35 |  | 0.73 |  | 1.41 |  |
| 972 | 0.44 |  | 10.64 |  | 0.00 |  | 0.18 |  | 8.39 |  | 0.09 |  | 9.79 |  |
| 981 | 4.05 |  | 46.78 | *** | 4.87 |  | 19.65 | * | 0.06 |  | 0.05 |  | 7.06 |  |
| 1019 | 6.00 |  | 19.93 | * | 20.53 | * | 2.27 |  | 0.15 |  | 0.04 |  | 1.87 |  |
| 1026 | 1.82 |  | 18.84 | * | 66.68 | *** | 0.70 |  | 0.35 |  | 0.19 |  | 9.92 |  |
| 1028 | 5.07 |  | 6.05 |  | 7.36 |  | 3.80 |  | 0.82 |  | 0.07 |  | 5.15 |  |
| 1059 | 33.90 | ** | 4.44 |  | 3.66 |  | 8.25 |  | 1.72 |  | 3.93 |  | 0.77 |  |
| 1064 | 0.27 |  | 10.74 |  | 0.32 |  | 2.79 |  | 1.55 |  | 1.12 |  | 0.07 |  |
| 1072 | 10.33 |  | 9.71 |  | 7.27 |  | 13.69 |  | 0.93 |  | 0.01 |  | 2.93 |  |
| 1083 | 3.57 |  | 8.28 |  | 1.37 |  | 6.34 |  | 0.01 |  | 1.89 |  | 0.10 |  |
| 1094 | 39.41 | *** | 39.41 | *** | 0.03 |  | 39.41 | *** | 0.03 |  | 0.03 |  | 0.03 |  |
| 1127 | 64.27 | *** | 12.31 |  | 4.79 |  | 12.31 |  | 4.79 |  | 0.00 |  | 0.00 |  |
| 1132 | 48.19 | *** | 0.06 |  | 1.09 |  | 41.85 | *** | 0.09 |  | 4.71 |  | 0.96 |  |
| 1142 | 44.26 | *** | 0.80 |  | 2.74 |  | 12.44 |  | 0.11 |  | 0.52 |  | 2.61 |  |
| 1157 | 18.02 | . | 18.02 | . | 0.95 |  | 18.02 | . | 0.95 |  | 0.95 |  | 0.95 |  |
| 1159 | 18.74 | * | 4.01 |  | 2.43 |  | 64.97 | *** | 0.59 |  | 0.59 |  | 18.01 | . |
| 1172 | 55.04 | *** | 55.04 | *** | 0.19 |  | 55.04 | *** | 0.19 |  | 0.19 |  | 0.19 |  |
| 1174 | 3.33 |  | 45.73 | *** | 1.40 |  | 0.36 |  | 1.79 |  | 0.21 |  | 0.99 |  |
| 1175 | 0.34 |  | 10.77 |  | 0.01 |  | 0.46 |  | 1.74 |  | 0.32 |  | 0.83 |  |
| 1183 | 1.44 |  | 13.50 |  | 0.03 |  | 1.09 |  | 1.59 |  | 4.55 |  | 0.00 |  |
| 1184 | 0.00 |  | 14.57 |  | 0.00 |  | 0.00 |  | 14.57 |  | 0.00 |  | 14.57 |  |
| 1185 | 15.27 |  | 2.63 |  | 4.99 |  | 23.94 | * | 0.06 |  | 1.91 |  | 1.20 |  |
| 1191 | 22.18 | * | 11.02 |  | 0.01 |  | 11.02 |  | 0.01 |  | 2.19 |  | 2.19 |  |
| 1201 | 2.27 |  | 20.63 | * | 8.31 |  | 38.20 | ** | 0.41 |  | 5.80 |  | 2.14 |  |
| 1212 | 3.47 |  | 25.45 | ** | 5.51 |  | 13.13 |  | 0.08 |  | 5.91 |  | 2.83 |  |
| 1216 | 0.33 |  | 15.74 | . | 0.57 |  | 6.17 |  | 0.57 |  | 0.42 |  | 9.50 |  |
| 1219 | 1.09 |  | 11.15 |  | 0.70 |  | 18.00 | . | 3.79 |  | 0.00 |  | 0.12 |  |
| 1235 | 8.55 |  | 20.44 | * | 0.02 |  | 10.11 |  | 1.50 |  | 11.04 |  | 0.65 |  |
| 1239 | 7.39 |  | 26.06 | ** | 12.40 |  | 34.98 | ** | 0.01 |  | 2.95 |  | 14.42 |  |
| 1244 | 1.49 |  | 7.12 |  | 0.03 |  | 0.02 |  | 1.62 |  | 0.36 |  | 0.68 |  |
| 1253 | 1.29 |  | 38.04 | ** | 0.02 |  | 1.29 |  | 0.01 |  | 0.02 |  | 0.01 |  |
| 1267 | 7.30 |  | 30.94 | ** | 0.66 |  | 0.01 |  | 0.20 |  | 0.00 |  | 0.17 |  |
| 1285 | 4.20 |  | 6.18 |  | 0.47 |  | 2.74 |  | 2.95 |  | 0.06 |  | 0.00 |  |
| 1292 | 0.00 |  | 0.07 |  | 10.62 |  | 3.53 |  | 0.28 |  | 2.22 |  | 2.56 |  |
| 1296 | 8.68 |  | 5.38 |  | 0.17 |  | 12.75 |  | 0.01 |  | 1.04 |  | 0.32 |  |
| 1306 | 5.93 |  | 44.15 | *** | 5.57 |  | 0.25 |  | 1.30 |  | 0.58 |  | 0.14 |  |
| 1327 | 26.11 | ** | 26.11 | ** | 5.32 |  | 26.11 | ** | 5.32 |  | 5.32 |  | 5.32 |  |
| 1334 | 3.18 |  | 9.68 |  | 3.66 |  | 31.81 | ** | 0.83 |  | 0.05 |  | 1.94 |  |
| 1340 | 4.88 |  | 6.79 |  | 3.49 |  | 25.72 | ** | 0.45 |  | 0.11 |  | 1.71 |  |
| 1357 | 12.72 |  | 7.48 |  | 9.53 |  | 39.71 | *** | 0.71 |  | 4.42 |  | 5.55 |  |
| 1370 | 7.34 |  | 4.01 |  | 0.73 |  | 26.26 | ** | 0.14 |  | 0.45 |  | 0.00 |  |
| 1375 | 35.14 | ** | 1.10 |  | 4.77 |  | 32.83 | ** | 3.28 |  | 0.01 |  | 1.57 |  |
| 1388 | 20.42 | * | 0.40 |  | 1.52 |  | 25.37 | ** | 0.53 |  | 0.14 |  | 2.85 |  |
| 1434 | 0.00 |  | 1.91 |  | 2.56 |  | 9.59 |  | 0.76 |  | 0.00 |  | 0.45 |  |
| 1442 | 0.92 |  | 0.12 |  | 5.38 |  | 1.20 |  | 1.43 |  | 0.13 |  | 13.14 |  |
| 1446 | 2.76 |  | 0.41 |  | 3.49 |  | 2.96 |  | 3.99 |  | 0.57 |  | 5.33 |  |
| 1454 | 11.57 |  | 1.88 |  | 5.46 |  | 4.54 |  | 1.19 |  | 1.32 |  | 6.52 |  |
| 1461 | 26.13 | ** | 2.82 |  | 11.04 |  | 28.32 | ** | 1.17 |  | 0.60 |  | 2.69 |  |
| 1464 | 9.06 |  | 121.07 | *** | 2.76 |  | 0.00 |  | 3.95 |  | 21.87 | * | 1.05 |  |
| 1473 | 42.60 | *** | 0.30 |  | 2.38 |  | 8.95 |  | 1.28 |  | 0.05 |  | 0.40 |  |
| 1484 | 18.82 | * | 4.69 |  | 0.21 |  | 23.51 | * | 7.93 |  | 1.24 |  | 2.00 |  |
| 1492 | 23.29 | * | 4.95 |  | 2.61 |  | 5.53 |  | 0.32 |  | 0.48 |  | 0.31 |  |
| 1499 | 8.99 |  | 7.85 |  | 0.97 |  | 21.58 | * | 4.99 |  | 0.34 |  | 2.40 |  |
| 1525 | 5.54 |  | 22.71 | * | 6.32 |  | 0.48 |  | 0.30 |  | 3.59 |  | 7.98 |  |
| 1551 | 14.96 |  | 14.96 |  | 3.33 |  | 14.96 |  | 3.33 |  | 3.33 |  | 3.33 |  |
| 1583 | 0.37 |  | 7.62 |  | 0.46 |  | 1.03 |  | 0.38 |  | 1.39 |  | 0.82 |  |
| 1592 | 30.98 | ** | 7.07 |  | 2.82 |  | 18.70 | * | 3.02 |  | 3.40 |  | 13.27 |  |
| 1609 | 21.88 | * | 21.88 | * | 8.51 |  | 21.88 | * | 8.51 |  | 8.51 |  | 8.51 |  |
| 1635 | 0.01 |  | 1.14 |  | 0.33 |  | 2.40 |  | 4.70 |  | 4.90 |  | 5.01 |  |
| 1659 | 11.45 |  | 1.39 |  | 0.00 |  | 1.71 |  | 8.78 |  | 0.06 |  | 0.02 |  |
| 1675 | 31.73 | ** | 1.65 |  | 1.81 |  | 9.89 |  | 4.81 |  | 1.16 |  | 1.29 |  |
| 1716 | 5.62 |  | 11.81 |  | 0.14 |  | 14.65 |  | 3.97 |  | 29.82 | ** | 9.55 |  |
| 1723 | 14.54 |  | 1.69 |  | 0.24 |  | 11.99 |  | 0.61 |  | 24.43 | * | 0.34 |  |
| 1760 | 59.72 | *** | 5.87 |  | 0.57 |  | 7.26 |  | 0.32 |  | 3.47 |  | 6.62 |  |
| 1783 | 0.10 |  | 5.39 |  | 0.22 |  | 36.72 | ** | 0.22 |  | 2.86 |  | 4.88 |  |
| 1788 | 4.18 |  | 25.24 | * | 5.26 |  | 21.54 | * | 2.04 |  | 0.15 |  | 8.56 |  |
| 1847 | 0.16 |  | 1.94 |  | 5.33 |  | 2.55 |  | 8.90 |  | 9.73 |  | 7.69 |  |
| 1868 | 0.08 |  | 0.23 |  | 0.06 |  | 5.78 |  | 9.49 |  | 0.30 |  | 0.83 |  |
| 1889 | 3.65 |  | 1.98 |  | 2.35 |  | 1.19 |  | 8.49 |  | 2.36 |  | 0.72 |  |
| 1911 | 1.83 |  | 12.36 |  | 0.64 |  | 12.97 |  | 0.54 |  | 9.60 |  | 0.29 |  |
| 1928 | 9.31 |  | 0.71 |  | 2.65 |  | 7.59 |  | 0.12 |  | 8.29 |  | 0.06 |  |
| 1981 | 2.83 |  | 8.47 |  | 0.55 |  | 34.00 | ** | 0.99 |  | 1.32 |  | 6.72 |  |
| 1989 | 54.19 | *** | 15.41 | . | 42.74 | *** | 26.66 | ** | 3.05 |  | 2.69 |  | 3.00 |  |
| 2188 | 26.65 | ** | 10.65 |  | 20.61 | * | 0.01 |  | 1.32 |  | 10.80 |  | 0.10 |  |

**Supplementary Table S4**

| **Term** | **Category** | **674** | **702** | **723** | **752** | **789** | **795** | **799** | | **831** | **843** | |
| --- | --- | --- | --- | --- | --- | --- | --- | --- | --- | --- | --- | --- |
| Sp | *try* | 0.0158 (0.0128,0.0227) | - | - | - | - | - | - | | - | - | |
|  | *neo* | 0.0011 (0.0002,0.0099) | - | - | - | - | - | - | | - | - | |
| Dm | New | - | - | - | - | 0.0017 (0.0008,0.0034) | - | - | | - | - | |
|  | Old | - | - | - | - | 0.0000 (0.0000,0.0001) | - | - | | - | - | |
| Mh | Virgin male | - | - | - | - | - | - | - | | - | - | |
|  | Mixed male | - | - | - | - | - | - | - | | - | - | |
| Sp:Dm | *try* New | - | 0.0000 (0.0000,0.0001) | 0.0000 (0.0000,0.0019) | 0.0000 (0.0000,0.1450) | - | 0.0000 (0.0000,0.0011) | 0.0000 (0.0000,0.0005) | | 0.0000 (0.0000,0.0628) | 0.0000 (0.0000,0.0354) | |
|  | *neo* New | - | 0.0058 (0.0029,0.0065) | 0.0131 (0.0110,0.0152) | 0.4273 (0.1871,0.8054) | - | 0.0085 (0.0072,0.0097) | 0.0024 (0.0019,0.0030) | | 0.2131 (0.1248,0.3165) | 0.5951 (0.5052,0.6496) | |
|  | *try* Old | - | 0.0004 (0.0003,0.0005) | 0.0000 (0.0000,0.0019) | 2.4988 (2.2817,3.2597) | - | 0.0000 (0.0000,0.0011) | 0.0000 (0.0000,0.0005) | | 0.0000 (0.0000,0.0628) | 0.1417 (0.1279,0.1947) | |
|  | *neo* Old | - | 0.0000 (0.0000,0.0001) | 0.0000 (0.0000,0.0019) | 1.2333 (0.8214,2.2613) | - | 0.0000 (0.0000,0.0011) | 0.0000 (0.0000,0.0005) | | 0.0000 (0.0000,0.0628) | 0.1407 (0.1217,0.1839) | |
| Sp:Mh | *try* Mixed male | - | 0.0004 (0.0003,0.0005) | - | - | 0.0108 (0.0067,0.0139) | - | - | | - | - | |
|  | *neo* Mixed male | - | 0.0003 (0.0002,0.0004) | - | - | 0.0000 (0.0000,0.0005) | - | - | | - | - | |
|  | *try* Virgin male | - | 0.0000 (0.0000,0.0001) | - | - | 0.0000 (0.0000,0.0000) | - | - | | - | - | |
|  | *neo* Virgin male | - | 0.0014 (0.0005,0.0020) | - | - | 0.0000 (0.0000,0.0011) | - | - | | - | - | |
| Dm:Mh | New Mixed male | - | 0.0003 (0.0002,0.0004) | - | - | - | - | - | | - | - | |
|  | Old Mixed male | - | 0.0004 (0.0003,0.0005) | - | - | - | - | - | | - | - | |
|  | New Virgin male | - | 0.0014 (0.0005,0.0020) | - | - | - | - | - | | - | - | |
|  | Old Virgin male | - | 0.0000 (0.0000,0.0001) | - | - | - | - | - | | - | - | |
| Sp:Dm:Mh | *try* New Mixed male | - | - | - | - | - | - | - | | - | - | |
|  | *neo* New Mixed male | - | - | - | - | - | - | - | | - | - | |
|  | *try* Old Mixed male | - | - | - | - | - | - | - | | - | - | |
|  | *neo* Old Mixed male | - | - | - | - | - | - | - | | - | - | |
|  | *try* New Virgin male | - | - | - | - | - | - | - | | - | - | |
|  | *neo* New Virgin male | - | - | - | - | - | - | - | | - | - | |
|  | *try* Old Virgin male | - | - | - | - | - | - | - | | - | - | |
|  | *neo* Old Virgin male | - | - | - | - | - | - | - | - | | - |  |

| **Term** | **Category** | **867** | **883** | **902** | **933** | **939** | **951** | **955** | **962** | **972** |
| --- | --- | --- | --- | --- | --- | --- | --- | --- | --- | --- |
| Sp | *try* | - | 0.0121 (0.0055,0.0205) | - | - | - | - | - | - | - |
|  | *neo* | - | 0.0746 (0.0445,0.1112) | - | - | - | - | - | - | - |
| Dm | New | 0.0008 (0.0002,0.0018) | 0.0113 (0.0047,0.0189) | - | 0.0001 (0.0000,0.0014) | - | - | - | - | - |
|  | Old | 0.0161 (0.0070,0.0199) | 0.0829 (0.0448,0.1117) | - | 0.0045 (0.0032,0.0057) | - | - | - | - | - |
| Mh | Virgin male | 0.0016 (0.0008,0.0028) | - | - | - | 0.0071 (0.0038,0.0120) | - | - | - | - |
|  | Mixed male | 0.0075 (0.0037,0.0169) | - | - | - | 0.0961 (0.0661,0.1845) | - | - | - | - |
| Sp:Dm | *try* New | - | - | 0.0000 (0.0000,0.0260) | - | - | - | 0.0000 (0.0000,0.0004) | 0.0000 (0.0000,0.0002) | 0.0000 (0.0000,0.0034) |
|  | *neo* New | - | - | 0.6046 (0.5116,0.7163) | - | - | - | 0.0403 (0.0289,0.0558) | 0.0044 (0.0030,0.0068) | 0.0785 (0.0665,0.0920) |
|  | *try* Old | - | - | 0.2163 (0.1062,0.3984) | - | - | - | 0.0053 (0.0035,0.0155) | 0.0000 (0.0000,0.0002) | 0.0099 (0.0081,0.0170) |
|  | *neo* Old | - | - | 0.0817 (0.0558,0.1128) | - | - | - | 0.0036 (0.0011,0.0056) | 0.0000 (0.0000,0.0002) | 0.0086 (0.0078,0.0101) |
| Sp:Mh | *try* Mixed male | - | - | - | - | - | - | - | - | - |
|  | *neo* Mixed male | - | - | - | - | - | - | - | - | - |
|  | *try* Virgin male | - | - | - | - | - | - | - | - | - |
|  | *neo* Virgin male | - | - | - | - | - | - | - | - | - |
| Dm:Mh | New Mixed male | - | - | - | - | - | - | - | - | - |
|  | Old Mixed male | - | - | - | - | - | - | - | - | - |
|  | New Virgin male | - | - | - | - | - | - | - | - | - |
|  | Old Virgin male | - | - | - | - | - | - | - | - | - |
| Sp:Dm:Mh | *try* New Mixed male | - | - | - | - | - | 0.0000 (0.0000,0.0002) | - | - | - |
|  | *neo* New Mixed male | - | - | - | - | - | 0.0070 (0.0068,0.0073) | - | - | - |
|  | *try* Old Mixed male | - | - | - | - | - | 0.0000 (0.0000,0.0002) | - | - | - |
|  | *neo* Old Mixed male | - | - | - | - | - | 0.0000 (0.0000,0.0002) | - | - | - |
|  | *try* New Virgin male | - | - | - | - | - | 0.0000 (0.0000,0.0002) | - | - | - |
|  | *neo* New Virgin male | - | - | - | - | - | 0.0020 (0.0018,0.0022) | - | - | - |
|  | *try* Old Virgin male | - | - | - | - | - | 0.0000 (0.0000,0.0002) | - | - | - |
|  | *neo* Old Virgin male | - | - | - | - | - | 0.0000 (0.0000,0.0002) | - | - | - |

| **Term** | **Category** | **981** | **989** | **992** | **993** | **1001** | **1006** | **1019** | **1026** | **1028** |
| --- | --- | --- | --- | --- | --- | --- | --- | --- | --- | --- |
| Sp | *try* | - | - | 0.0009 (0.0004,0.0033) | - | - | - | 0.0042 (0.0013,0.0092) | - | - |
|  | *neo* | - | - | 0.0064 (0.0058,0.0075) | - | - | - | 0.0158 (0.0104,0.0178) | - | - |
| Dm | New | - | - | 0.0010 (0.0005,0.0035) | - | - | - | - | 3.8974 (0.8588,10.8748) | 0.0377 (0.0231,0.0638) |
|  | Old | - | - | 0.0063 (0.0057,0.0072) | - | - | - | - | 0.3468 (0.2572,0.6677) | 0.0139 (0.0112,0.0180) |
| Mh | Virgin male | - | - | - | - | - | - | - | - | - |
|  | Mixed male | - | - | - | - | - | - | - | - | - |
| Sp:Dm | *try* New | 0.0001 (0.0000,0.0004) | 0.0000 (0.0000,0.0020) | - | 0.0000 (0.0000,0.0002) | 0.0000 (0.0000,0.0015) | - | - | - | - |
|  | *neo* New | 0.0073 (0.0044,0.0112) | 0.0100 (0.0078,0.0123) | - | 0.0063 (0.0046,0.0074) | 0.0143 (0.0126,0.0159) | - | - | - | - |
|  | *try* Old | 0.0003 (0.0000,0.0005) | 0.0000 (0.0000,0.0020) | - | 0.0000 (0.0000,0.0002) | 0.0000 (0.0000,0.0015) | - | - | - | - |
|  | *neo* Old | 0.0000 (0.0000,0.0002) | 0.0000 (0.0000,0.0020) | - | 0.0000 (0.0000,0.0002) | 0.0000 (0.0000,0.0015) | - | - | - | - |
| Sp:Mh | *try* Mixed male | - | - | - | - | - | - | - | - | - |
|  | *neo* Mixed male | - | - | - | - | - | - | - | - | - |
|  | *try* Virgin male | - | - | - | - | - | - | - | - | - |
|  | *neo* Virgin male | - | - | - | - | - | - | - | - | - |
| Dm:Mh | New Mixed male | - | - | - | - | - | - | 0.0198 (0.0163,0.0247) | - | - |
|  | Old Mixed male | - | - | - | - | - | - | 0.0090 (0.0026,0.0145) | - | - |
|  | New Virgin male | - | - | - | - | - | - | 0.0013 (0.0006,0.0078) | - | - |
|  | Old Virgin male | - | - | - | - | - | - | 0.0091 (0.0035,0.0155) | - | - |
| Sp:Dm:Mh | *try* New Mixed male | - | - | - | - | - | 0.0000 (0.0000,0.0003) | - | - | - |
|  | *neo* New Mixed male | - | - | - | - | - | 0.0018 (0.0014,0.0022) | - | - | - |
|  | *try* Old Mixed male | - | - | - | - | - | 0.0000 (0.0000,0.0003) | - | - | - |
|  | *neo* Old Mixed male | - | - | - | - | - | 0.0000 (0.0000,0.0003) | - | - | - |
|  | *try* New Virgin male | - | - | - | - | - | 0.0000 (0.0000,0.0003) | - | - | - |
|  | *neo* New Virgin male | - | - | - | - | - | 0.0042 (0.0039,0.0045) | - | - | - |
|  | *try* Old Virgin male | - | - | - | - | - | 0.0000 (0.0000,0.0003) | - | - | - |
|  | *neo* Old Virgin male | - | - | - | - | - | 0.0000 (0.0000,0.0003) | - | - | - |

| **Term** | **Category** | **1083** | **1087** | **1094** | **1098** | **1108** | **1115** | **1124** | **1127** | **1142** |
| --- | --- | --- | --- | --- | --- | --- | --- | --- | --- | --- |
| Sp | *try* | - | - | - | - | - | - | - | - | - |
|  | *neo* | - | - | - | - | - | - | - | - | - |
| Dm | New | - | - | 0.0192 (0.0137,0.0290) | - | - | 0.0377 (0.0239,0.0512) | - | - | - |
|  | Old | - | - | 0.0679 (0.0482,0.1025) | - | - | 0.0084 (0.0000,0.0217) | - | - | - |
| Mh | Virgin male | - | - | - | - | - | - | - | - | 5.5666 (5.4993,6.1173) |
|  | Mixed male | - | - | - | - | - | - | - | - | 7.7079 (6.8083,10.0463) |
| Sp:Dm | *try* New | - | - | - | - | - | - | - | 0.0010 (0.0001,0.0033) | 6.2689 (5.5461,7.5091) |
|  | *neo* New | - | - | - | - | - | - | - | 10.2593 (8.1229,11.5999) | 4.3375 (3.6345,5.5273) |
|  | *try* Old | - | - | - | - | - | - | - | 0.0030 (0.0009,0.0151) | 5.8412 (5.5313,7.1196) |
|  | *neo* Old | - | - | - | - | - | - | - | 0.0000 (0.0000,0.0009) | 19.9276 (13.2212,20.2612) |
| Sp:Mh | *try* Mixed male | - | - | - | - | - | - | - | - | - |
|  | *neo* Mixed male | - | - | - | - | - | - | - | - | - |
|  | *try* Virgin male | - | - | - | - | - | - | - | - | - |
|  | *neo* Virgin male | - | - | - | - | - | - | - | - | - |
| Dm:Mh | New Mixed male | - | - | - | - | - | - | - | - | - |
|  | Old Mixed male | - | - | - | - | - | - | - | - | - |
|  | New Virgin male | - | - | - | - | - | - | - | - | - |
|  | Old Virgin male | - | - | - | - | - | - | - | - | - |
| Sp:Dm:Mh | *try* New Mixed male | 0.0000 (0.0000,0.0049) | 0.0000 (0.0000,0.0040) | - | 0.0000 (0.0000,0.0033) | 0.0000 (0.0000,0.0008) | - | 0.0000 (0.0000,0.0044) | - | - |
|  | *neo* New Mixed male | 0.0728 (0.0666,0.0791) | 0.0795 (0.0744,0.0846) | - | 0.0737 (0.0697,0.0778) | 0.0389 (0.0379,0.0399) | - | 0.0855 (0.0799,0.0911) | - | - |
|  | *try* Old Mixed male | 0.0000 (0.0000,0.0049) | 0.0000 (0.0000,0.0040) | - | 0.0000 (0.0000,0.0033) | 0.0000 (0.0000,0.0008) | - | 0.0000 (0.0000,0.0044) | - | - |
|  | *neo* Old Mixed male | 0.0000 (0.0000,0.0049) | 0.0000 (0.0000,0.0040) | - | 0.0000 (0.0000,0.0033) | 0.0000 (0.0000,0.0008) | - | 0.0000 (0.0000,0.0044) | - | - |
|  | *try* New Virgin male | 0.0000 (0.0000,0.0049) | 0.0000 (0.0000,0.0040) | - | 0.0000 (0.0000,0.0033) | 0.0000 (0.0000,0.0008) | - | 0.0000 (0.0000,0.0044) | - | - |
|  | *neo* New Virgin male | 0.0113 (0.0064,0.0163) | 0.0100 (0.0059,0.0140) | - | 0.0151 (0.0117,0.0184) | 0.0085 (0.0077,0.0092) | - | 0.0107 (0.0063,0.0152) | - | - |
|  | *try* Old Virgin male | 0.0000 (0.0000,0.0049) | 0.0000 (0.0000,0.0040) | - | 0.0000 (0.0000,0.0033) | 0.0000 (0.0000,0.0008) | - | 0.0000 (0.0000,0.0044) | - | - |
|  | *neo* Old Virgin male | 0.0000 (0.0000,0.0049) | 0.0000 (0.0000,0.0040) | - | 0.0000 (0.0000,0.0033) | 0.0000 (0.0000,0.0008) | - | 0.0000 (0.0000,0.0044) | - | - |

| **Term** | **Category** | **1164** | **1171** | **1173** | **1178** | **1179** | **1182** | **1187** | **1188** | **1189** |
| --- | --- | --- | --- | --- | --- | --- | --- | --- | --- | --- |
| Sp | *try* | 0.0915 (0.0765,0.1365) | 0.0277 (0.0171,0.0383) | 0.0013 (0.0000,0.0040) | - | - | - | - | - | - |
|  | *neo* | 0.3284 (0.2033,0.3984) | 0.0000 (0.0000,0.0109) | 0.0108 (0.0080,0.0135) | - | - | - | - | - | - |
| Dm | New | - | - | - | - | 0.0511 (0.0384,0.0520) | - | 0.0488 (0.0328,0.0537) | 0.1900 (0.1375,0.2018) | - |
|  | Old | - | - | - | - | 0.0132 (0.0105,0.0175) | - | 0.0047 (0.0037,0.0155) | 0.0139 (0.0121,0.0272) | - |
| Mh | Virgin male | 0.2708 (0.1720,0.3878) | - | - | - | - | - | - | - | - |
|  | Mixed male | 0.0973 (0.0838,0.1622) | - | - | - | - | - | - | - | - |
| Sp:Dm | *try* New | - | - | - | 0.0000 (0.0000,0.0001) | - | 0.0364 (0.0250,0.0496) | - | - | 0.0181 (0.0069,0.0378) |
|  | *neo* New | - | - | - | 0.0135 (0.0132,0.0159) | - | 0.1061 (0.0905,0.1519) | - | - | 0.1795 (0.1467,0.1960) |
|  | *try* Old | - | - | - | 0.0000 (0.0000,0.0001) | - | 0.1140 (0.0952,0.1862) | - | - | 0.0069 (0.0026,0.0181) |
|  | *neo* Old | - | - | - | 0.0002 (0.0001,0.0002) | - | 0.0471 (0.0336,0.0906) | - | - | 0.0000 (0.0000,0.0043) |
| Sp:Mh | *try* Mixed male | - | - | - | 0.0000 (0.0000,0.0001) | - | - | - | - | - |
|  | *neo* Mixed male | - | - | - | 0.0003 (0.0002,0.0003) | - | - | - | - | - |
|  | *try* Virgin male | - | - | - | 0.0000 (0.0000,0.0001) | - | - | - | - | - |
|  | *neo* Virgin male | - | - | - | 0.0132 (0.0090,0.0134) | - | - | - | - | - |
| Dm:Mh | New Mixed male | - | - | - | - | - | - | - | - | - |
|  | Old Mixed male | - | - | - | - | - | - | - | - | - |
|  | New Virgin male | - | - | - | - | - | - | - | - | - |
|  | Old Virgin male | - | - | - | - | - | - | - | - | - |
| Sp:Dm:Mh | *try* New Mixed male | - | - | - | - | - | - | - | - | - |
|  | *neo* New Mixed male | - | - | - | - | - | - | - | - | - |
|  | *try* Old Mixed male | - | - | - | - | - | - | - | - | - |
|  | *neo* Old Mixed male | - | - | - | - | - | - | - | - | - |
|  | *try* New Virgin male | - | - | - | - | - | - | - | - | - |
|  | *neo* New Virgin male | - | - | - | - | - | - | - | - | - |
|  | *try* Old Virgin male | - | - | - | - | - | - | - | - | - |
|  | *neo* Old Virgin male | - | - | - | - | - | - | - | - | - |

| **Term** | **Category** | **1201** | **1212** | **1235** | **1239** | **1253** | **1256** | **1296** | **1442** | **1454** |
| --- | --- | --- | --- | --- | --- | --- | --- | --- | --- | --- |
| Sp | *try* | - | 76.7953 (72.4428,77.7919) | - | 8.7096 (8.2324,10.6089) | 0.0237 (0.0174,0.0334) | - | - | - | - |
|  | *neo* | - | 64.7106 (63.3770,65.4629) | - | 3.5745 (0.8663,7.5768) | 0.0591 (0.0468,0.0734) | - | - | - | - |
| Dm | New | - | - | 0.1632 (0.0601,0.2863) | 3.6357 (0.8800,7.6791) | - | 0.1621 (0.1085,0.1706) | - | 0.1041 (0.0467,0.1386) | 0.0970 (0.0808,0.1046) |
|  | Old | - | - | 0.4314 (0.3189,0.5391) | 8.6831 (8.1408,10.5384) | - | 0.0564 (0.0461,0.0629) | - | 0.0111 (0.0088,0.0134) | 0.0287 (0.0222,0.0361) |
| Mh | Virgin male | - | 72.5087 (70.2582,77.1907) | - | - | - | - | - | - | - |
|  | Mixed male | - | 65.3106 (64.6913,69.1845) | - | - | - | - | - | - | - |
| Sp:Dm | *try* New | 2.4436 (2.1713,3.1873) | - | - | - | - | - | - | - | - |
|  | *neo* New | 2.0085 (1.5108,2.4181) | - | - | - | - | - | - | - | - |
|  | *try* Old | 2.5352 (2.2587,3.4650) | - | - | - | - | - | - | - | - |
|  | *neo* Old | 4.1731 (3.4581,5.0506) | - | - | - | - | - | - | - | - |
| Sp:Mh | *try* Mixed male | - | - | - | - | - | - | 0.0154 (0.0081,0.0179) | - | - |
|  | *neo* Mixed male | - | - | - | - | - | - | 0.0030 (0.0000,0.0089) | - | - |
|  | *try* Virgin male | - | - | - | - | - | - | 0.0000 (0.0000,0.0050) | - | - |
|  | *neo* Virgin male | - | - | - | - | - | - | 0.0063 (0.0013,0.0139) | - | - |
| Dm:Mh | New Mixed male | - | - | - | - | - | - | - | - | - |
|  | Old Mixed male | - | - | - | - | - | - | - | - | - |
|  | New Virgin male | - | - | - | - | - | - | - | - | - |
|  | Old Virgin male | - | - | - | - | - | - | - | - | - |
| Sp:Dm:Mh | *try* New Mixed male | - | - | - | - | - | - | - | - | - |
|  | *neo* New Mixed male | - | - | - | - | - | - | - | - | - |
|  | *try* Old Mixed male | - | - | - | - | - | - | - | - | - |
|  | *neo* Old Mixed male | - | - | - | - | - | - | - | - | - |
|  | *try* New Virgin male | - | - | - | - | - | - | - | - | - |
|  | *neo* New Virgin male | - | - | - | - | - | - | - | - | - |
|  | *try* Old Virgin male | - | - | - | - | - | - | - | - | - |
|  | *neo* Old Virgin male | - | - | - | - | - | - | - | - | - |

| **Term** | **Category** | **1461** | **1464** | **1468** | **1484** | **1760** |
| --- | --- | --- | --- | --- | --- | --- |
| Sp | *try* | - | 0.1775 (0.1138,0.2179) | 0.0081 (0.0073,0.0149) | - | - |
|  | *neo* | - | 0.0706 (0.0496,0.0897) | 0.0404 (0.0305,0.0667) | - | - |
| Dm | New | - | - | 0.0081 (0.0070,0.0152) | 0.3270 (0.2324,0.3720) | 0.0019 (0.0007,0.0031) |
|  | Old | - | - | 0.0403 (0.0314,0.0666) | 0.0777 (0.0589,0.1018) | 0.0178 (0.0130,0.0209) |
| Mh | Virgin male | - | - | - | - | - |
|  | Mixed male | - | - | - | - | - |
| Sp:Dm | *try* New | - | - | - | - | - |
|  | *neo* New | - | - | - | - | - |
|  | *try* Old | - | - | - | - | - |
|  | *neo* Old | - | - | - | - | - |
| Sp:Mh | *try* Mixed male | - | - | - | - | 0.0030 (0.0014,0.0098) |
|  | *neo* Mixed male | - | - | - | - | 0.0608 (0.0362,0.0864) |
|  | *try* Virgin male | - | - | - | - | 0.0030 (0.0014,0.0100) |
|  | *neo* Virgin male | - | - | - | - | 0.0000 (0.0000,0.0016) |
| Dm:Mh | New Mixed male | - | - | - | - | - |
|  | Old Mixed male | - | - | - | - | - |
|  | New Virgin male | - | - | - | - | - |
|  | Old Virgin male | - | - | - | - | - |
| Sp:Dm:Mh | *try* New Mixed male | 0.0000 (0.0000,0.0007) | - | - | - | - |
|  | *neo* New Mixed male | 0.0161 (0.0152,0.0169) | - | - | - | - |
|  | *try* Old Mixed male | 0.0000 (0.0000,0.0007) | - | - | - | - |
|  | *neo* Old Mixed male | 0.0000 (0.0000,0.0007) | - | - | - | - |
|  | *try* New Virgin male | 0.0000 (0.0000,0.0007) | - | - | - | - |
|  | *neo* New Virgin male | 0.0000 (0.0000,0.0007) | - | - | - | - |
|  | *try* Old Virgin male | 0.0000 (0.0000,0.0007) | - | - | - | - |
|  | *neo* Old Virgin male | 0.0000 (0.0000,0.0007) | - | - | - | - |

**Supplementary Table S5**

| **Term** | **Category** | **867** | **939** | **981** | **1019** | **1026** | **1059** | **1094** | **1127** | **1132** |
| --- | --- | --- | --- | --- | --- | --- | --- | --- | --- | --- |
| Sp | *try* | - | - | - | - | - | 0.0002 (0.0001,0.0007) | - | 0.0000 (0.0000,0.0013) | - |
|  | *neo* | - | - | - | - | - | 0.0036 (0.0013,0.0050) | - | 0.0047 (0.0034,0.0060) | - |
| Dm | New | - | - | - | 0.0018 (0.0009,0.0030) | 0.1044 (0.0678,0.1592) | - | - | - | - |
|  | Old | - | - | - | 0.0055 (0.0038,0.0065) | 0.3037 (0.1762,0.4310) | - | - | - | - |
| Mh | Virgin female | - | 0.0008 (0.0004,0.0012) | - | 0.0017 (0.0009,0.0030) | 0.0603 (0.0515,0.1042) | - | - | - | - |
|  | Mixed female | - | 0.0045 (0.0033,0.0068) | - | 0.0055 (0.0038,0.0065) | 0.4545 (0.3148,0.6196) | - | - | - | - |
| Sp:Dm | *try* New | - | 0.0050 (0.0034,0.0107) | 0.0001 (0.0000,0.0002) | - | - | - | 0.0000 (0.0000,0.0004) | - | 0.0057 (0.0032,0.0165) |
|  | *neo* New | - | 0.0002 (0.0000,0.0006) | 0.0000 (0.0000,0.0001) | - | - | - | 0.0000 (0.0000,0.0004) | - | 0.0078 (0.0033,0.0187) |
|  | *try* Old | - | 0.0022 (0.0012,0.0039) | 0.0002 (0.0001,0.0003) | - | - | - | 0.0000 (0.0000,0.0004) | - | 0.0017 (0.0012,0.0026) |
|  | *neo* Old | - | 0.0037 (0.0022,0.0052) | 0.0026 (0.0009,0.0033) | - | - | - | 0.0025 (0.0021,0.0029) | - | 0.0507 (0.0396,0.0823) |
| Dm:Mh | New Mixed female | - | - | - | - | - | - | - | - | - |
|  | Old Mixed female | - | - | - | - | - | - | - | - | - |
|  | New Virgin female | - | - | - | - | - | - | - | - | - |
|  | Old Virgin female | - | - | - | - | - | - | - | - | - |
| Sp:Dm:Mh | *try* New Mixed female | 0.0004 (0.0002,0.0007) | - | - | - | - | - | - | - | - |
|  | *neo* New Mixed female | 0.0000 (0.0000,0.0003) | - | - | - | - | - | - | - | - |
|  | *try* Old Mixed female | 0.0000 (0.0000,0.0003) | - | - | - | - | - | - | - | - |
|  | *neo* Old Mixed female | 0.0035 (0.0032,0.0037) | - | - | - | - | - | - | - | - |
|  | *try* New Virgin female | 0.0000 (0.0000,0.0003) | - | - | - | - | - | - | - | - |
|  | *neo* New Virgin female | 0.0000 (0.0000,0.0003) | - | - | - | - | - | - | - | - |
|  | *try* Old Virgin female | 0.0000 (0.0000,0.0003) | - | - | - | - | - | - | - | - |
|  | *neo* Old Virgin female | 0.0000 (0.0000,0.0003) | - | - | - | - | - | - | - | - |

| **Term** | **Category** | **1142** | **1159** | **1172** | **1174** | **1185** | **1191** | **1201** | **1212** | **1235** |
| --- | --- | --- | --- | --- | --- | --- | --- | --- | --- | --- |
| Sp | *try* | 0.1606 (0.1106,0.2628) | - | - | - | - | 0.0000 (0.0000,0.0013) | - | - | - |
|  | *neo* | 1.2916 (0.7151,1.8351) | - | - | - | - | 0.0029 (0.0017,0.0042) | - | - | - |
| Dm | New | - | - | - | 0.0779 (0.0678,0.0865) | - | - | - | 2.3700 (1.8845,3.2187) | 0.0042 (0.0033,0.0088) |
|  | Old | - | - | - | 0.7824 (0.6503,1.0575) | - | - | - | 7.8379 (4.3507,10.5271) | 0.0216 (0.0094,0.0565) |
| Mh | Virgin female | - | - | - | - | - | - | - | - | - |
|  | Mixed female | - | - | - | - | - | - | - | - | - |
| Sp:Dm | *try* New | - | 24.9605 (19.6408,30.5611) | 0.0000 (0.0000,0.0031) | - | 0.0061 (0.0027,0.0090) | - | 0.1150 (0.0647,0.2597) | - | - |
|  | *neo* New | - | 19.0911 (13.6070,23.9906) | 0.0000 (0.0000,0.0031) | - | 0.0073 (0.0048,0.0101) | - | 0.0482 (0.0245,0.0737) | - | - |
|  | *try* Old | - | 15.7279 (8.6161,19.2724) | 0.0000 (0.0000,0.0031) | - | 0.0101 (0.0073,0.0157) | - | 0.0751 (0.0517,0.1378) | - | - |
|  | *neo* Old | - | 36.3940 (31.8749,38.5716) | 0.0195 (0.0193,0.0204) | - | 0.0000 (0.0000,0.0019) | - | 0.4550 (0.3480,0.5160) | - | - |
| Dm:Mh | New Mixed female | - | - | - | - | - | - | - | - | - |
|  | Old Mixed female | - | - | - | - | - | - | - | - | - |
|  | New Virgin female | - | - | - | - | - | - | - | - | - |
|  | Old Virgin female | - | - | - | - | - | - | - | - | - |
| Sp:Dm:Mh | *try* New Mixed female | - | - | - | - | - | - | - | - | - |
|  | *neo* New Mixed female | - | - | - | - | - | - | - | - | - |
|  | *try* Old Mixed female | - | - | - | - | - | - | - | - | - |
|  | *neo* Old Mixed female | - | - | - | - | - | - | - | - | - |
|  | *try* New Virgin female | - | - | - | - | - | - | - | - | - |
|  | *neo* New Virgin female | - | - | - | - | - | - | - | - | - |
|  | *try* Old Virgin female | - | - | - | - | - | - | - | - | - |
|  | *neo* Old Virgin female | - | - | - | - | - | - | - | - | - |

| **Term** | **Category** | **1239** | **1253** | **1267** | **1306** | **1327** | **1334** | **1340** | **1357** | **1370** |
| --- | --- | --- | --- | --- | --- | --- | --- | --- | --- | --- |
| Sp | *try* | - | - | - | - | - | - | - | - | - |
|  | *neo* | - | - | - | - | - | - | - | - | - |
| Dm | New | - | 0.0000 (0.0000,0.0012) | 0.0003 (0.0000,0.0007) | 0.0027 (0.0015,0.0038) | - | - | - | - | - |
|  | Old | - | 0.0058 (0.0038,0.0078) | 0.0046 (0.0027,0.0066) | 0.0504 (0.0239,0.0659) | - | - | - | - | - |
| Mh | Virgin female | - | - | - | - | - | - | - | - | - |
|  | Mixed female | - | - | - | - | - | - | - | - | - |
| Sp:Dm | *try* New | 2.1225 (1.1927,4.2754) | - | - | - | 0.0000 (0.0000,0.0009) | 0.0175 (0.0120,0.0376) | 0.0231 (0.0139,0.0443) | 0.4566 (0.3180,0.7470) | 0.0012 (0.0005,0.0028) |
|  | *neo* New | 1.1888 (0.7075,2.0894) | - | - | - | 0.0000 (0.0000,0.0009) | 0.0081 (0.0063,0.0141) | 0.0131 (0.0061,0.0194) | 0.3220 (0.1889,0.4583) | 0.0004 (0.0001,0.0010) |
|  | *try* Old | 1.6929 (1.1127,2.7540) | - | - | - | 0.0000 (0.0000,0.0009) | 0.0106 (0.0073,0.0169) | 0.0137 (0.0076,0.0206) | 0.2912 (0.1475,0.4386) | 0.0002 (0.0000,0.0010) |
|  | *neo* Old | 9.3242 (5.8351,11.7706) | - | - | - | 0.0034 (0.0024,0.0043) | 0.0615 (0.0444,0.0865) | 0.0788 (0.0575,0.1195) | 1.3592 (1.0018,1.7553) | 0.0108 (0.0046,0.0128) |
| Dm:Mh | New Mixed female | - | - | - | - | - | - | - | - | - |
|  | Old Mixed female | - | - | - | - | - | - | - | - | - |
|  | New Virgin female | - | - | - | - | - | - | - | - | - |
|  | Old Virgin female | - | - | - | - | - | - | - | - | - |
| Sp:Dm:Mh | *try* New Mixed female | - | - | - | - | - | - | - | - | - |
|  | *neo* New Mixed female | - | - | - | - | - | - | - | - | - |
|  | *try* Old Mixed female | - | - | - | - | - | - | - | - | - |
|  | *neo* Old Mixed female | - | - | - | - | - | - | - | - | - |
|  | *try* New Virgin female | - | - | - | - | - | - | - | - | - |
|  | *neo* New Virgin female | - | - | - | - | - | - | - | - | - |
|  | *try* Old Virgin female | - | - | - | - | - | - | - | - | - |
|  | *neo* Old Virgin female | - | - | - | - | - | - | - | - | - |

| **Term** | **Category** | **1375** | **1388** | **1461** | **1464** | **1473** | **1484** | **1492** | **1499** | **1525** |
| --- | --- | --- | --- | --- | --- | --- | --- | --- | --- | --- |
| Sp | *try* | - | - | - | - | 0.0036 (0.0013,0.0086) | - | 0.0027 (0.0013,0.0042) | - | - |
|  | *neo* | - | - | - | - | 0.0001 (0.0000,0.0002) | - | 0.0128 (0.0066,0.0156) | - | - |
| Dm | New | - | - | - | - | - | - | - | - | 0.1293 (0.1087,0.1654) |
|  | Old | - | - | - | - | - | - | - | - | 0.2879 (0.1955,0.4923) |
| Mh | Virgin female | - | - | - | - | - | - | - | - | - |
|  | Mixed female | - | - | - | - | - | - | - | - | - |
| Sp:Dm | *try* New | 0.0052 (0.0025,0.0066) | 0.0059 (0.0040,0.0097) | 0.0077 (0.0048,0.0142) | - | - | 0.0005 (0.0002,0.0010) | - | 0.0007 (0.0002,0.0027) | - |
|  | *neo* New | 0.0057 (0.0025,0.0068) | 0.0055 (0.0039,0.0082) | 0.0074 (0.0045,0.0132) | - | - | 0.0005 (0.0001,0.0010) | - | 0.0004 (0.0000,0.0016) | - |
|  | *try* Old | 0.0008 (0.0000,0.0025) | 0.0007 (0.0003,0.0040) | 0.0031 (0.0004,0.0068) | - | - | 0.0002 (0.0000,0.0005) | - | 0.0003 (0.0000,0.0012) | - |
|  | *neo* Old | 0.0592 (0.0363,0.0910) | 0.0227 (0.0109,0.0303) | 0.0368 (0.0220,0.0677) | - | - | 0.0023 (0.0014,0.0041) | - | 0.0122 (0.0067,0.0139) | - |
| Dm:Mh | New Mixed female | - | - | - | 0.0033 (0.0015,0.0047) | - | - | - | - | - |
|  | Old Mixed female | - | - | - | 0.0001 (0.0000,0.0011) | - | - | - | - | - |
|  | New Virgin female | - | - | - | 0.0068 (0.0060,0.0070) | - | - | - | - | - |
|  | Old Virgin female | - | - | - | 0.0000 (0.0000,0.0001) | - | - | - | - | - |
| Sp:Dm:Mh | *try* New Mixed female | - | - | - | - | - | - | - | - | - |
|  | *neo* New Mixed female | - | - | - | - | - | - | - | - | - |
|  | *try* Old Mixed female | - | - | - | - | - | - | - | - | - |
|  | *neo* Old Mixed female | - | - | - | - | - | - | - | - | - |
|  | *try* New Virgin female | - | - | - | - | - | - | - | - | - |
|  | *neo* New Virgin female | - | - | - | - | - | - | - | - | - |
|  | *try* Old Virgin female | - | - | - | - | - | - | - | - | - |
|  | *neo* Old Virgin female | - | - | - | - | - | - | - | - | - |

| **Term** | **Category** | **1592** | **1609** | **1675** | **1716** | **1723** | **1760** | **1783** | **1788** | **1981** | **1989** | **2188** |
| --- | --- | --- | --- | --- | --- | --- | --- | --- | --- | --- | --- | --- |
| Sp | *try* | - | - | 0.4791 (0.3511,0.5630) | - | - | 0.8493 (0.3517,1.1677) | - | - | - | - | 0.0689 (0.0453,0.0837) |
|  | *neo* | - | - | 0.1979 (0.1614,0.2606) | - | - | 0.1777 (0.1292,0.1998) | - | - | - | - | 0.0267 (0.0188,0.0382) |
| Dm | New | - | - | - | - | - | - | - | - | - | - | - |
|  | Old | - | - | - | - | - | - | - | - | - | - | - |
| Mh | Virgin female | - | - | - | - | - | - | - | - | - | 1.9283 (1.7531,2.0650) | 0.0679 (0.0393,0.0836) |
|  | Mixed female | - | - | - | - | - | - | - | - | - | 1.1978 (0.9504,1.4863) | 0.0270 (0.0195,0.0387) |
| Sp:Dm | *try* New | 29.7258 (26.0126,34.0551) | 0.0015 (0.0010,0.0191) | - | - | - | - | 3.2038 (2.4190,4.5000) | 23.1874 (19.3487,25.2768) | 6.3106 (4.5809,7.8934) | 1.9016 (1.7056,2.1581) | - |
|  | *neo* New | 28.6078 (25.2198,31.9391) | 0.0000 (0.0000,0.0005) | - | - | - | - | 7.8366 (5.2541,11.2460) | 25.2020 (23.1619,28.2549) | 8.9629 (7.7963,9.2968) | 1.7098 (1.5013,1.9267) | - |
|  | *try* Old | 31.9706 (28.6235,40.1628) | 0.0000 (0.0000,0.0005) | - | - | - | - | 5.3301 (4.1218,9.2790) | 22.9575 (17.3185,24.6018) | 8.2097 (6.5865,9.0966) | 2.0170 (1.7770,2.2789) | - |
|  | *neo* Old | 14.9240 (6.2424,22.7925) | 0.0000 (0.0000,0.0005) | - | - | - | - | 2.2881 (1.6039,3.1985) | 12.1199 (9.5445,13.7250) | 3.3082 (1.8810,4.8516) | 0.4776 (0.3203,0.8209) | - |
| Dm:Mh | New Mixed female | - | - | - | 0.0288 (0.0195,0.0426) | 0.6630 (0.5796,1.2839) | - | - | - | - | - | - |
|  | Old Mixed female | - | - | - | 0.0211 (0.0097,0.0328) | 0.4947 (0.3765,0.6561) | - | - | - | - | - | - |
|  | New Virgin female | - | - | - | 0.0105 (0.0056,0.0221) | 0.4719 (0.3544,0.6259) | - | - | - | - | - | - |
|  | Old Virgin female | - | - | - | 0.0469 (0.0297,0.1259) | 0.8198 (0.6295,2.6555) | - | - | - | - | - | - |
| Sp:Dm:Mh | *try* New Mixed female | - | - | - | - | - | - | - | - | - | - | - |
|  | *neo* New Mixed female | - | - | - | - | - | - | - | - | - | - | - |
|  | *try* Old Mixed female | - | - | - | - | - | - | - | - | - | - | - |
|  | *neo* Old Mixed female | - | - | - | - | - | - | - | - | - | - | - |
|  | *try* New Virgin female | - | - | - | - | - | - | - | - | - | - | - |
|  | *neo* New Virgin female | - | - | - | - | - | - | - | - | - | - | - |
|  | *try* Old Virgin female | - | - | - | - | - | - | - | - | - | - | - |
|  | *neo* Old Virgin female | - | - | - | - | - | - | - | - | - | - | - |
